# The Efficacy and Safety of Nutritional Supplements for Cancer Supportive Care: An Umbrella Review and Hierarchical Evidence Synthesis

**DOI:** 10.1177/15347354251405267

**Published:** 2026-01-03

**Authors:** Sarah Benna-Doyle, Suzanne Grant, Alison Maunder, Jing Liu, Melik Ibrahim, Adele Cave, Chhiti Pandey, Monica Tang, Eng-Siew Koh, Geoff Delaney, Deep Jyoti Bhuyan, Victoria Choi, Ki Kwon, Maria Gonzalez, Susannah Graham, Ashanya Malalasekera, Carolyn Ee

**Affiliations:** 1 NICM Health Research Institute, Western Sydney University, Australia; 2 South Western Sydney Local Health District, Sydney, Australia; 3 Ingham Institute for Health and Medical Research, Sydney, Australia; 4 South-Western Sydney Clinical School University of New South Wales, Sydney, Australia; 5 Liverpool Hospital, Sydney, Australia; 6 Chris O’Brien Lifehouse, Sydney, Australia; 7 Faculty of Medicine and Health University of Sydney, Sydney, Australia; 8 University of New South Wales, Sydney, Australia; 9 Sydney Local Health District, Sydney, Australia

**Keywords:** cancer supportive care, nutritional supplements, review, evidence synthesis

## Abstract

Cancer survivors experience a range of side effects during and after treatment. There is a need for a rigorous synthesis of the most recent and best available evidence on the role of nutritional supplements for supportive care in cancer, to inform shared decision-making. We searched 5 databases for umbrella reviews, meta-analyses and systematic reviews on nutritional supplements for supportive cancer care, excluding studies on pain, anxiety and depression, which are covered in recent guidelines. We found 52 reviews that reported on 250 RCTs on 18 supplements for 16 indications. Almost all reviews were of low/critically low quality (assessed using A MeaSurement Tool to Assess systematic Reviews version 2). There was moderate-certainty evidence for benefit from the following supplements: amino acids and oral proteolytic enzymes for severity of radiation-induced dermatitis, N-acetyl cysteine for prevention of chemotherapy-induced peripheral neuropathy (CIPN) in individuals with gastrointestinal cancers. There was low to very low certainty evidence that glutamine, zinc, probiotics and melatonin may be effective for oral mucositis; Vitamin E, omega-3 fatty acids, glutamine and other amino acids may be effective for preventing CIPN. Serious adverse events were reported for high-dose Vitamin A, and dose-related adverse events were reported with zinc and Vitamin E. However, the majority of nutritional supplements were associated with only minor adverse events. Due to the low to very low certainty of the majority of evidence, firm clinical recommendations cannot be made. Further research to conclusively evaluate benefit and harm, including potential impact on efficacy of standard treatments, should be conducted.

## Introduction

Globally, 1 in 5 people will be diagnosed with cancer in their lifetime.^1^ Advances in early detection, novel therapeutics and other aspects of clinical care have led to overall improvements in cancer survival.^2^ An estimated 53.5 million people worldwide are cancer survivors with a personal history of a cancer diagnosis within the last 5 years.^1,3^ Whilst the concept of survivorship is often associated with the period following treatment completion, a cancer survivor is defined as any individual diagnosed with cancer from the day of diagnosis until the end of life.^4^

Cancer survivors are a heterogeneous population with diverse and dynamic healthcare needs that vary along the cancer continuum.^5,6^ Their experiences and healthcare needs are often shaped by treatment side effects – the undesired yet often expected consequences of medical interventions.^7^ Acute side effects are most amplified during treatment, impacting quality of life (QoL) and treatment adherence.^8,9^ Although some side effects resolve post-treatment, many survivors face persistent and late-onset sequelae that can negatively impact well-being and contribute to long-term morbidity.^10,11^

Treatment-specific toxicities vary widely.^8^ Fatigue is the most prevalent, persistent and burdensome symptom affecting over 50% of survivors regardless of treatment modality.^8,12,13^ Chemotherapy is associated with nausea and vomiting (CINV; 30%-60%),^14,15^ oral mucositis (OM; 30%-90%)^16^ and peripheral neuropathy (CIPN; up to 70%),^8^ while radiotherapy-associated dermatitis (RD) affects up to 95% of recipients.^17^ Additional acute effects such as xerostomia, weight loss, hot flushes and insomnia vary in incidence and severity according to cancer type, stage, treatment regimen and performance status.^18^ While individual symptoms can be distressing and debilitating on their own, cancer survivors often undergo multimodality treatment with diverse regimens, and symptoms rarely occur in isolation.^9,18^

Advances in early detection, immunotherapy and targeted treatments have significantly improved outcomes and increased survival rates.^11,19^ However, these gains are accompanied by long-term challenges and latent effects of treatment. Targeted therapies may cause hypertension, skin toxicities and metabolic disturbances.^19,20^ In addition to acute side effects, long-term complications include lymphoedema, cardiotoxicity, cognitive impairment, sexual dysfunction, genitourinary complications and endocrine disruption encompassing weight gain and cardiometabolic diseases.^11^

National and international multidisciplinary organisations, such as the Multinational Association of Supportive Care in Cancer (MASCC),^21^ the European Society of Medical Oncology (ESMO) and the American Society of Clinical Oncology (ASCO)^22^ have developed evidence-based guidelines for supportive care management across the most common and debilitating side effects. However, the inclusion of nutritional supplement interventions – defined here as vitamins, minerals, phytochemicals, pre/probiotics and foods containing live cultures, fatty acids or amino acids provided in supplement form^23^- remains limited, largely due to the magnitude and heterogeneity of the available literature.

Despite widespread usage among cancer survivors^24^ and general perceptions of safety, current guidelines offer limited recommendations on nutritional supplements. The Society of Integrative Oncology (SIO), in collaboration with ASCO, has issued specific guidelines for the integrative management of anxiety, depression, pain management and fatigue, which include some guidance on supplement use.^25^ Yet, a comprehensive synthesis of evidence on the efficacy and safety of nutritional supplements across treatment-related side effects is lacking.

To support evidence-informed shared decision-making between clinicians and patients, a rigorous synthesis of the most current evidence is needed. This umbrella review aims to synthesise findings from existing umbrella reviews, meta-analyses and systematic reviews, to provide an evidence base on the efficacy and safety of nutritional supplements in managing cancer treatment–related side effects.

## Materials and Methods

An umbrella review, also known as an overview of reviews, is a novel form of evidence synthesis that aims to collate and assess evidence from multiple reviews on a specific topic and provide a synthesis of the current body of evidence.^26^

### Protocol Registration

This umbrella review was designed and reported in accordance with the Preferred Reporting Items for Overviews of Reviews (PRIOR) reporting guidelines.^27^ A protocol was developed a priori and prospectively registered on the Joanna Briggs Institute (JBI) systematic review register 24 April 2024.

### Search Strategy

A comprehensive search strategy was developed by CE, SG and SBD. Two authors (SBD, AO) conducted a systematic search of Medline, CINHAL and EMBASE and Cochrane Database of Systematic Reviews and Epistemonikos for relevant publications from January 2019 to May 2024, limited to the last 5 years to capture and synthesise the most recent evidence. All umbrella reviews, meta-analyses and systematic reviews with data from randomised controlled trials (RCTs) available in English were considered. The full search strategy is available in the Supplemental Material (Table S1). Titles and abstracts of all retrieved studies were screened in duplicate independently. All full text articles were screened in duplicate by 2 of the 6 reviewers (SBD, AO, SG, CE, ALM, CP or JL). Disagreements were resolved following discussion.

### Selection Criteria

The eligibility criteria for this review were defined according to the Population, Intervention, Control, Outcome, Study Design (PICOS) framework and shown in Table 1. Criteria were developed by our multidisciplinary team with expertise across oncology (medical and radiation), surgery, and supportive care. Given that there have been recent, comprehensive, evidence-based guidelines published on integrative oncology for anxiety, depression and pain management in cancer care,^28^ we excluded these outcomes from our review. Although gastrointestinal (GI) side effects are common, they were not included in this review, with the exception of mucositis, nausea/vomiting, postoperative ileus.

**Table 1.** Inclusion Criteria-PICOS Framework.

Population	Adults (≥18 y) with a cancer diagnosis using nutritional supplements for supportive care which may include:
	• Appetite
	• Body weight loss/gain
	• Cardiotoxicity
	• Chemo/radiotherapy induced nausea/vomiting
	• Cognitive changes
	• Dermatitis
	• Fatigue
	• Fear of cancer recurrence
	• Genitourinary complications
	• Hot flushes
	• Insomnia/sleep disorders
	• Lymphoedema
	• Mucositis
	• Neuropathy
	• Postoperative ileus
	• Quality of life
	• Side effects of endocrine therapy
	• Xerostomia
	We excluded adults with cancer-related cachexia
Intervention	Adjunctive or stand-alone treatment with nutritional supplements with any duration or frequency administered orally or topically. Nutritional supplement was defined as vitamins, minerals, phytochemicals, pre/probiotics and foods containing live cultures, fatty acids, or amino acids provided in supplement form for management of symptom and/or side effects.
	• We included nutritional supplements for symptom management rather than supplements integrated into treatment protocols for micronutrient replacement
	• Studies on food products alone, fortified food interventions, diet composition, or dietary patterns were excluded.
	• We included phytochemicals isolated from foods of food-like sources (eg, curcumin isolated from turmeric, inulin isolated from Jerusalem artichokes)
	• We excluded herbal medicine interventions, defined as whole plant materials (including herbal medicine extracts)
Comparisons	Any control, as long as there is a direct comparison of the intervention to a control
Outcomes	We included reviews that reported on the following side effects/symptoms from cancer and its treatments. Validated instruments (where available) for each outcome are ordered according to clinical relevance.
	• Appetite (anorexia symptoms scale of the 30-item European Organisation for Research and Treatment of Cancer Quality of Life Questionnaire-Cancer [EORTC QLQ C-30], Anorexia/Cachexia Subscale [A/CS] of the Functional Assessment of Anorexia/Cachexia [FAACT], and visual analogue scale VAS)
	• Body weight loss/gain (weight, Body Mass Index [BMI])
	• Cardiotoxicity (Left ventricular ejection fraction [LVEF], if not available, global longitudinal strain [GLS], if not available, Cardiac troponins and Natriuretic Peptides Quality of life)
	• Chemotherapy/radiotherapy induced nausea and vomiting (CINV/RINV) incidence and severity using Rhodes Index of Nausea, Vomiting and Retching (INVR), Common Terminology Criteria for Adverse Events (CTCAE), World Health Organisation (WHO) criterion, Eastern Society for Medical Oncology (ESMO) criterion. Acute CINV/RINV defined as within 24 h of treatment, delayed defined as >24 h to 7 d post treatment, anticipatory defined as before next cycle.
	• Dermatitis (Radiation Therapy Oncology [RTOG] and CTCAE score)
	• Fatigue (National Comprehensive Cancer Network [NCCN] single item, Brief Fatigue Inventory [BFI], MD Anderson Symptom Inventory [MDASI], Measure Yourself Concerns and Wellbeing [MYCaW], Patient Reported Outcome Measurement Information System, Fatigue- Short Form [PROMISF-SF], Edmonton Symptom Assessment Scale [ESAS], EORTC QLQ C-30)
	• Fear of cancer recurrence
	• Genitourinary syndrome of menopause (dyspareunia, vulvovaginal dryness, discomfort, or irritation)
	• Hot flushes (frequency/severity of vasomotor symptoms [VMS], distress, bother, or interference caused by VMS)
	• Insomnia/sleep disorders
	• Lymphoedema (circumference measurement, water displacement, bioelectrical impedance spectroscopy [BIS])
	• Mucositis (pain measured by VAS, opioid use and oral intake [opioid use and soft food intake days in first 28 d of treatment], CTCAE grade for severity)
	• Neuropathy (CIPN symptoms, impact of CIPN, balance and gait)
	• Poor cognitive function
	• Postoperative ileus (time to flatus, time to stool, time to solid diet tolerance, requirement of nasogastric tube, length of hospital stay [LOS])
	• Quality of Life (any validated tool)
	• Xerostomia (salivary gland flow, signs of hyposalivation, duration, overall impact)
	• Safety and adverse events to nutritional supplement interventions
	We excluded all other gastrointestinal side effects apart from postoperative ileus.
	Reviews evaluating nutritional supplementation with the intent of improving time to recurrence or survival were not included.
Study design	Only umbrella reviews, meta-analyses, or systematic reviews of RCTs which meet the following criteria:
	• Published since January 2019
	• Clear inclusion criteria
	• Reports a systematic search and data extraction procedure
	• Reports a relevant quality assessment of included studies e.g., Risk of Bias for randomised controlled trials in a systematic review/meta-analysis such as Cochrane Risk of Bias or Jadad scale; and A MeaSurement Tool to Assess systematic Reviews (AMSTAR)/Risk of Bias Assessment Tool for Systematic Reviews (ROBIS) or similar validated instrument for systematic reviews included in an umbrella review
	• English language
	Systematic reviews with non-randomised trials were only included if a separate subgroup analysis of data from randomised trials was performed. Published clinical guidelines were included if they contained an umbrella review or systematic review that fulfilled the above criteria.

There is little consensus on what is included and excluded in defining nutritional supplements.^23^ For purposes of this review, we have defined nutritional supplements as products with ingredients that may include vitamins, minerals, phytochemicals, pre/probiotics, and foods containing live cultures (eg, fermented dairy products), fatty acids or amino acids in supplement form. Probiotics were defined as “live microorganisms which when administered in adequate amounts confer health benefits” with inclusions classified according to the International Scientific Association for Probiotics and Prebiotics (ISAPP) consensus statement.^29^ For phytochemicals, we included isolated or standardised compounds administered in supplement form and excluded whole plant materials and/or unprocessed herbs used in their natural form, including extracts of whole plant materials, as we considered these to be herbal medicines rather than nutritional supplements.^30^

### Data Collection and Analysis

#### Hierarchal Evidence Gathering

Due to the broad scope of the research question and resource limitations, it was not practicable to extract and collate data from all eligible reviews retrieved. Another common methodological challenge with umbrella reviews is the problem of overlapping primary studies. Including all systematic reviews without consideration of the likelihood of significant overlap of primary studies introduces the risk of increasing the relative weight of each primary study if it is included more than once in synthesis. This reduces trustworthiness of the final result. There is no consensus on the ideal method of managing overlap.^31^ In this review, we use a hierarchical evidence approach to systematically gather data from the top tiers of the evidence. We have previously piloted this method successfully in our other reviews.^32-35^ Our methods are similar to those used in other umbrella reviews.^31^ Using this approach, 1 author (SBD) reviewed all studies deemed eligible for inclusion following full text review to identify the most recent and highest tier of evidence available. The hierarchical order was structured as follows: (1) Umbrella reviews; (2) Network meta-analyses; (3) Meta-analyses of double-blind, placebo-controlled RCTs; (4) Meta-analyses of RCTs; (5) Systematic reviews of RCTs.

Where multiple reviews with similar PICO criteria were identified, the review with the most recent search date was preferentially selected. In situations where both an umbrella review and systematic review and meta-analysis addressed the same clinical question, the umbrella review was prioritised for inclusion. If the systematic review and meta-analysis was more recent, the umbrella review was updated with the more recent data. Where there was uncertainty regarding the amount of overlap between reviews with a similar clinical question, we assessed primary study overlap by producing a citation matrix and calculating the Corrected Covered Area (CCA) previously described by Pieper et al.^36^ Where primary study overlap between reviews was high (>15%), the most comprehensive review (ie, the review that included the most RCTs) with the most recent search date was selected. Hierarchical selection through CCA calculations was performed by 3 authors (SBD, ALM, JL). The hierarchical selection of reviews for inclusion was verified by a second author (CE).

#### Data Extraction

Seven reviewers (SBD, AO, JL, MI, ALM, JL, CP) independently extracted data using a predefined data extraction tool developed in Microsoft Excel. All extracted data were verified by a second reviewer. The data extraction tool encompassed the PICO of included reviews, number of trials and participants, literature search date, population details, intervention and control details, weighted or standard mean differences and confidence intervals for outcomes (where available), risk of bias assessment and Grading of Recommendations Assessment, Development and Evaluation (GRADE)^37^ assessments (where available).

#### Quality Assessment of Included Reviews

Included reviews were assessed using the AMSTAR Version 2.0.^38^ The AMSTAR-2 instrument is a validated and well-accepted measure of the quality of systematic reviews. It consists of 16 items assessing domains such as comprehensiveness of the search, having a clearly defined research question and eligibility criteria and use of appropriate risk of bias assessment. Quality assessment was performed independently in duplicate by SBD, ALM, JL, AO or MI. Disagreements were resolved via discussion and a third reviewer (CE) if required. An overall rating of review quality ranging from “critically low” to “high” was given according to the number of critical and non-critical domains met by the included review.^38^

#### Certainty of Evidence

The GRADE approach^37^ was used to assess and report the certainty of the evidence (ie, confidence in effect estimate). Where authors of the included reviews performed a GRADE assessment for outcomes using a validated tool/measure, the assessment was extracted directly from the review. Where possible, all other GRADE assessments were conducted independently by 2 reviewers (ALM, JL). Due to resource limitations, it was not possible to conduct a GRADE assessment for every outcome. Where outcomes were reported by more than 1 outcome measure, GRADE assessments were only conducted for a single outcome measure using the list of pre-defined validated tools or outcome measures, which are ordered in Table 1 in terms of clinical importance as determined by our clinician-researcher team (oncologists, breast surgeons, etc.). GRADE assessments were conducted on the most highly ranked (in terms of clinically important) outcome measure that was reported. Discrepancies were resolved by discussion. Not all reviews reported sufficient information to conduct a GRADE, and GRADEs were not possible for reviews with 1 RCT in the outcome analysis.

## Results

### Search Results

**Figure 1.**
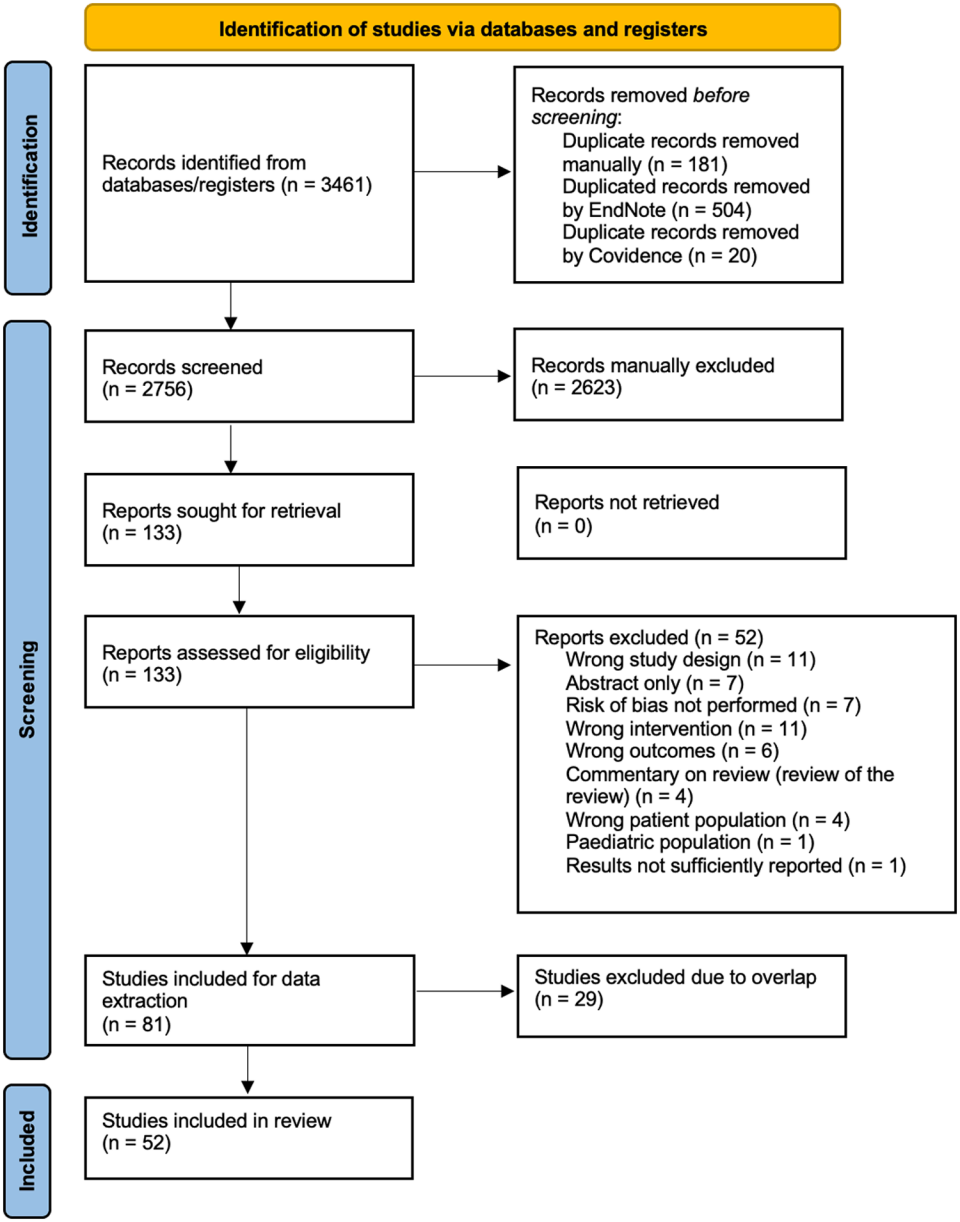
PRISMA flow chart.

The search results are presented in Figure 1. After duplicates were removed, the titles and abstracts of 2756 records were screened, and 2623 records that did not meet the inclusion criteria were excluded. The remaining full text of 133 records were assessed for eligibility, and 52 records that did not meet the inclusion criteria were removed following full-text review. A total of 81 studies were considered eligible for inclusion and subject to hierarchical evidence synthesis screening. Of the 81 eligible studies, 29 were subsequently excluded after checking for overlap, leaving 52 reviews included. The excluded studies with reasons for exclusion are listed in Supplemental Table S2.

### Study Characteristics

We report on 250 RCTs extracted from 1 umbrella review, 2 network meta-analyses, 25 meta-analyses and 24 systematic reviews, including 2 clinical practice guidelines.^39,40^ Of the 52 reviews evaluating the efficacy of nutritional supplements, 17 reviews evaluated QoL,^40-56^ 11 reviews evaluated OM,^41,45,53,56-63^ 10 reviews evaluated changes in body weight,^43,47,49,51,53,55,64-67^ eight reviews evaluated CIPN^40,63,64,68-72^, seven reviews evaluated dermatitis,^39,44,73-77^ six reviews evaluated fatigue^41,50,53,62,78,79^, five reviews evaluated xerostomia^45,51,62,63,80^, four reviews evaluated CINV^48,50,56,62^ and three reviews evaluated postoperative ileus.^67,81,82^ Other symptoms, including hot flushes,^63^ lymphoedema,^62,65^ genitourinary complications,^80,83^ sleep disorders,^41^ cardiotoxicity^84^ cognitive changes^47^ and appetite^48^ were less frequently investigated. No reviews evaluating the efficacy of nutritional supplementation for fear of cancer recurrence were identified.

The characteristics table (Table 2) provides a detailed overview of all outcomes evaluated in the 52 included reviews. Eighteen reviews reported on safety and adverse events related to the nutritional supplement intervention.^40,44,46,48,49,53,55,62-64,70,76,80,85-89^ Most of the reviews included populations with various cancer diagnoses (33 reviews), seven evaluated effects in individuals with breast cancer only, seven in gastrointestinal cancers only, four in reproductive cancers only (prostate, gynaecological) and one in head and neck cancer only.

**Table 2.** Characteristics of Included Studies.

Author (review type)	Number of participants (number of trials, relevant trials)	Relevant interventions (control type)	Outcomes	AEs to the nutritional supplement intervention
Search date	Inclusion criteria by PICO (Population, Intervention, Control, Outcome)		Treatment-related side effects (measurement tool/scale)	
Allenby et al^45^ (SR)	N = 1567 (16 RCTs; 3 RCTs on QoL, OM, xerostomia, n = 690) P = Patients with cancer undergoing radiotherapy I = Dietary modifications including nutritional supplementation C = Any control/usual diet O = Safety, radiotherapy toxicities, dermatitis, mucositis, xerostomia, QoL	Intervention(s): vitamin E + beta-carotene; antioxidants (beta-carotene + vitamin C/E + selenium); vitamin C + vitamin E Dose: NR Duration: During RT to 3 y post, during CT *(Placebo or usual care)*	OM (NR), QoL (EORTC QLQ-C30), xerostomia	NR
Inception to 2018				
Amiri Khosroshahi et al^57^ (UR)	N = 3514 (26 MAs, 18 RCTs on OM, n = 1271) P = Patients with cancer I = Nutritional interventions C = Placebo, basal oral care, no treatment O = OM incidence grade 3 or 4	Intervention(s) & dose: 800 mg/d vitamin E; 8 to 30 g/d glutamine Duration: 5 d to 156 wk *(Placebo, no treatment, or usual care)*	OM (NCI-CTC, WHO, RTOG, OMAS, NR) Any incidence or grade 3-4	NR
Inception to December 2021				
Amitay et al^81^ (MA)	N = 1318 (16 RCTs, 4 RCTs on postoperative ileus, n = 398) P = Patients with colorectal cancer undergoing/undergone colorectal resection I = Probiotics/Synbiotics C = placebo O = HRQOL, GI function, post-operative complications	Intervention: Pre/probiotics Strain(s)/dose: *Lactobacillus acidophilus, Bifidobacterium longum, L. plantarum, L.lactis, L.casei, B.bifidum, Saccharomyces boulardii, Enterococcus faecalis, B.infantis* Dose: NR Duration: 7-16 d, starting from 7 d before until 14 d after surgery *(Placebo)*	Postoperative ileus (time to first stool)	NR
Inception to January 2020				
An et al^85^ (MA)	N = 1763 (20 RCTs, 7 on AEs, n = 692) P = Patients with colorectal cancer undergoing surgery I = Any probiotic, synbiotic, prebiotic or combination of pre/probiotics C = Placebo or standard care without any other interventions O = post-operative outcomes including, mortality, infectious complications, and probiotic-related AEs as primary outcomes, LOS, QoL as secondary outcomes	Intervention: Pre/probiotics Strain(s)/dose: (i) *Lactobacillus acidophilus* NCFM + *L. rhamnosus* HN001 + *L. casei* LPC-37 *+* *Bifidobacterium lactis* HN019 + fructooligosaccharides (FOS); (ii) *B. longum* + *L. acidophilus* + *Enterococcus faecalis*; (iii) *B. animalis* + *B. lactis* + *L. casei + L. plantarum*; iv) lactic acid bacteria; v) *Streptococcus thermophilus* + *Bifidobacteria* + *L. acidophilus* + *L. plantarum* + *L. paracasei* + *L. delbrueckii subsp. Bulgaricus* Dose: NR Duration: 1-11 d before surgery to 21 d post-surgery. 1 d after antibiotic completion for 4 wk *(Placebo or standard care)*		AEs to probiotic intervention
Inception to Aug 2022				
Bagherniya et al^46^ (SR)	N = 483 (11 RCTs, 4 RCTs on AEs, 1 RCT on QoL, n = 222) P = Patients with prostate disease I = Curcumin supplementation C = NR O = NR	Intervention(s): curcumin, nanocurcumin Dose: 120 mg to 3000 mg/d Duration: from 7 d before RT, during RT; 6 wk to 6 mo *(Placebo)*	QoL (HRQOL)	AEs
Inception to April 2020				
Barnhart et al^80^ (SR)	N = 10 482 (24 RCTs, 4 RCTs on genitourinary atrophy, salivary gland function, AEs, n = 403) P = Oncology patients I = Vitamin C, vitamin E, selenium C = NR O = Safety and efficacy	Intervention(s): selenium ± lycopene, vitamin E ± vitamin C Dose: NR Duration: NR (*Placebo*)	Genitourinary atrophy, salivary gland function	AEs to selenium + lycopene supplementation
Jan 2016 to Jan 2022				
Behroozian et al^39^ (SR)	N = 5290 (1 39 RCTs, 2 RCTs RD, n = 88) P = patients with breast, head and neck, multiple cancer undergoing RT I = natural and miscellaneous agents C = placebo O = Dermatitis	Intervention(s): ascorbic acid solution 50 mcg/d, vitamin D ointment (Daivonex) Duration: NR During RD *(Placebo, or aqua cream)*	Radiodermatitis (RTOG, CNS Cancer Consortium)	NR
Inception to January 2023				
Belloni et al^79^ (SR)	N = 280 (3 RCTs, 3 RCTs on fatigue, n = 280) P = Adults (≥18) with cancer in active or post treatment phase I = probiotics, prebiotics, synbiotics C = Any active control or nonactive comparator group O = Cancer-related fatigue (CRF)	Intervention: Pre/probiotics Strain(s)/dose: *Lacidofil probiotic supplement* (BID); *Lactobacillus acidophilus* + *L. Casei* + *L. lactis* + *Bifidobacterium longum* + *B. infantis* (30 × 10^9^ CFUs per sachet QID) +2 g omega-3 FA/d; *L. rhamnosus, L. casei, L. acidophilus, L. bulgaricus, B. breve, B. longum, L. helveticus, L. lactis, L. paraplantarum, B. bifidum, Streptococcus thermophilus, and L. gasseri* (1 × 10^9^ CFUs capsule BID) +21 g prebiotic FOS Duration: 8 wk-6 mo before, during or after CT *(Placebo)*	CRF (FACT-F, EORTC-QLQ30, CFS)	NR
Inception to 2023				
Chang et al^73^ (MA)	N = 218 (5 RCTs, 5 RCTs on RD, n = 218) P = Patients with cancer experiencing RT- or CT-induced dermatitis I = Glutamine C = Control treatment or usual care O = Radiodermatitis	Intervention: 15-30 g glutamine/d; HMB/Arg/Gln (HMB 1.2 g, Arg 7 g, Gln 7 g) BID Duration: from 6 wk prior to CT/RT to 2 wk post *(Dextrose, usual care, glucose solution, maltodextrin, or placebo)*	Radiodermatitis (RTOG, CTCAE)	AEs to glutamine supplementation
Inception to March 2023				
Chen et al^69^ (MA)	N = 486 (9 RCTs, 9 RCTs on CIPN, n = 486) P = Patients with cancer undergoing CT I = Vitamin E C = NR O = CIPN Incidence (PNP, CTCAE), incidence of severe CIPN, and total neuropathy scores	Intervention: 300-600 mg Vitamin E/D Duration: Prior to CT and at the completion of CT; timeframe NR *(NR)*	Incidence of all-grade CIPN and severe CIPN (PNP, CTCAE).	NR
Inception to December 2020				
Cintoni et al^47^ (SR)	N = 553 (9 RCT, 1 RCT on BMI, QoL, n = 72) P = patients with pancreatic cancer undergoing CT I = nutritional interventions C = any comparison O = CT-related toxicity, body weight, QoL, survival	Intervention: oral liquid formulation of L-carnitine Dose: NR Duration: 12 wk *(Placebo)*	BMI, cognitive function (EORTC QLQ-PAN26), QoL (global health status)	NR
Inception to December 2022				
Cogo et al^82^ (SR)	N = 3305 (36 RCTs, 7 RCTs on postoperative ileus, n = 725) P = People with cancer undergoing surgery I = Synbiotic or probiotics C = Active control, placebo, usual care O = disease progression/stability, mortality, anthropometrics, cancer biomarkers, immune cells, inflammatory markers, LOS, postoperative infections, antibiotic use, complications.	Intervention: Probiotics/synbiotic Strain(s)/dose: *Saccharomyces boulardii* 50 × 10^6^ CFUs QID; *Bifidobacterium bifidum + B. infantis + Lactobacillus acidophilus + L. casei + L. salivarius + L. lactis* 10 × 10^9^ BID; *L. casei* Shirota 40 × 10^9^ + *B. breve* Yakult 10 × 10^9^ + 2.5 g GOS QID; *L. plantarum 299 v* 100 × 10^9^ CFUs in oatmeal-based drink 100 ml QID, *L. casei* Shirota 1 g + *B. breve* Yakult 1 g + GOS 15 g QID; *B. animalis* subsp. *L. lactis* HY8002 100 × 10^6^ CFU) + *L. casei* HY2782 50 × 10^6^ CFU + *L. plantarum* HY7712 50 × 10^6^ CFU + xylooligosaccharides 350 mg + FOS 36 mg BID; *B. longum* + *L. acidophilus + Enterococcus faecalis* 63 × 10^6^ CFUs TID Duration: 3-28 d *(Standard care, no treatment, or placebo)*	Postoperative ileus (intestinal obstruction)	NR
Inception to September 2020				
Crichton et al^68^ (MA)	N = 1012 (13 RCTs; 1 RCT on CIPN, n = 44) P = Patients with advanced cancer (any age) treated with CT I = Any non-pharmacological self-management intervention O = Incidence or severity of CIPN	Intervention: 30 g/d L-glutamine Duration: Every 2 wks for 7 d during CT *(Standard care)*	CIPN (NR)	
Inception to February 2022				
Croisier et al^48^ (SR)	N = 89 (4 RCTs, 3 RCTs on RINV, appetite, QoL, AEs, n = 79) P = Adults with gynaecologic cancer undergoing pelvic radiotherapy I = Nutrition intervention with pre/probiotics C = Standard care, placebo, or no treatment O = GI toxicity symptoms including bowel changes, abdominal pain, bloating, anorexia, CRINV, QoL	Intervention: Prebiotics Strain(s)/dose: 6 g prebiotic mixture (50% FOS + 50% inulin) BID; 150 ml yoghurt with 6.5% lactulose + 2 × 10^9^ *Lactobacillus acidophilus*/d; hydrolysed rice bran 3 g TID Duration: 5-7 wk, starting 5-7 d pre-RT *(No treatment, or placebo)*	RINV (study-specific tool), QoL (EORTC QLQ-C30)	AEs to intervention with fibre + pre/synbiotics
				Inception to December 2019
da Anunciacao et al^49^ (SR)	N = 450 (6 RCTs; 3 RCT on QoL, weight, AEs, n = 196) P = Patients with various cancer I = Turmeric supplementation C = Any therapy O = Dermatitis, QoL, body weight	Intervention: Curcumin Dose: 1-3 g curcumin capsules/d Duration: During RT (16-33 sessions), RT/CT or CT *(Control, dicalcium phosphate, rice bran)*	Body weight, QoL (University of Washington QoL), RD (RDSS)	AEs to curcumin supplementation
Inception to Aug 2017				
Deleemans et al^50^ (SR)	N = 974 (12 RCTs, 6 RCTs on QoL, CINV, fatigue, n = 457) P = People with cancer undergoing treatment or survivors finished treatment I = Prebiotics, probiotics C = NR O = Nausea, vomiting, bloating, acid reflux, heartburn, GERD, dyspepsia, indigestions, depression, anxiety, fatigue, pain, cognitive decline, social functioning, fear of recurrence, emotional wellbeing, mood	Intervention: Prebiotics/probiotics Strain(s)/dose: Synbiotic biogel with *Lactobacillus acidophilus* NCFM 1 × 10^7^ CFU/d + *Bifidobacterium lactis* Bi-07 1 × 10^6^/d + inulin TID; “Bifilact” *L. acidophilus* LAC – 361 + *B. longum* BB-536 BID 1.3 × 10^9^ CFU BID or 10 × 109 CFU TID; “Lacidofil” *L. rhamnosus* R0011, *L. acidophilus* R0052 2 × 10^9^ CFU BID; PHGG BID; fibre-enriched jelly “jevity FOS” with 10 g fibre,7 g FOS/L; *L. plantarum* CJLP243 (KCCM11045P), isolated from kimchi 10 × 10^9^/d Duration: 3-12 wk *(Placebo, or standard western diet)*	CINV (frequency/intensity), QoL (EORTC-QLQ, FACT-C, GIQLI), fatigue (FACT-F)	NR
Inception to September 2021				
Delmicon et al^51^ (SR)	N = 463 (5 RCTs; 3 RCT on weight, CIPN, xerostomia n = 166) P = Women undergoing breast cancer treatment I = Supplementation with omega-3 fatty acids (EPA/DHA) C = Placebo O = Adverse effects of cancer treatment including xerostomia, neuropathy, mucositis, nausea, vomiting, diarrhoea, lack of appetite, fatigue, increased bone resorption, arthralgia, and maintenance of lean body mass	Intervention: 2.24 g EPA + 1.12 g DHA/day; 1.6 g EPA + 0.8 DHA/day, 1.04 g DHA + 0.2 g EPA/d Duration: 8-24 wk *(Placebo)*	Arthralgia [Brief Pain Inventory (BPI-SF) scale], Body weight, PIPN (NR), QoL (FACT-ES), Xerostomia (Edmonton scale)	NR
Inception to December 2022				
Dikeocha et al^52^ (SR)	N = 2457 (23 RCTs; 3 RCTs on QoL, n = 175) P = Patient with colorectal cancer post-surgery I = Probiotics ± prebiotics. C = Active control or placebo O = Effect on microbiome, immune and inflammatory biomarkers, postoperative complications, LOS, death, tumour size	Intervention: Pre/probiotics Strain(s)/dose: Alfasigma capsule 112.5 × 10^9^ CFU BID containing *B. infantis, L plantarum, L paracasei, B. longum, L. bulgaricus, L. acidophilus, B. breve, and Streptococcus thermophilus)*; 1 sachet MCP preparation (30 × 10^9^ CFU + omega-3 FAs/d); FourLAB: *Pediococcus pentosaceus, Leuconostoc mesenteriodes, Lactobacillus paracasei, L. plantarum* *+* b-glucan, inulin, pectin, resistant starch 12 g/day Duration: 15 d-4 wk; postoperatively for ≤15 doses or until discharge (Placebo, or no treatment)	QoL (FACT, EORTC-QLQ-C30, GIQLI)	NR
Inception to January 2020				
E Vasconcelos et al^76^ (SR)	N = 2416 (17 RCTs; 5 RCTs RD, AEs, n = 971) P = Patients with cancer undergoing RT I = Oral supplementation for management of RD C = None or any intervention O = Prevention or reduction of the grade of severity of RD	Intervention(s): 500 mg Adlay Bran extract capsules QID; 125 mg anthocyanins TID; 400 IU a-tocopherol ± 30 mg b-carotene/day; 24 g HMB/Arg/Gln (1.2 g HMB, 7 g Arg, 7 g Gln) BID; 25 mg zinc TID Duration: 1 wk prior to treatment with RT to 3 y following treatment completion *(Placebo, olive oil capsule, no treatment, or soya bean oil capsule)*	RD (RTOG, CTCAE)	AEs to nutritional supplement interventions
Inception to May 2022				
Fan et al^41^ (MA)	N = 2101 (19 RCTs, 19 RCTs on fatigue, QoL, sleep, stomatitis, n = 2101) P = Adults with cancer I = Melatonin C = NR O = QoL, sleep quality, fatigue, depression, pain, stomatitis	Intervention(s): 3-20 mg melatonin/day or 3% melatonin gel Duration: 7 d to 1 y, or until death (P*lacebo, no treatment, or light therapy*)	Fatigue (EORTC, FACT-F, MFI-20, VAS), QoL (EORTC, FACT-L), sleep (AIS, EORTC, ISI, KSS, MOS, PSQI), stomatitis (WHO, NR)	NR
Inception to November 2021				
Feng et al^59^ (MA)	N = 1013 (12 RCTs, 7 RCTs on OM, n = 647) P = People with cancer undergoing chemotherapy I = Probiotics C = Patients that did not take probiotics O = OM incidence	Intervention: Probiotics Strain/dose: 1 capsule BID 109 CFU *L. plantarum* MH-301, *B. animalis subsp. Lactis* LPL-RH, *L. rhamnosus* LGG-18, *L. acidophilus;* 3 capsules BID *Bifidobacterium longum, Lactobacillus lactis, Enterococcus faecium*; 3 g 108 CFU/day Yakult (*Bifidobacterium breve* strain Yakult, *Lactobacillus casei* strain Shirota, GOS*)*; 2 × 109 CFU/lozenge, 6 lozenges/day *Lactobacillus brevis* CD 2; 1-2 capsule(s)1010 CFU BID *Lactobacillus rhamnosus* GG Duration: 2 d prior to CT to end of CT for 24 wk *(Placebo, or usual care)*	Incidence and severity of OM (CTCAE, RTOG)	
Inception to December 2021				
Gremmler et al^62^ (SR)	N = 671 (10 RCTs, 7 RCTs on, OM, RINV, fatigue, AEs, lymphoedema, QoL, n = 481) P = Adult patients with cancer (≥18 y) I = proteolytic enzyme therapy (containing papain, trypsin, chymotrypsin) C = Any comparison (observation, standard care, placebo) O = Patient-relevant symptoms/toxicities, response data, survival data, QoL	Intervention: 5-15 tablets tablets Wobe-Mugos^®^/d; 5 Wobenzym^®^ tablets TID, 10xWobe-Mugos^®^ dragees TID Duration: 2-6 d prior to RT, during RT for 45-68 d; day 2-7 of every CT cycle *(Placebo)*	OM (EORTC, RTOG NR), dysphagia (EORTC, RTOG), RINV (frequency), fatigue (CTCAE, EORTC), QoL (NR), lymphoedema (volume)	AEs to Wobe-Mugos^®^ enzyme supplementation
Inception to May 2019				
Hoppe et al^53^ (SR)	N = 1180 (23 RCTs, 2 RCTs on OM, fatigue, QoL, BMI, n = 107) P = Adults with cancer treated with CT, surgery or RT I = Zinc C = Standard care, observation, placebo O = Mucositis, xerostomia, altered taste, oral pain, mucosal inflammation, survival data, QoL, fatigue, immune response, zinc toxicities	Intervention: Zinc Dose: NR Duration: NR (*Placebo)*	BMI, fatigue (NR), OM (NR), QoL (NR)	AEs to zinc supplementation
Inception to January 2019				
Lam et al^64^ (MA)	N = 1744 (28 RCTs, 13 RCTs, weight, AEs n = 691) P = Adults with any cancer, undergoing any treatment with any nutritional status *I* = 600 mg or more of oral omega-3 (EPA or DHA) supplementation C = Placebo, usual care O = Body composition, QoL, body weight, adverse events	Intervention: 360 mg-2.20 g EPA + 240 mg-1.6 g DHA O3-FA capsules Duration: 30 d-6 mo *(Placebo or isocaloric oral nutritional supplement)*	Body weight (NR)	AEs to O3-FA supplementation
Inception to December 2019				
Lee et al^74^ (MA)	N = 591 (8 RCTs, 7 RCTs on RD = 458) P = Patients with breast cancer receiving RT I = HA, topical C = NR O = Dermatitis grade	Intervention: Topical HA + beta glucan Duration: 0-15 d before RT, during RT, 30-90 d after RT *(Alginate, Avene thermal water, emollients, grapevine extract, omega 3, 6, 9, petrolatum-based or placebo cream, phytosterols, or vitamin E)*	RD (RTOG, NCI)	NR
Inception to March 2021				
Leen et al^70^ (MA)	N = NR (24 RCTs; 2 RCTs on CIPN, AEs n = 108) P = Adults with cancer treated with paclitaxel CT I = Pharmacological or non-pharmacological interventions C = Placebo or control O = Incidence of paclitaxel-induced peripheral neuropathy (PIPN)	Intervention: NAC, glutamate Dose: NR Duration: 3-12 wk *(Placebo)*	Incidence of PIPN (FACT/Gynaecologic Oncology Group-NTX subscale)	AEs to NAC supplementation
January 2000 to September 2021				
Lin et al^42^ (MA)	N = 3010 (34 RCTs; 6 RCTs on QoL n = 280) P = Patients with cancer I = Complementary and integrative medicine C = No intervention for psychological wellbeing or QoL O = QoL	Intervention(s): L-carnitine 0.5 g/d (Day 1-2), 1 g/d (Day 3-4), 2 g/d ongoing; Biorinck chlorella granules 4 sticks/d; CoQ10 300mg + vitamin E 300 IU TID; creatine monohydrate 20 g/d (first week), 5 g/d ongoing; kefir 250 ml BID; 4.3 g/d EPA + DHA Duration: NR (*NR*)	QoL (FACT, FACIT, MDASI, SF-36, EORTC)	NR
Inception to February 2018				
Liu et al^90^ (NMA)	N = 2349 (16 RCTs, 1 RCT on hot flushes, n = 358) P = Patients with breast cancer experiencing hot flashes I = Non-hormonal therapies C = Hormonal therapies, no treatment/waitlist, placebo/sham treatment O = Hot flash frequency, hot flash scores, AEs	Intervention(s): 800 mg to 1200 mg/d magnesium oxide Duration: 8-12 wk (*Placebo)*	Hot flushes (Hot flush diary)	
Inception to May 2018				
Liu et al^43^ (MA)	N = 1556 (19 RCTs; 6 RCTs on body weight, BMI, QoL, n = 406) P = Adults with colorectal cancer undergoing surgery ± CT I = Omega 3 FAs C = NR O = Weight, infection rates, hospital stays, mortality, inflammation markers, BMI, QoL	Intervention: 108 mg EPA QID to 1.6 g/day Duration: NR *(NR)*	Body weight, BMI, QoL (NR)	NR
Inception to March 2023				
Liu et al^60^ (MA)	N = 783 (12 RCTs; 12 RCTs on OM) P = Patients undergoing CT and/or RT I = Zinc in any form C = Placebo or control O = OM incidence	Intervention(s): Oral zinc sulphate, zinc chloride mouthwash, oral polapre-zinc rinse Dose: 30- 220 mg BID to QID Duration: 28 d to 20 wk or 30-35 treatment sessions *(Placebo)*	OM (RTOG, WHO)	NR
Inception to December 2021				
Loprinzi et al^40^ (SR)	N = 4252 (31 RCTs, 5 RCTs on CIPN, AEs, n = 440) P = Patients with cancer undergoing CT I = Nutritional interventions C = Any comparison O = CIPN	Intervention(s): 1800 mg alpha-lipoic-acid/day, 300 to 3000 mg acetyl-l-carnitine/day, 1 capsule BID B vitamin complex (50 mg thiamine, 20 mg riboflavin, 100 mg niacin, 163.5 mg pantothenic acid, 30 mg pyridoxine, 500 mcg folate, 500 mcg cyanocobalamin, 500 mcg biotin, 100 mg choline, and 500 mcg inositol) Duration: During CT *(Placebo or NR)*	CIPN (FACT-NTX, PNQ); QoL (EORTC QOL)	AEs to vitamin E supplementation only
January 2013 to February 2020				
Mahdavi et al^54^ (SR)	N = 234 (3 RCTs, 1 RCT on QoL, n = 38) P = Patients with colorectal cancer I = Probiotics, synbiotics C = Placebo O = Chemotherapy-toxicity	Intervention: Probiotics Strain/dose: BID 108 CFU *Lactobacillus casei* PXN 37, *L. rhamnosus* PXN 54, Streptococcus thermophilus PXN 6635, *Bifidobacterium breve* PXN25, *L. acidophilus* PXN 35l, *B. longum* PXN 30, *L. bulgaricus* PXN 39 + FOS, magnesium stearate Duration: 6 wk (Placebo)	QoL (EORTC QLQ-C30)	NR
Inception to January 2021				
Momenzadeh et al^71^ (MA)	N = 858 (3 RCTs, 3 RCTs on CIPN, n = 858) P = Women with breast cancer receiving taxane CT I = Acetyl-L-carnitine C = Standard care O = Improvement in CIPN	Intervention: 3 g/day acetyl-L-carnitine Duration: 56-168 d *(NR)*	PIPN (FACT-NTX)	NR
January 2010 to December 2019				
Moustafa et al^84^ (SR)	N = NR (12 RCTs, 6 RCTs on cardiotoxicity, n = 186) P = Adults with cancer treated with doxorubicin I = Vitamin E and levocarnitine alone or in combination C = Any comparison O = Cardiotoxicity	Intervention(s): Vitamin E, L-carnitine Dose: Vitamin E, 400 mg-600 mg/d, 800 IU 1800 IU; L-carnitine 3 g before CT, 1 g during CT Duration: Before and during CT (*No intervention, placebo, digoxin, silymarin or NR*)	Cardiotoxicity	NR
Inception to April 2021				
Nogueira et al^44^ (SR/MA)	N = 383 (7 RCTs; 4 RCTs for Vitamin E, n = 228) P = Adults with breast cancer. (any stage of disease) treated with RT exclusively or in combination with radiation-induced fibrosis I = Any type of treatment to improve or resolve the radiation-induced fibrosis C = Any other intervention, placebo, no intervention O = Incidence of fibrosis, intervention-related AEs, QoL	Intervention: 1000- 12 000 mg IU vitamin E per day Duration: 6 mo *(Placebo or no intervention)*	Fibrosis (LENT-SOMA scoring scale), QoL (EORTC QLQ-C30, BR23)	AEs to vitamin E supplementation
NR				
Pandy et al^77^ (MA)	N = 2814 (16 RCTs; 8 RCTs on pyridoxine, n = 1031) P = Adult cancer patients receiving systemic cancer treatment I = Urea cream or pyridoxine or celecoxib as prophylaxis C = Placebo or another treatment for Hand Foot Syndrome/Hand Foot Skin Reaction O = Rates of HFS/HFSR of any grade	Intervention: 60-200 mg/day pyridoxine Duration: NR *(Placebo or no pyridoxine or Eppikajutsuto (Kampo medicine))*	Hand Foot Syndrome (Grade 1-3)	AEs to pyridoxine supplementation
Inception to August 2021				
Peng et al^72^ (SR/MA)	N = (x RCTs, 5 RCTs on CIPN, n = 253) P = Adults with cancer with planned oxaliplatin as the only neurotoxic chemotherapeutic agent I = Any form of protective measure to prevent and/or reduce the incidence of chronic OIPN C = Placebo, no intervention, other interventions O = Incidence and/or severity of chronic OIPN measured using clinical scales or electrophysical studies	Intervention(s): 500-1000 mg/d L-carnosine, 30 g/d glutamine, 1200 mg/d NAC Duration: During CT (*Placebo, non-active intervention)*	OIPN (CTCAE)	NA
August 2020				
Prado et al^55^ (SR)	N = 416 (5 RCTs, 5 RCTs on weight, BMI, QoL, AEs, n = 375) P = Adults with cancer of (any type/stage) I = HMB combined with amino acids C = Placebo, standard care O = Muscle mass, HRQoL, body weight, muscle function, physical performance, sarcopenia, inflammation markers, hospitalisation rate, length of hospital stay, mortality and survival, adverse events	Intervention(s): 3 g HMB/Arg/Gln/d; Duration: 8 to 24 wk; 3 d pre surgery to 7 d post-surgery *(Isonitrogenous isocaloric placebo, standard care, routine nutritional care)*	QoL (NR), weight loss	AEs to supplementation to HMB
Inception to December 2021				
Raza et al^61^ (MA)	N = 650 (13 RCTS, 1 RCT on OM, n = 71) P = Patients with head and neck cancer undergoing RT with/without CT I = Oral or topical antioxidants C = Placebo O = OM incidence (WHO, CTCAE, OMAS, RTOG)	Intervention: 400 mcg selenium/d Duration: During RT period *(Placebo)*	OM (NR)	NR
Inception to February 2021				
Retzlaff et al^63^ (SR)	N = 1923 (5 RCTs on OM, salivary gland function, CIPN, hot flushes, n = 299) P = Adult cancer patients I = α-tocopherol C = Any control O = Toxicities, response data, survival data, QoL	Intervention/dose: α-tocopherol or vitamin E, 400 IU (≙268, 46 mg)-800 mg per oral tablet or topical application vitamin E/d Duration: commencing 4 d prior to CT/RT treatment up to 3 mo post CT and 3 y post RT (*Usual care, placebo, low dose of α-tocopherol,acetyl-L-carnitine, glutamine, methylocabalamine*)	CIPN (NR), hot flushes (frequency & severity), OM (NR), salivary gland function	NR
Inception to July 2020				
Retzlaff et al^87^ SR (SR)	N = 4296 (2 RCTs on vitamin A toxicity, n = 401) P = Adult patients with cancer I = vitamin A C = All possible control groups (placebo, standard care, observation) O = All patient-relevant symptoms/ toxicities, response data, survival data, QoL	Intervention: Busulphan + vitamin A 50 000 IU/d; vitamin A 100 000 IU/d Duration: 18 mo; 7 y *(Bulsulphan,or no treatment)*	NA	AEs to supplementation with vitamin A
Inception to November 2020				
Robijns et al^75^ (SR/MA)	N = 2849 (16 RCTs; 2 RCTs on RD, n = 219) P = Patients with cancer undergoing RT I = Any intervention C = Standard of care, placebo, any other intervention, or no intervention O = Acute RD	Intervention: oral enzymes (100 mg papain, 40 mg trypsin, 40 mg chymotrypsin) QID Duration: During RT to 1-wk post RT *(Placebo capsule/gel, Johnson baby oil, no treatment)*	RD (RTOG/EORTC/RDSS)	NR
Inception to January 2023				
Seo et al^86^ (SR/MA)	N = NR (9 RCTs; 4 RCTs on AEs, n = 233) P = Adults with breast cancer (excl. pregnant women) I = Melatonin supplements C = Not specified O = Sleep indicators	Intervention: 3-20 mg oral melatonin, daily Duration: 10 d to 4 mo *(Placebo)*		AEs to melatonin supplementation
Inception to February 2022				
Thu et al^65^ (SR/MA)	N = 571 (13 RCTs; 5 RCTs on weight, lymphoedema, n = 602) P = Adults with breast cancer I = Any probiotic treatment with or without any active treatment C = Active control or placebo O = QoL, alterations in bacteria profile, diversity, and metabolites	Intervention: Probiotics Dose/strain: ProLBE (*Lactobacillus with Bifidobacterium, Enterococcus*) 3 capsules (0.84 g) 2 times/day; ProLBS (*Lactobacillus with Bifidobacterium, streptococcus*) + FOS 1 capsule (109 CFU) + 38.5 g FOS)/d; ProLB (*Lactobacillus with Bifidobacterium*) 1 sachet (4 × 109 CFU)/d Duration: 3 wk *(Calorie restricted diet + placebo, Mediterranean diet only, placebo)*	Body weight (loss), BMI (reduction) lymphoedema (oedema volume),	NR
Inception to Mar 2022				
Tsai et al^78^ (SR/MA)	N = 1126 (13 RCTs; 1 RCT on fatigue n = 139) P = Human participants I = Coenzyme Q10 C = Placebo O = Fatigue	Intervention: 300 mg CoQ10/d Duration: 24 wk *(Placebo)*	Fatigue (Profile of Mood States fatigue subscale (0-4))	NA
Inception to January 2022				
Umlauff et al^66^ (SR/MA)	N = 536 (3 RCTs on BMI, n = 140) P = Men with prostate cancer treated with ADT I = Dietary interventions aimed to improve body composition, either direct (e.g., nutritional supplementation) or indirect (e.g., nutrition advice) manipulation of dietary intake C = Placebo or no dietary intervention O = Objective measure of body composition including lean mass, fat mass or BMI	Intervention/dose: whey protein with 2.4 g leucine powder + 1200 mg calcium carbonate and 1000 IU vit D/day; 25 g whey protein isolate (EnergyFirst Manhattan Beach, CA) with 4 g leucine BID; 20 g soy protein powder (Revival, Physicians Pharmaceuticals) with 160 mg isoflavones (64 mg genistein, 63 mg daidzein, 34 mg glycitein)/d Duration: 12-52 wk *(Usual care; home based flexibility programme, or placebo)*	BMI	NA
Inception to December 2020				
vanGorkom et al^88^ (SR)	N = NR (1 RCT on AEs, n = 100) P = Patients with cancer (all ages/cancer types) I = Vitamin C O = Overall survival, progression-free survival, tumour response, response rate, disease-free survival, AEs, QoL, clinical response and performance status	Intervention: 10 g oral vitamin C/D Duration: NR *(Placebo)*	NA	AEs to supplementation with vitamin C
March 2019				
Wang et al^58^ (NMA)	N = (1 RCT on vitamin E mouthwash, n = 59) P = Patients with OM caused by radio/chemotherapy I = Mouthwash C = Placebo or other agents O = OM severity score	Intervention: Vitamin E mouthwash Duration: 3 wk (*Placebo)*	Grade III/IV OM (WHO)	
April 2022				
Wierzbicka et al^83^ (SR)	N = 376 (3 RCTs, 2 RCTs genitourinary complications, n = 222) P = Adult women with gynaecological cancer treated with brachytherapy /radiotherapy I=hyaluronic acid, vitamin A, vitamin E C = Not specified O = Mucosal inflammation, dyspareunia, vaginal dryness, vaginal bleeding, vulvar fibrosis	Intervention: 5 mg HA, 1 mg vitamin C, 1 mg vitamin E Duration: 5-16 wk *(NR)*	Genitourinary side effects of pelvic RT	NR
October 2020 to November 2020				
Wu et al^56^ (SR)	N = 118 (4 RCTs, 1 RCT on QoL, OM, CINV n = 54) P = Adults with cancer undergoing treatment I = Fucoidan (seaweed compound) C = None, usual care, or placebo O = Disease progression, anti-inflammatory, nutritional status, adverse effects, and QoL	Intervention: 4 g fucoidan low molecular weight powder, BID Duration: 6 mo *(Cellulose powder)*	QoL (EORTC QLQ-CR29), OM (NR)	AEs to supplementation with fucoidan (NCI CTCAE and EORTC-QLQ-CR29)
Inception to November 2020				
Ye et al^67^ (MA)	N = 861 (9 RCTS; 8 RCTs on time to flatus, time to defecation, weight, n = 769) P = Patients with gastric cancer who had surgery I = Probiotic supplementation C = Placebo or standard diet O = Infectious complications, time to flatus, time to defecation, weight loss	Intervention: Strain(s)/dose:500 mg *Saccharomyces cerevisiae* Hansen CBS 5926, 4-6 g *Bifidobacterium* + *Lactobacillus*, 1.5 g *Bifidobacterium bifidum*, 3 × 107 CFU *Bifidobacterium longum, Lactobacillus acidophilus* + *Enterococcus faecalis*, 18 × 107 CFU *Clostridium butyricum CGMCC0313.1*, 1.5 × 10 CFU *Bifidobacterium, B. infantis, L. acidophilus, Enterococcus faecalis, Bacillus cereus*, 0.84-1.68 g *Bifid.* Duration: 0-7 d before surgery to 0-28 d post-surgery *(Placebo or standard care)*	Time to flatus, time to defecation, weight loss	NR
Inception to June 2023				
Zeng et al^89^ SR	N = 8222 (56 RCTs, 3 RCTs on AEs n = 618) P = Patients with cancer I = Dietary supplements C = NR O = NR	Interventions: vitamin B3, vitamin D3, Zinc Duration: 18 wk to 6 y *(No treatment or placebo)*	NA	AEs to nutritional supplement interventions
Inception to Oct 2018				

Abbreviations: AAs, amino acids; AEs, adverse events; AIS, Athens Insomnia Scale; ALA, alpha lipoic acid; Arg, arginine; BCAA, Branch chained amino acids; BID, twice daily; BMI, body mass index; CFS, chronic fatigue scale; CFUs, colony-forming units; CI, confidence intervals; CINV, chemotherapy-induced nausea and vomiting; CIPN, chemotherapy-induced peripheral neuropathy; CoQ10, coenzyme Q10; CNS, central nervous system; CRF, cancer-related fatigue; CT, chemotherapy; CTCAE, NCI Common Terminology Criteria for Adverse Events; d, day(s); DHA, docosahexaenoic acid; EORTC, European Organisation for Research and Treatment of Cancer; EPA, eicosapentaenoic acid; FACIT, Functional Assessment of chronic illness; FACT, Functional Assessment of Cancer Therapy; FAs, fatty acids; FLIE, Functional Living Index Emesis; FOS, fructooligosaccharides; g, grams; GERD, gastroesophageal reflux disease; GI, gastrointestinal; GIQLI, Gastrointestinal Quality of Life Index; Gln, glutamine; GOS, galactooligosaccharides; HA, hyaluronic acid; HFS, hand and foot syndrome; HMB, beta-hydroxy b-methylbutyrate; HRQOL, health-related QoL; ISI, Insomnia severity index; IU, international units; KSS, Karolinska Sleepiness Scale; LOS, length of stay; MA, meta-analysis, MD, mean difference, MDASI, MD Anderson Symptom Inventory; MFI-20, Multidimensional Fatigue Inventory; mg, milligrammes; MOS, Medical outcomes study sleep survey; MPQ, McGill Pain Questionnaire; NA, not applicable; NAC, n-acetylcysteine; NCI, National Cancer Institute; nMA, network meta-analysis; NR, not reported, NS, not significant; NTX, neurotoxicity; OM, oral mucositis; OMAS, oral mucositis assessment scale; OIPN, oxaliplatin-induced peripheral neuropathy; OR, odds ratio; PAN, pancreatic cancer; PHGG, partially hydrolysed guar gum; PIPN, paclitaxel-induced peripheral neuropathy; PNP, peripheral neuropathy pain scale; PNQ, Patient Neurotoxicity Questionnaire; PSQI, Pittsburgh Sleep Quality Index; QID, four times daily; QLQ, QoL questionnaire; QoL, quality of life; RCT, randomised controlled trial; RD, radiodermatitis; RDSS, Radiation Dermatitis Severity Score; RINV, radiotherapy-induced nausea and vomiting; RR, risk ratio; RT, radiotherapy; RTOG, Radiation Therapy Oncology Group; SF-36, 36-item Short Form Health Survey; SOMA, subjective objective medical management and analytic evaluation of injury; SMD, standard mean difference; SR, systematic review; TID, three times daily; UR, umbrella review; wk, week(s); VAS, Visual Analogue Scale; WHO, World Health Organization grading system.

Amino acids or amino acid derivatives (eg, N-acetylcysteine [NAC], L-carnitine) were the most commonly investigated nutritional supplement (13 reviews),^40,42,47,55,57,66,68,70-73,76,84^ followed by pre/probiotics (11 reviews)^48,50,52,54,59,65,67,79,81,82,85^, vitamin E (12 Reviews)^40,42,44,45,57,58,63,69,76,80,83,84^, fatty acids (7 reviews),^43,47,51,64,70,72,89^ antioxidants (eg, coenzyme Q10 [CoQ10], alpha-lipoic acid [ALA]; 5 reviews)^40,42,72,76,78^, vitamin A/precursors (4 reviews),^45,76,83,87^ and zinc (3 reviews).^53,60,76^ Vitamin D^39,80,89^, vitamin C,^39,45,88^ selenium^45,61,80^, B vitamins^40,77^ and magnesium^90^ were also evaluated though less frequently. Other nutritional supplements included proteolytic enzymes,^62,75^ hyaluronic acid^74,83^, melatonin,^41,86^ curcumin^46,49^ and various phytochemical supplements (eg, fucoidan, adlay bran).^14,42,56,76^ Table 2 provides a detailed overview of all nutritional supplements evaluated.

### Quality Assessment

The methodological quality of the 51 systematic reviews and meta-analyses included for review was assessed (the umbrella review Khosroshahi et al^57^ was not applicable). The overall quality was generally poor, with 69% (35/51) being assessed as having critically low methodological quality due to the presence of more than one critical flaw. Of the remaining reviews, 29% (15/51) were assessed as low quality, while only one review (2%, 1/51) was assessed as having high methodological quality (Table 3). This review was by Croisier et al^48^ on dietary fibre/prebiotic supplementation for gynaecological cancers.^48^ Common critical flaws included a lack of justification for excluding individual studies (item 7), a partially inadequate literature search strategy (item 4), absence of prospective protocol registration (item 2) and inadequate discussion of risk of bias with review findings (item 13).

**Table 3.** AMSTAR-2 Assessment of Included Studies.

Author (reference)	Critical flaws	Outcome	Author (reference)	Critical flaws	Outcome
Allenby et al^45^	2, 7, 13	Critically low	Lin et al^42^	7, 13	Critically low
Amitay et al^81^	7, 13	Critically low	Liu et al^43^	2, 7	Critically low
An et al^85^	13	Low	Liu et al^90^	7	Low
Bagherniya et al^46^	2, 7, 9, 13	Critically low	Liu et al^60^	2, 7, 13	Critically low
Barnhart et al^80^	2, 7, 13	Critically low	Loprinzi et al^40^	2, 4, 7	Critically low
Behroozian et al^39^	2, 7	Critically low	Mahdavi et al^54^	2, 7	Critically low
Belloni et al^79^	7, 11, 15	Critically low	Momenzadeh et al^71^	7, 13, 15	Critically low
Chang et al^73^	7, 15	Critically low	Moustafa et al^84^	2, 7	Critically low
Chen et al^69^	2, 7, 13	Critically low	Nogueira et al^44^	7, 11, 15	Critically low
Cintoni et al^47^	7, 13	Critically low	Pandy et al^77^	2, 7, 13	Critically low
Cogo et al^82^	7	Low	Peng et al^72^	7, 13	Critically low
Crichton et al^68^	7, 15	Critically low	Prado et al^55^	7	Low
Croisier et al^48^	—	High	Raza et al^61^	7, 15	Critically low
da Anunciacao^49^	7	Low	Retzlaff et al^63^	2	Low
Deleemans et al^50^	2, 7, 13	Critically low	Retzlaff et al^87^	2	Low
Delmicon et al^51^	7	Low	Robijns et al^75^	2, 7, 15	Critically low
Dikeocha et al^52^	7	Low	Seo et al^86^	2, 7, 13, 15	Critically low
E Vasconcelos et al^76^	15	Low	Thu et al^65^	7, 11, 15	Critically low
Fan et al^41^	7	Low	Tsai et al^78^	13	Low
Feng et al^59^	7	Low	Umlauff et al^66^	7, 13	Critically low
Gremmler et al^62^	2	Low	vanGorkom^88^	2, 7	Critically low
Hoppe et al^53^	2	Low	Wierzbicka et al^83^	2, 7, 13	Critically low
Lam et al^64^	7, 15	Critically low	Wang et al^58^	2, 7, 13, 15	Critically low
Lee et al^74^	7, 15	Critically low	Wu et al^56^	2, 7, 13	Critically low
Leen et al^70^	2, 7, 13, 15	Critically low	Ye et al^67^	7, 15	Critically low
			Zeng et al^89^	2, 7	Critically low

### Efficacy

#### Body Weight

Ten reviews investigated nutritional supplement effects on body weight, including five meta-analyses^43,64-67^ and five systematic reviews.^47,49,51,53,55^ Table 4 summarises all included reviews that reported on nutritional supplement intervention and body weight. All reviews were assessed as either having low or critically low methodological quality.

**Table 4.** Nutritional Supplements for Nutrition-Related Side Effects.

Author (study design)	Intervention	Control	Outcomes (measurement tool/scale)	Certainty of evidence (GRADE)
Type of cancer				
Body weight/BMI				
Amino acids & derivatives				
Cintoni et al^47^ (SR) Pancreatic cancer	L-carnitine oral liquid formulation of L-carnitine Dose: NR Duration: 12 wk during CT	Placebo	Body weight ↑ BMI after 12 wk, *P* < .018 (1 RCT, n = 72)	NA
Prado et al^55^ (SR) Solid and head and neck cancers	HMB/Arg/Gln Intervention/dose: HMB/Arg/Gln (Ca-HMB 1.5 g, L-arginine 7 g, and L-glutamine 7 g) BID Duration: 8-24 wk during CT; 3 d pre surgery to 7 d post-surgery	Isonitrogenous isocaloric placebo	Weight loss (Body weight or BMI): Beneficial effect on increasing body weight (2 RCTs, n = 229, solid cancers) No effect on increasing body weight post-surgery NS (1 RCT, n = 60, head and neck cancer)	NA
Umlauff et al^66^ (MA) Prostate cancer	Protein powder Dose: 25 g whey protein containing 2.4 g leucine powder in water + 1200 mg calcium carbonate and 1000 IU vit D/d; 25 g whey protein isolate (EnergyFirst Manhattan Beach, CA) containing 4 g leucine BID; 20 g soy protein powder (Revival, Physicians Pharmaceuticals) containing 160 mg isoflavones (64 mg genistein, 63 mg daidzein, 34 mg glycitein)/d Duration: 12-52 wk	Usual care; home based flexibility programme; placebo with 20 g/d whole milk protein powder with similar nutrient content without isoflavones	BMI: ↔ in BMI with 25 g whey protein containing 2.4 g leucine + 1200 mg calcium carbonate and 1000 IU vit D /day (ES: 0.00, 95% CI: −0.47 to 0.47; 1 RCT, n = 60) ↔ in BMI for intervention with 25 g whey protein isolate BID or 20 g soy protein with isoflavones versus controls. 2 RCTs did not report post-intervention measures (2 RCTs, n = 70).	NA
Minerals & Vitamins				
Hoppe et al^53^ (SR) Colorectal cancer	Zinc Dose: NR Duration: approximately 16 wk	Placebo	BMI Difference between groups NS (1 RCT, n = 24)	NA
Fatty acids				
Delmicon et al^51^ (SR) Breast cancer	Omega-3 FAs Dose: 1.6 g EPA + 0.8 g DHA/d Duration: 6 mo	Placebo (Sunflower oil)	Body weight, maintain body weight during CT ↔ NS (1 RCT, n = 53)	NA
Lam et al^64^ (MA) Various cancers	Omega-3 FAs Dose: 360 mg-2.20 g EPA + 240 mg-1.6 g DHA O-3 FA capsules Duration: 30 d-6 mo Any treatment	No intervention, placebo, isocaloric oral nutritional supplement	Body weight, increase or maintain MD = NS (10 RCTs, n = 317)	⊕ΟΟΟ* Very low (due to very serious risk of bias and inconsistency)
Liu et al^43^ (MA) Colorectal cancer	Omega-3 FAs Dose: 70-600 mg EPA QID Duration: 56-84 d Treatment with surgery and/or CT	NR	Body weight, goal prevent weight loss MD = NS (5 RCTs, N = 222) BMI MD = NS (4 RCTs, N = 209)	Body weight ⊕ΟΟΟ* Very low (due to very serious imprecision and serious risk of bias and inconsistency)
Phytochemicals				
da Anunciacao et al^49^ (SR) Colorectal & prostate cancer	Curcumin Daily dose: 1.08-3 g/d curcumin Duration: 10, 20, and 30 d, during RT, RT/CT or CT; up to 2 y during RT	Control, rice bran	Weight loss ↑ “Significant improvement in weight loss in colorectal patients” (1 RCT, n = 71) Prostate cancer: NR (1 RCT, n = 45)	NA
Probiotics, prebiotics, & synbiotics				
Thu et al^65^ (MA) Breast cancer	Probiotics Strain(s)/dose: Calorie-restricted diet + Mediterranean diet + (i) ProLBE (*Lactobacillus with Bifidobacterium, Enterococcus*) 3 capsules (0.84 g) BID (ii)ProLBS (*Lactobacillus with Bifidobacterium*, S*treptococcus*) 1 capsule (109 CFU) + 38.5 g FOS)/d; (iii)ProLB (*Lactobacillus with Bifidobacterium*) 1 sachet (4 × 109 CFU)/d Duration: 3-10 wk	Calorie-restricted diet + placebo, Mediterranean diet only, placebo	Weight loss MD NS (4 RCTs, n = 298) Subgroup analysis by probiotic type: ProLBS, MD NS (2 RCTs, n = 164) ProLBE vs. placebo, MD: −3.20,95% CI −5.97 to −0.43, *I*^2^ = 0%, *P* = .02 (1 RCT, n = 100) -↓ body weight with PRoLB + a Mediterranean diet vs Mediterranean diet only. MD 6.40, 95% CI 0.57-12.23, *P* = .03 (1 RCT, n = 34) Subgroup analysis by duration of intervention: NS BMI Overall and subgroup analysis of probiotic type: MD NS (5 RCTs, n = 374)	⊕OOO* Very low (due to very serious risk of bias and imprecision)
Ye et al^67^ (MA) Gastric Cancer	Probiotics Strain(s)/daily doses: 50 zmg *Saccharomyces cerevisiae* Hansen CBS 5926, 4-6 g *Bifidobacterium* + *Lactobacillus*, 1.5 g *Bifidobacterium bifidum*, 3 × 107 CFU *Bifidobacterium longum, Lactobacillus acidophilus* + *Enterococcus faecalis*, 18 × 107 CFU *Clostridium butyricum CGMCC0313.1*, 1.5 × 10 CFU *Bifidobacterium, B. infantis, L. acidophilus, Enterococcus faecalis, Bacillus cereus*, 0.84-1.68 g *Bifid.* Duration: 0-7 d prior to surgery + 0-28 d post-surgery	Placebo or standard care	Weight loss MD NS (2 RCTs, n = 136)	⊕OOO* Very low (due to very serious risk of bias and imprecision)
Chemotherapy-induced or radiotherapy-induced nausea and vomiting				
Probiotics, prebiotics & synbiotics				
Croisier et al^48^ (SR) Gynaecologic cancer	Pre/Probiotics Dose: 150 ml yoghurt with 6.5% lactulose + 2 × 109 L*actobacillus acidophilus*/d; hydrolysed rice bran 3 g TID Duration: 5-7 wk, starting 5-7 d pre-RT	Nil supplement, standard care, placebo	Nausea, vomiting, anorexia (Study-specific numerical tool): No significant difference between intervention and control (2 RCTs, n = 41)	NA
Deleemans et al^50^ (SR) Cervical cancer	Pre/pro/synbiotics Dose: Synbiotic biogel with 10 × 106 CFU *L. acidophilus* NCFM + 1 × 106 *Bifidobacterium lactis* Bi-07 + blue agave inulin TID Duration 7 wk during CT and RT	Placebo	CINV (frequency and intensity) ↓ vomiting frequency and intensity, *P* < .001 (1 RCT, n = 70)	NA
Other nutrient supplements				
Gremmler et al^62^ (SR) Pelvic cancer	Proteolytic enzymes Dose: 4 tablets WOBE MUGOS^®^ (E) TID Duration: Commencing 3 d prior to RT to end of RT	Placebo	Nausea and vomiting (frequency) NS for both nausea and vomiting (1 RCT, n = 56)	NA
Wu et al^56^ (SR) Colorectal cancer	Fucoidan (seaweed compound) Dose: 4 g BID Duration: 6 mo	Cellulose powder	Vomiting: NS (1 RCT, n = 54)	NA
Oral mucositis				
Amino acids & derivatives				
Amiri Khosroshahi et al^57^ (UR) Various cancers	Glutamine Dose: 8-30 g/d glutamine Duration: 9 d-5 wk Follow-up: 28-3 y during RT	Placebo, no treatment, usual care	OM: (NCI-CTC, WHO, RTOG, OMAS, NR) Incidence grades 3-4: RR 0.49, 95% CI 0.32-0.73, *P* = .001 (16 RCTs, n = 1199)	⊕⊕ΟΟ+ Low
Minerals & Vitamins				
Allenby et al^45^ (SR) Head & neck cancer	β-carotene Dose: NR Duration: during RT to 3 y post RT	Placebo	OM (RTOG): “Beta-carotene reduces mucositis” (1 RCT, n = 535)	NA
Amiri Khosroshahi et al^57^ (UR) Various cancers	Vitamin E Dose: 800 mg/d Duration: 5 d Follow-up: 2 y	Placebo	OM (WHO, RTOG) incidence (any grade): RR = NS (2 RCTs, n = 72)	⊕⊕ΟΟ+ Low
Hoppe et al^53^ (SR) Head and neck cancer	Zinc Dose: NR Duration: approximately 16 wk during RT	Placebo	OM (NR) Time of onset ↓ control group Grade 2, *P* = .017; Grade 3, *P* = .0003; reduced severity in intervention, *P* = .003; After RT, similar improvements in both groups (1 RCT, n = 97) Time of onset ↑ intervention, *P* < .001 and duration ↓ 3.12 wk vs 5.14 wk, *P* = .001 for oral cancer (1 RCT, n = 83)	NA
Liu et al et al.^60^ (MA) Various cancer types	Zinc Form: Oral zinc sulphate capsules, zinc chloride mouthwash, oral polapre-zinc rinse Dose: 30- 220 mg, BID to QID Duration: 28 d to 20 wk or 30-35 treatment sessions	Placebo	OM incidence (all scales) RR 0.67, 95% CI 0.47-0.95, *P* = .03; *I*^2^ = 84% (12 RCTs, n = 783) OM incidence (WHO) RR 0.49, 95% CI: 0.19-1.26, *P* = .14; *I*^2^ = 84% (5 RCTs, n = 392) Subgroup analysis of OM incidence by cancer type: Head and neck cancer: NS (6 RCTs, n = 339) Leukaemia: RR 0.37; 95% CI: 0.20-0.70, *P* = .002, *I*^2^ = 23% (2 RCTs, n = 212) Pharyngeal cancer: RR 0.31; 95% CI: 0.21-0.45, *P* < .0001, *I*^2^ = 0% (2 RCTs, n = 170) Stratified by scale or criteria used for OM incidence: RTOG: RR 0.77, 95% CI 0.36-1.64 (4 RCTs, n = 245); WHO: RR 0.49, 95% CI 0.19-1.26 (5 RCTs, n = 396); Other scales: RR 0.77, 95% CI 0.61-0.97 (3 RCTs, n = 146)	Zinc for OM incidence (WHO):⊕ΟΟΟ Very low * (due to very serious risk of bias and inconsistency)
Raza et al^61^ (MA) Head and neck cancer	Selenium Dose: 400 mcg selenium per day Duration: during RT	Placebo	Incidence of severe OM (NR): Grade 3 or 4 OM NS (1 RCT, n = 71)	
Retzlaff et al^63^ (SR) Haematologic cancer	Vitamin E Dose: 3 arm intervention: Arm A: topical vitamin E paste 1 g BID Arm B: 200 mg oral vitamin E BID Duration: 4 d prior to CT/RT up to 3 mo post CT and 3 y post RT	Placebo	Grade III/IV OM (WHO) Incidence of grade III/IV OM NS post cycle 1 and cycle 2 Cycle 3: Arm A: 26.3% Arm B: 33.3% Arm C: 31.8; *P* = .01 Cycle 4: Arm A: 26.3% Arm B: 43.7% Arm C: 31.8; *P* = .01 (1 RCT, n = 76)	NA
Wang et al^58^ (nMA)	Vitamin E Dose: vitamin E mouthwash Duration: 3 wk	Placebo	Grade III/IV OM 2 wk post RT WMD: −0.74; 95% CI: −0.94 to −0.54 (p NR) 3 wk post RT WMD: −0.94; 95% CI: −1.03 to −0.85 (p NR) (1 RCT, n = 59, head and neck cancer) Rank probability indicated most effective OM mouthwashes were GM-CSF (54%), vitamin E (24%), natural drugs (43%) 10 Mouthwash types compared: sodium bicarbonate, phenylbutyrate, GM-CSF, phenytoin gabapentin, chlorhexidine, povidone iodine, natural drugs (Achillea extract, Althea root extract, Plantago major extract, curcumin, royal jelly), placebo (13 RCTs in nMA, n = 570)	
Probiotics, prebiotics & synbiotics				
Feng et al^59^ (MA) Various cancers	Probiotics Strain(s)/dose: (i) 1 capsule BID 109 CFU *Lactobacillus plantarum* MH-301, *Bifidobacterium animalis subsp. Lactis* LPL-RH, *L. rhamnosus* LGG-18, *L. acidophilus; (ii)* 3 capsules BID *B. longum, L. lactis, Enterococcus faecium*; (iii) 3 g 108 CFU/day Yakult (*B. breve* Yakult, *Lactobacillus casei* Shirota, GOS*)*; (iv) 2 × 10^9^ CFU/lozenge, 6 lozenges/day *L. brevis* CD 2; (v) 1-2 capsule(s)10 × 10^9^ CFU BID *L. rhamnosus* GG; (vi) *Lactococcus lacti* oral rinse Duration: 2 d prior to CT to end of CT for 24 wk	Placebo, usual care	OM Incidence (CTCAE/RTOG): ↓ RR = 0.84, 95% CI 0.78-0.91, *P* < .00001, *I*^2^ = 28% (4 RCTs, n = 412) Severe OM (Grades 3 & 4): ↔ RR NS (7 RCTs, n = 647)	NA
Other nutrient supplements				
Fan et al^41^ (MA) Various cancers	Melatonin Dose: 10-20 mg/d or 3% melatonin oral gel Duration: 14 d to 7 wk	Placebo, no treatment	Stomatitis incidence (WHO-G, NR): RR NS (7 RCTs, n = 832) All cancers RR 0.47, 95% CI 0.26-0.88, *P* = .02, *I*^2^ = 66% (4 RCTS, n = 674) All cancers except head and neck cancer Reducing severe stomatitis (WHO-G) RR NS (3 RCTs, n = 154)	Reducing severe stomatitis ⊕ΟΟΟ* Very low (due to very serious risk of bias and inconsistency)
Gremmler et al^62^ (SR) Various cancers	Proteolytic enzymes Dose: 3-4 tablets WOBE MUGOS^®^ (E) TID- QID, 4 tablets WOBE MUGOS^®^ TID; 5-15WOBE MUGOS^®^ tablets/day; 5 Wobenzym^®^ tablets TID, 10 WOBE MUGOS^®^ dragees TID Duration: Commencing 2-6 d prior to RT, during RT for 45-68 d; day 2-7 of every CT cycle	Placebo	OM (RTOG/EORTC): ↓ lower average values, *P* = .041), ↔ maximum values NS; oropharynx carcinoma (1 RCT, n = 69) OM grade (RTOG/EORTC): ↓ 1.32 ± 0.64 intervention vs. 2.24 ± 0.60 control, *P* < .0001 in head and neck cancer (1 RCT, n = 100) OM (NR) average values: ↓1.9 ± 0.56 intervention vs. 2.5 ± 0.59 control, *P* = .014 in head and neck cancer (1 RCT, n = 39)	NA
Wu et al^56^ (SR) Colorectal cancer	Fucoidan (seaweed compound) Dose: 4 g BID Duration: 6 mo	Cellulose powder	Incidence of OM (NR) NS (*P* = .25; 1 RCT, n = 54)	NA
Postoperative ileus				
Probiotics, prebiotics & synbiotics				
Amitay et al^81^ (MA) Colorectal cancer	Probiotics Strain(s)/dose: Lactobacillus acidophilus + Bifidobacterium longum + Enterococcus faecalis; B. longum + L. acidophilus + L.lactis + L.casei + B.bifidum + B.infantis; L. acidophilus + L. plantarum + L. lactis + Saccharomyces boulardii; L. plantarum + L. acidophilus + B. longum Dose: NR Duration: 7-16 d, 7 d prior to until 14 d post-surgery Follow-up: NR or 30 d	Placebo	Postoperative ileus (time to first stool) Days until return to normal gut function: MD = −0.66, 95% CI −0.93 to −0.39, *P* < .001 (4 RCTs, n = 398)	⊕ΟΟΟ* Very low (due to very serious imprecision and serious risk of bias and indirectness)
Cogo et al^82^ (SR) Colorectal, pancreatic cancers	Probiotics Strain(s)/dose: *Saccharomyces boulardii* 50 × 106 *CFUs QID; Bifidobacterium bifidum* + *B. infantis* + *Lactobacillus acidophilus* + *L. casei* + *L. salivarius* + *L. lactis* 10 × 109*BID; L. casei* Shirota 40 × 10^9^ + *B. breve* Yakult *10* × 10^9^ *+* *2*.5 g *GOS* QID; *L. plantarum 299 v 100 × 10*9 CFUs in oatmeal-based drink *100* ml *QID*, *L. casei* Shirota *1 g* + *B. breve* Yakult *1* g + GOS *15* g QID; *B. animalis subsp. L. lactis* HY8002 100 × 106 *CFU)* *+* *L. casei HY2782* 50 × 106 CFU + *L. plantarum HY7712* 50 × 106 *CFU* + *xylooligosaccharides 350* *mg + FOS 36* *mg BID; B. longum* + *L. acidophilus* + *Enterococcus faecalis* 63 × 106 *CFUs TID* Duration: 3-28 d	Standard care, no treatment, placebo	Postoperative ileus (intestinal obstruction) 4/7 RCTs (57%) favoured intervention 1/7 RCTs (14%) favoured control 2/7 RCTs (29%) showed no effect	NA
Ye et al^67^ (MA) Gastric cancer	Probiotics Strain(s)/daily doses: 500 mg *Saccharomyces cerevisiae* Hansen CBS 5926, 4-6 g *Bifidobacterium* + *Lactobacillus*, 1.5 g *Bifidobacterium bifidum*, 3 × 107 CFU *Bifidobacterium longum, Lactobacillus acidophilus* + *Enterococcus faecalis*, 18 × 10^7^ CFU *Clostridium butyricum CGMCC0313.1*, 1.5 × 10 CFU *Bifidobacterium, B. infantis, L. acidophilus, Enterococcus faecalis, Bacillus cereus*, 0.84-1.68 g *Bifid.* Duration: 0-7 d before surgery + 0-28 d post-surgery	Placebo or standard care	Time to flatus MD −11.27 95% CI −16.83 to −5.70 (7 RCTs, n = 693) Time to defecation MD −11.27 95% CI −16.83 to −5.70 (4 RCTs, n = 413)	Time to flatus: ⊕ΟΟΟ* Very low (due to very serious risk of bias and inconsistency and serious indirectness and imprecision)
Xerostomia/salivary gland function/dysphagia				
Minerals & Vitamins				
Allenby et al^45^ (SR) Head & neck cancer	Vitamins C + E Dose: NR Duration: During Cisplatin treatment Follow-up: 6 mo post RT	Placebo	↓ Xerostomia: (as measured by a patient reported xerostomia) Questionnaire (mean reduction in score of 2.7) and an observer-rated xerostomia score (mean reduction in score of 1.3; 1 RCT, n = 52)	NA
Barnhart et al^80^ (SR) Thyroid cancer	Selenium Dose: 300 mcg/d Duration:10 d, from 3 d before to 6 d after radioactive iodine	Placebo	Salivary gland function: ↓ serum amylase, *P* = .009 ↓ in maximum secretion % of two glands, *P* = .039 ↔ improvements in questionnaire symptom scores NS Placebo group showed ↑ salivary gland deterioration vs. selenium (*P* < .05; 1 RCT, n = 16)	NA
	Vitamin E Duration: 100-300 mg/d Dose: During RT	Placebo	Salivary gland function: Patients experienced increased function (treatment vs control) ↓ uptake fraction of the parotid glands (*P* < .001) ↑ excretion fraction of submandibular glands and some parotid glands with different doses of Vitamin E (*P* < .05) ↑ uptake index of submandibular and parotid glands (*P* < .05; 1 RCT, n = 82)	NA
Retzlaff et al^63^ (SR) Thyroid cancer	Vitamin E Dose: α-tocopherol 800 IU/d Duration: 5 wk from 4 d prior to CT/RT to 3 mo post CT and 3 y post RT	Placebo	Salivary gland function: ↑ Protective effect from RT ↑ first-minute uptake ratio in the left parotid gland (*P* = .04) ↑ excretion fraction in the right parotid gland (*P* = .04) (1 RCT, n = 36)	NA
Fatty Acids				
Delmicon et al^51^ (SR) Breast cancer	Omega-3 FAs (Fish oil capsule) Dose: 1.6 g EP*A* + 0.8 g DHA/d Duration: 6 mo	Placebo (Sunflower oil)	Xerostomia (Edmonton Scale): ↑ Acute reduction and reduced xerostomia 6 mo post RT (*P* = .032; 1 RCT, n = 53)	NA
Other nutrient supplements				
Gremmler et al^62^ (SR) Head and neck cancer	Proteolytic enzymes Dose: 3 tablets WOBE MUGOS^®^ (E) TID Duration: Commencing 2-6 d prior to RT, to completion RT for 45 to 68 d	Placebo	Dysphagia (EORTC/RTOG): ↓ grade: 1.33 ± 0.63 intervention vs. 2.24 ± 0.60 control, *P* < .001 (1 RCT, n = 100)	NA

Key: ↓ denotes significant decrease; ↑ denotes significant increase; ↔ non-significant/no difference.

Abbreviations: Arg, arginine; BCAA, Branch chained amino acids; BID, twice daily; BMI, body mass index; CFUs, colony-forming units; CINV, chemotherapy-induced nausea and vomiting; CT, chemotherapy; CTCAE, NCI Common Terminology Criteria for Adverse Events; d, day(s); DHA, docosahexaenoic acid; EORTC, European Organisation for Research and Treatment of Cancer; EPA, eicosapentaenoic acid; FAs, fatty acids; g, grams; GM-CSF, granulocyte-macrophage colony-stimulating factor; HMB, beta-hydroxy b-methylbutyrate; IU, international units; mcg, micrograms; MD, mean difference; mo, month(s); NA, not available; NCI, National Cancer Institute, Common Terminology Criteria; nMA, network meta-analysis; NS, not significant; OM, oral mucositis; OMAS, Oral Mucositis Assessment Scale; QID, four times per day; RCT, randomised controlled trial; RINV, radiotherapy-induced nausea and vomiting; RR, risk ratio; RT, radiotherapy; RTOG, Radiation Therapy Oncology Group; TID, three times per day; UR, umbrella review; WHO, World Health Organization grading system.

+ Original authors.

* Current authors.

##### Weight Loss Prevention

There is inconsistent evidence for perioperative supplementation with b-hydroxy b-methylbutyrate, arginine and glutamine (HMB/Arg/Gln).^55^ In individuals with various solid cancers, oral HMB/Arg/Gln supplementation may be beneficial for increasing body weight compared with isonitrogenous, isocaloric controls (2 RCTs, n = 229).^55^ However, oral HMB/Arg/Gln did not have an effect on body weight in individuals with head and neck cancer with the same control (1 RCT, n = 60).^55^ Interpretation of these findings of HMB/Arg/Gln is further limited by the absence of the effect estimates and confidence intervals from these RCTs within the systematic review.^55^ In a single RCT (n = 72), supplementation with L-carnitine during chemotherapy was effective for preventing weight loss in individuals with pancreatic cancers compared with placebo.^47^ This evidence is also limited by sample size and lack of reporting on intervention dosages, effect estimates and confidence intervals.

There was also very low certainty evidence from 2 meta-analyses^43,64^ and one systematic review^51^ that omega 3 fatty acids (O3-FAs) supplementation is not effective for preventing weight loss during treatment in individuals with colorectal cancers (5 RCTs, n = 222),^43^ breast cancer (1 RCT, n = 53) and other various cancer types (10 RCTs, n = 317).^64^

Oral curcumin supplementation was also reported to demonstrate “significant positive effects for preventing weight loss” in colorectal cancer survivors.^49^ However, these results were narratively synthesised, and effect estimates were not provided to support these findings.

##### Weight Loss Promotion

There was low certainty of evidence that probiotic supplementation is not effective for aiding weight loss in breast cancer survivors, based on pooled data from various *Lactobacillus* with *Bifidobacterium* formulations with and without prebiotics (fructooligosaccharides [FOS]; 4 RCTs, n = 298).^65^ Following subgroup analysis by probiotic type, *Lactobacillus* with *Bifidobacterium* and *Enterococcus* (2 RCTs, n = 164) or *Lactobacillus* with *Bifidobacterium* plus a Mediterranean diet (1 RCT, n = 34; both interventions without FOS) demonstrated efficacy for reducing body weight when compared with placebo or Mediterranean diet only.^65^

Supplementation with zinc^53^ and protein powders, including both whey protein isolate and soy protein with isoflavones,^66^ was not effective for reducing body weight in individuals with colorectal and prostate cancers compared with controls.

#### Cardiotoxicity

Our review identified 1 systematic review^84^ (with critically low methodological quality) evaluating the impact of nutritional supplementation to ameliorate cardiotoxicity outcomes in cancer survivorship. Moustafa et al^84^ investigated the prophylactic efficacy of vitamin E and levocarnitine on doxorubicin-induced cardiotoxicity. L-carnitine was reported to be “cardioprotective” compared with silymarin (1 RCT, n = 41) and vitamin E was “cardioprotective” in 3 of the 5 RCTs (n = 102) and “not cardioprotective” in 2 of the RCTs (n = 85) compared with placebo or no intervention. The efficacy of these interventions is significantly limited by the absence of validated cardiotoxic outcome measures and a lack of quantitative data regarding effect estimates and measures of variability. The authors also concluded the RCT data was ambiguous, controversial and of low quality.^84^ The available details extracted from this review can be found in Table 5.

**Table 5.** Nutritional Supplements for Reproductive and Cardiometabolic Side Effects.

Lead author (study design), cancer type	Intervention	Control	Outcomes (measurement tool/scale)	Certainty of evidence (GRADE)
Cardiotoxicity				
Amino acids & derivatives				
Moustafa et al^84^ (SR) Breast cancer and non-Hodgkin lymphoma	L-carnitine Dose: 3 g before CT + 1 g every d (per oral) Duration: During CT, follow-up at 6 mo	Silymarin	“Cardioprotective” (1 RCT, n = 41)	NA
Minerals & Vitamins				
Moustafa et al^84^ (SR) Breast cancer and lymphoma	Vitamin E Dose: 800-1800 IU/d, 400-600 mg/d (per oral) Duration: 1.4-18 mo	No intervention, placebo, NR	3/5 RCTs “Cardioprotective” (3 RCTs, n = 102) 2/5 RCTs “Not cardioprotective” (2 RCTs, n = 85)	NA
Genitourinary complications				
Minerals & Vitamins				
Barnhart et al^80^ (SR) Breast cancer	Vitamin D or Vitamin E Dose: NR Duration: NR	Placebo	Genitourinary atrophy: ↑ Improvement in symptoms (self-reported; *P* < 0.0001; 1 RCT, n = 96)	NA
Other nutrient supplements				
Wierzbicka et al^83^ (SR) Cervical cancer	Hyaluronic acid + vitamin A + vitamin E Dose: 5 mg HA, 1 mg vitamin A, 1 mg vitamin E, topically Duration: 5-16 wk	NR	Genitourinary side effects ↓ vaginal dryness I: 0.63 ± 0.33, C: 2.43 ± 0.50; *P* < .001 ↓ dyspareunia I: 2.46 ± 0.50, C: 0.74 ± 0.43; *P* < .001 ↓ mucosal Inflammation I: 0.77 ± 0.25, C: 2.47 ± 0.50; *P* < .001 (1 RCT, n = 177) ↓ dyspareunia I: 23%, C: 69%; *P* < .05 ↓ mucosal inflammation I: 23%, C: 75%, *P* < .05 ↓ vulvar fibrosis I: 18%, C: 56%; *P* < .05 (1 RCT, n = 45)	NA
Hot flushes				
Minerals & Vitamins				
Liu et al^90^ (NMA) Breast cancer	Magnesium Dose: Magnesium oxide (i) 800 mg, (ii) 1200 mg/d Duration: 8 wk	Placebo	Hot flash frequency and score (Hot flash diary) NS (1 RCT, n = 358)	NA
Retzlaff et al^63^ (SR) Breast cancer	Vitamin E Dose: vitamin E succinate 800 IU/d (≙536, 91 mg/d) Duration: 4 wk	Placebo	Hot flash frequency and severity NS (1 RCT, n = 125)	NA
Lymphoedema				
Probiotics, prebiotics & synbiotics				
Thu et al^65^ (MA) Breast cancer	Probiotics Dose: Calorie-restricted diet +; Mediterranean diet +, ProLBS (*Lactobacillus + Bifidobacterium + Streptococcus* 1 × 109 CFU) + 38.5 g FOS/d; ProLBS (*Lactobacillus + Bifidobacterium + Streptococcus* 1 × 10^9^ CFU) Duration: 3-10 wk	Calorie-restricted diet + placebo, Mediterranean diet only, placebo	Oedema volume: NS (2 RCTs, n = 223)	⊕ΟΟΟ* Very Low (due to serious risk of bias and very serious imprecision)
Other nutrient supplements				
Gremmler et al^62^ (SR) Breast cancer	Enzymes Dose: 5 Wobe-Mugos^®^ tablets (containing papain, trypsin, chymotrypsin) TID Duration: 6.5 wk during combined decongestive therapy	Placebo	Lymphoedema Arm volume NS Fibrosis (skin fold thickness) NS ↓ skin tension baseline to visit 4: intervention (2.0, 0.4) vs control (1.8, 0.5), p NR (1 RCT, n = 88)	NA

Key: ↓ denotes significant decrease; ↑ denotes significant increase; ↔ non-significant/no difference.

Abbreviations: CFU, colony forming units; CT, chemotherapy; GRADE, Grading of Recommendations, Assessment, Development and Evaluation; HA, Hyaluronic acid; IU, International Units; MA, meta-analysis; NA, not applicable; NR, not reported, NS, not significant; SR, systematic review; TID, three times per day.

+ Original authors.

* n of control group not reported for 2 RCTs. Current authors.

#### Chemo/Radiotherapy Induced Nausea/Vomiting (CINV/RINV)

Three systematic reviews investigated proteolytic enzymes,^62^ fuciodan,^56^ and pre/probiotics^50^ for CINV with low or critically low methodological quality. One systematic review evaluated the effect of pre/probiotics for RINV^48^ with high methodological quality. Among the evaluated interventions, there is limited evidence for the efficacy of nutritional supplementation for CINV/RINV.

The only intervention to demonstrate efficacy was a synbiotic supplement containing *L. acidophilus* NCFM and *Bifidobacterium lactis* with inulin.^50^ A single RCT (n = 70) reported a significant reduction in the frequency and intensity of CINV in individuals with cervical cancer undergoing chemo/radiotherapy.^50^ Pre/probiotic supplementation with lactulose and *L. acidophilus-*enriched yoghurt and hydrolysed rice bran, however, were not effective for RINV in individuals with gynaecological cancers undergoing radiotherapy (2 RCTs, n = 41).^48^ All other interventions showed no benefits over placebo/control (Table 4).

#### Cognitive Changes

Evidence of the effects of nutritional supplementation on cognition was sparse (Table 6). We identified one review by Cintoni et al^47^ reporting on intervention with L-carnitine for cognitive function in individuals with pancreatic cancer undergoing chemotherapy. Evidence from a single RCT (placebo-controlled, double-blinded) with 72 participants showed a significant improvement in cognitive function using validated measures (QLQ-C30, Pancreatic Cancer Module [PAN 26]).^47^ The review was assessed as having critically low methodological quality.

**Table 6.** Nutritional Supplements for Neurological and Cutaneous Side Effects.

Author, study design	Intervention	Control	Outcomes	Certainty of evidence (GRADE)
Chemotherapy-induced peripheral neuropathy				
Amino acids & derivatives				
Crichton et al^68^ (MA) Colorectal cancer	Glutamine Dose: 30 g/d divided doses (BID) for 7 d every 2 wk Duration: Commencing C1 CT for 6 cycles	Standard care	Incidence of CIPN (NR): ↓ reduced vs. standard care (1 RCT, n = 86)	⊕ΟΟΟ+ Very Low (due to the risk of bias found in “most” included studies and the number of participants < 100)
Leen et al^70^ (MA) Ovarian cancer and Breast cancer	NAC Dose: (i) 600 mg NAC BID (ii) 1200 mg NAC BID Duration: 12 wk	No treatment/usual care	Incidence of PIPN (FACT/Gynaecologic Oncology Group-NTX subscale): NS (1 RCT, n = 65)	NA
	Glutamate Dose: 500 mg TID; dosed 30 min prior to or 2 h post prandial Duration: during CT to 3 wk post	Placebo	Incidence of PIPN (NR): NS (1 RCT, n = 43)	NA
Loprinzi et al^40^ (SR) Various cancer types	Acetyl-L-Carnitine Dose: 1 g every 3 d Duration: during CT	Placebo	CIPN (FACT-NTX) ↔ CIPN overall score and median duration: NS (1 RCT, n = 150)	NA
Momenzadeh et al^71^ (MA) Breast cancer	Acetyl-L-Carnitine Dose: 3 g/d Duration: 56-168 d during CT (paclitaxel)	No treatment	PIPN (FACT-NTX): RR NS (3 RCTs, n = 858)	NA
Peng et al^72^ (MA) Colorectal cancer	L-carnosine Dose: 500-1000 mg/d Duration: Prior to and during CT for 3 mo	Non-active intervention	Severity of OIPN (CTCAE): ↓ severity Grade ≥ 2: RR 0.05, 95% CI 0.01-0.22, *I*^2^ = 0%, *P* < .0001 (2 RCTs, n = 121)	Incidence of OIPN: Grade ≥ 2: ⊕⊕ΟΟ+ Low (due to risk of bias (2 levels)
	Cysteine + theanine Dose: 700 mg cysteine + 280mg theanine/day Duration: during CT for 6 wk	Placebo, non-active intervention	OIPN (CTCAE, study specific questionnaire): “Reported as significant benefit “(1 RCT, n = 28)	Incidence of OIPN Grade ≥ 2: ⊕ΟΟΟ+ Very Low (due to risk of bias (2 levels), indirectness (1 level))
	Glutamine Dose: 30 g/day Duration: 1 d before and during CT (8 wk), 7d every 2 wk for 6 wk	Non-active intervention	Severity of OIPN (CTCAE): ↓ severity Grade 3 to 4: RR 0.37 95% CI 0.15-0.95, p NR (1 RCT, n = 86)	Incidence of OIPN Grade ≥ 2: ⊕⊕ΟΟ+ Low (due to high risk of bias (2 levels))
	NAC Dose: 1200 mg/d 1 h before CT Duration: Prior to and during CT, 8 to 12 wk	Placebo	Severity of OIPN (CTCAE): ↓ severity Grade ≥ 2: RR 0.26 95% CI 0.12-0.56, *I*^2^ = 0%, *P* = .0006 (2 RCTs, n = 46)	Incidence of OIPN Grade ≥ 2: ⊕⊕⊕Ο+ Moderate (due to risk of bias (1 level))
Fatty acids				
Loprinzi et al^40^ (SR) Various cancer types	Alpha-lipoic acid Dose: 600 mg TID Duration: 24 wk during CT	NR	CIPN (FACT-NTX) CIPN overall score and median duration: NS (1 RCT, n = 243)	NA
Lam et al^64^ (MA) Various cancers	O3-FAs Dose: 1.9, 0.192, and EPA + 1.04 g DHA omega-3 PUFA oral capsules/d Duration: 13-25 wk	Placebo	CIPN incidence (NR) ↓ incidence OR = 0.20, 95% CI 0.10-0.40, *I*^2^ = 0%, *P* < .00001 (3 RCTs, n = 158)	Incidence of CIPN ⊕ΟΟΟ* Very Low (due to very serious risk of bias and imprecision)
Vitamins				
Chen et al^69^ (MA) Various cancers	Vitamin E Dose: 300 to 600 mg vitamin E/d Duration: Prior to CT and at the completion of CT	NR	CIPN (PNP, CTCAE) ↓ Incidence of all-grade CIPN: RR 0.55, 95% CI: 0.36-0.85, *I*^2^ = 77.3%, *P* = .007 (8 RCTs, n = 307) Severity CIPN: NS (3 RCTs, n = 262)	Incidence of all-grade CIPN: ⊕ΟΟΟ* Very Low (due to serious risk of bias and very serious inconsistency)
Loprinzi et al^40^ (SR) Various cancer types	Vitamin B Dose: Vitamin B complex (50 mg thiamine, 20 mg riboflavin, 100 mg niacin, 163.5 mg pantothenic acid, 30 mg pyridoxine, 500 mg folate, 500 mg cyanocobalamin, 500 mg biotin, 100 mg choline, and 500 mg inositol) BID Duration: 24 wk during CT	Placebo	CIPN (PNQ) Incidence: NS ↓ Patient perceived sensory peripheral neuropathy at 36 wk, *P* = .021 (1 RCT, n = 71)	NA
Retzlaff et al^63^ (SR) Various cancers	Vitamin E Dose: 300 mg DL-alpha-tocopherol BID, Duration: During and up to 3 mo post CT	No treatment	CIPN (NR) ↓ CIPN incidence: RR = 2.51, 95% CI: 1.16-5.47) ↓ CIPN score (*P* = .023) Significant differences between groups in all three sensory nerves tested, ulnar nerve (*P* = .021), superficial peroneal nerve (*P* = .017), nervus suralis (*P* = .046; 1 RCT, n = 30) ↓ PIPN incidence (*P* = .026) ↓ PIPN score (*P* = .01) Significant differences between groups in all three sensory nerves tested, ulnar nerve (*P* = .014), superficial peroneal nerve (*P* = .003), nervus suralis (*P* = .008; 1 RCT, n = 32)	NA
Cognitive function				
Cintoni et al^47^ (SR) Pancreatic cancer	L-Carnitine Dose: L-carnitine liquid formula Duration: 12 wk	Placebo	Cognitive function (EORTC QLQ-PAN26) ↑ cognitive function after 6 wk, *P* < .034 (1 RCT, n = 72)	NA
Dermatitis				
Amino acids & derivatives				
Chang et al^73^ (MA) Breast, head and neck cancers	Glutamine Dose: 14-30 g glutamine/day or in combination as HMB/Arg/Gln (14, glutamine + 2.4, and HMB + 14 g arginine) Duration: From 6 wk before RT/CT to 2 wk after treatment completion. NR in 1 R/CT	placebo (glucose solution, dextrose, maltodextrin), no treatment	Incidence of radiodermatitis (RTOG/CTCAE): ↓ RR 0.90, 95% CI 0.81-1.0, *P* = .05, *I*^2^ = 7% (5 RCTs, n = 218) Severity (RTOG/CTCAE): ↓ incidence severity of Grade 2-4: RR 0.49, 95% CI 0.32-0.76, *P* = .001, *I*^2^ = 52% (5 RCTs, n = 218)	Incidence: ⊕ΟΟΟ+ Very Low (due to the risk of bias, heterogeneity, CI include harm and benefit) Grade 2-4: Moderate ⊕⊕⊕Ο+ (due to the risk of bias)
E Vasconcelos et al^76^ (MA) Head and neck cancer	HMB/Arg/Gln Dose: 24 g HMB/Arg/Gln (1.2 g HMB, 7 g Arg, 7 g Gln) BID Duration: During CT/RT until 1 wk after CT/RT	No treatment	Dermatitis (CTCAE): ↓ Grade 1 and Grade 2 (*P* < .03, *P* < .02) ↔ Grade 3 (*P* < .44) (1 RCT, n = 34)	NA
Minerals & vitamins				
Behroozian et al^39^ (SR) Head and neck cancer, Breast cancer	Vitamin C Dose: Ascorbic acid solution Duration: NR Treatment: RT	Placebo	Dermatitis (CNS Cancer Consortium): ↔ between groups NS (1 RCT, n = 65)	NA
	Vitamin D Dose: Daivonex ointment (calcipotriol 50 mcg/d) Duration: NR Treatment: RT	Aqua cream	Dermatitis (RTOG): ↔ between groups NS (1 RCT, n = 23)	NA
E Vasconcelos et al^76^ (MA) Head and neck cancer	Vitamin E + vitamin A Dose: (i) 400 IU α-tocopherol + 300 mg β-carotene/d; (ii) 400 IU α-tocopherol Duration: During RT and for 3 y after RT completion	Placebo	Dermatitis (RTOG): ↔ in severity of RD between all groups (1 RCT, n = 535)	NA
	Zinc Dose: 25 mg zinc TID Duration: “approximately 2 mo” Treatment: RT	Placebo	Dermatitis (RTOG): ↓ Grade 2, *P* = .014 ↓ Grade 3, *P* = .0092 ↓ severity of inflammation (*P* = .03) (1 RCT, n = 100)	NA
Nogueira et al^44^ (MA) Breast cancer	Vitamin E Dose: 1000 mg or 1200 IU Duration: 6 mo Treatment: RT	Placebo or no intervention	Fibrosis (LENT-SOMA scoring scale): SMD: NS (2 RCTs, n = 64)	⊕ΟΟΟ+ Very Low (both included studies had methodologic limitations and one included study showed imprecision of the results)
Pandy et al^77^ (MA) Various cancer types	Vitamin B6 (Pyridoxine) Dose: 60-200 mg/d Duration: NR Treatment: chemotherapy	Placebo, no treatment or Eppikajutsuto (Kampo medicine)	Incidence of HFS: OR NS (8 RCTs, n = 661)	⊕ΟΟΟ* Very Low (due to very serious risk of bias and imprecision)
Robijns et al^75^ (MA) Cervical, head and neck cancers	Enzymes Dose: 100 mg papain, 40 mg trypsin, and 40 mg chymotrypsin QID Duration: 3 d before RT to 5 d post RT	No treatment	Radiodermatitis (RTOG) ↓ Incidence of moist desquamation: RR 0.60, 95% CI 0.31-0.85, *P* = .009, *I*^2^ = 49% (2 RCTs, n = 219) ↓ Incidence of grade 2+: RR 0.42, 95% CI 0.30-0.58, *P* < .00001, *I*^2^ = 0% (2 RCTs, n = 219)	Moist desquamation: ⊕⊕ΟΟ+ Low (due to risk of bias and imprecision) Grade 2+: ⊕⊕ΟΟ+ Low (due to risk of bias and imprecision)
Phytochemicals				
E Vasconcelos et al^76^ (MA) Breast cancer	Anthocyanins Dose: 125 mg anthocyanins TID Duration: 1 wk prior to RT and during RT 2-5 wk Treatment: RT		Dermatitis (RTOG): ↔ incidence or severity of RD (1 RCT, n = 192)	NA
Other nutrient supplements				
E Vasconcelos et al^76^ (MA) Breast cancer	Adlay Bran Dose: 1 g Adlay bran extract capsules BID Duration: 5-6 wk Treatment: RT	Olive oil capsules	Dermatitis (RTOG): ↓ Grade ≥2 reaction developed in 45.2% vs 75.7%, OR 0.24, *P* = .002 (1 RCT, n = 110)	NA
Lee et al^74^ (MA) Breast cancer	HA + betaglucan Dose: topical NR Duration: 15 d before RT until 30 d after RT	Alginate	Dermatitis (RTOG) RR NS (1 RCT, n = 40)	NA
	HA + beta glucan Dose: topically applied BID Duration: 15 d before RT until 30 d after RT	Avene thermal water	Dermatitis (RTOG) RR NS (1 RCT, n = 40)	NA
	HA or HA + beta glucan Dose: topically applied TID, NR Duration: 0-15 d before RT, during RT, 0-90 d after RT	Grapevine extract or shea butter + grapevine extract + glycyrhetininic acid + telmesteine	Dermatitis (RTOG/NCI) RR NS (3 RCTs, n = 120)	NA
	HA + beta glucan Dose: topically applied TID Duration: 0-15 d before RT, during RT, 15-90 d after RT	Omega 369	Dermatitis (RTOG) RR 3.00, 95% CI 1.5-5.95, *P* = .002 (1 RCT, n = 40)	NA
	HA Dose: topically applied Duration: 0-15 d before RT, during RT, 15-90 d after RT	Petrolatum-based substance or placebo cream	Dermatitis (NCI) RR NS (2 RCTs, n = 218)	NA
	HA + beta glucan Dose: topically applied Duration: 0-15 d before RT, during RT, 30-90 d after RT	Phytosterols	Dermatitis (RTOG/NCI) RR = 3.11, 95% CI 1.82-5.29, *I*^2^ = 8%, *P* < .0001 (2 RCTs, n = 80)	NA
	HA + beta glucan Dose: topically applied Duration: During RT until 90 d after RT	Vitamin E	Dermatitis (RTOG) RR = 1.50, 95% CI 1.02-2.21, *P* = .04 (1 RCT, n = 40)	NA
Sleep				
Fan et al^41^ (MA) Various cancers	Melatonin Melatonin ± bright white light therapy Dose: 3-20 mg melatonin/d before bed Duration: 10 d to 1 y, or until death	Placebo, bright white therapy alone, zolpidem	Sleep quality (AIS, EORTC, ISI, KSS, MOS, PSQI): SMD NS (9 RCTs, n = 112)	⊕ΟΟΟ* Very Low (due to very serious risk of bias and inconsistency)

Key: ↓ denotes significant decrease; ↑ denotes significant increase; ↔ non-significant/no difference.

Abbreviations: AAs, amino acids; AIS, Athens Insomnia Scale; Arg, arginine; BID, twice per day; CIPN, chemotherapy-induced peripheral neuropathy; CT, chemotherapy; CTCAE, Common Terminology Criteria for Adverse Events; EORTC, European Organisation for Research and Treatment of Cancer; FACT, Functional Assessment of Cancer Therapy; Gln, glutamine; GRADE, Grading of Recommendations, Assessment, Development and Evaluation; HA, Hyaluronic acid; HFS, hand and foot syndrome; HMB, beta-hydroxy b-methylbutyrate; ISI, Insomnia severity index; KSS, Karolinska Sleepiness Scale; MA, meta-analysis; MOS, Medical outcomes study sleep survey; NA, not applicable; NAC, n-acetylcysteine; NCI, National Cancer Institute; NMA, network meta-analysis; NR, not reported; NS, not significant; O3-FA, Omega-3 fatty acids; OIPC, oxaliplatin-induced peripheral neuropathy; PIPN, paclitaxel-induced peripheral neuropathy; PNP, Peripheral Neuropathic Pain; PNQ, Patient Neurotoxicity Questionnaire; PSQI, Pittsburg Sleep Quality Index; RT, radiotherapy, RTOG, Radiation Therapy Oncology Group; SR, systematic review; TID, three times per day.

+ Original authors.

* Current authors.

#### Dermatitis

Seven reviews evaluated nutritional management for treatment-induced dermatitis (Table 6). There were 4 meta-analyses^44,73-75^, one clinical practice guideline^39^ and 1 systematic review on radiation-induced dermatitis (RD)^76^ and one meta-analysis on chemotherapy-induced skin reactions,^77^ with all reviews being assessed as having critically low or low methodological quality. There is limited evidence for the efficacy of nutritional supplements to improve dermatitis outcomes. Glutamine and proteolytic oral enzymes demonstrated some evidence of effectiveness for RD outcomes in the included reviews. There is limited evidence that zinc and adlay bran are effective for reducing RD severity and inconsistent evidence on the efficacy of topical hyaluronic acid (HA). There were no effective nutritional supplement interventions for chemotherapy-induced skin reactions.

The amino acids HMB/Arg/Gln may be effective for reducing the overall incidence of RD (RTOG/CTCAE) compared with placebo or no treatment (5 RCTs, n = 218) with very low certainty.^73^ There was moderate certainty that glutamine or HMB/Arg/Gln reduces the incidence of moderate-severe RD (RTOG/CTCAE; OR 0.49, CI 0.32-0.76, *I*^2^ = 52%, 5 RCTs, n = 218).^73^ In individuals with head and neck or cervical cancer, there is low certainty that oral enzymes (papain, trypsin, chymotrypsin) may reduce the incidence of moist desquamation and severity of RD compared with no treatment with pooled data from 2 RCTs (n = 219).^75^

A 2022 meta-analysis by Lee et al^74^ evaluated the efficacy of topical HA compared with active controls in individuals with breast cancer. When compared with phytosterols (2 RCTs, n = 80), vitamin E (1 RCTs, n = 40), and O3-FAs (1 RCT, n = 40), topical HA was more effective in reducing the incidence of RD, however a GRADE assessment was not possible due to the use of outcome measurement scales which were not included in our pre-defined criteria. Topical HA did not demonstrate superior efficacy when compared with simple emollients or grapevine extract.^74^

Oral supplementation with zinc and adlay bran extract capsules may also be effective interventions for reducing the severity of RD. There is limited evidence that adlay bran (1 RCT, n = 110) and zinc (1 RCT, n = 100) may be effective for reducing RD severity (RTOG) in breast and head and neck cancer compared with placebo and olive oil capsules.^76^

A number of nutritional supplement interventions were not effective for dermatitis. There was very low certainty that pyridoxine is not effective for reducing the incidence of dermatitis compared with placebo, no treatment and active controls (8 RCTs, n = 661).^77^ There is low and very low certainty that neither oral nor topical curcumin are effective for reducing moist desquamation incidence or RD severity in individuals with breast and head and neck cancers compared with placebo controls and simple emollients.^75^ Oral vitamin E is not effective for preventing fibrosis in individuals with breast cancer when compared with placebo or no treatment (2 RCTs, n = 64),^44^ with very low certainty. Intervention with vitamin E combined with vitamin A,^76^ anthocyanins,^76^ vitamin D^39^ and vitamin C^39^ also did not demonstrate efficacy.

#### Fatigue

Six reviews (2 meta-analyses^41,78^ and 4 systematic reviews^50,53,62,79^) reported on nutritional supplementation to manage cancer-related fatigue (CRF; Table 7). Nutritional supplements generally did not demonstrate efficacy for CRF. There is limited evidence from 1 systematic review that pre/probiotics may improve fatigue scores.^79^ There was no evidence from available meta-analyses demonstrating efficacy against control/placebo.^41,78^ All reviews were assessed as having low or critically low methodological quality.

**Table 7.** Nutritional Supplements for QoL and Fatigue.

Lead author (study design) Type of cancer	Intervention	Control	Outcomes (measurement tool or scale)	Certainty of evidence (GRADE)
Quality of life				
Amino acids & derivatives				
Cintoni et al^47^ (SR) Pancreatic cancer	L-carnitine Dose: NR L-carnitine liquid formula Duration: 12 wk during CT	Placebo	QoL (Global health status): ↑ global health status after 12 wk, *P* < .041 (1 RCT, n = 72)	NA
Prado et al^55^ (SR) Various cancers	HMB/Arg/Gln Dose: 2 × HMB 1.5 g, arginine7 g, L-glutamine 7 g/d Duration: 8 wk, 24 wk	Isonitrogenous placebo	QoL (NR): Mixed and NS effects (2 RCTs, n = 229)	
Minerals & Vitamins				
Allenby et al^45^ (SR) Cervical cancer	Mixed antioxidants (-carotene, vitamin C/ E, selenium) Dose: NR Duration: During CT	Placebo	QoL (EORT QLQ-C30): “*Antioxidants improved QoL in women with cervical cancer*” (1 RCT, n = 103)	NA
Hoppe et al^53^ (SR) Colorectal cancer	Zinc Dose: NR Duration: 16 wk during CT	Placebo	QoL (NR): ↔ between groups, NS (1 RCT, n = 24)	NA
Loprinzi et al^40^ (SR)	B vitamins Dose: 1 capsule BID B vitamin complex (50 mg thiamine, 20 mg riboflavin, 100 mg niacin, 163.5 mg pantothenic acid, 30 mg pyridoxine, 500 mcg folate, 500 mcg cyanocobalamin, 500 mcg biotin, 100 mg choline, and 500 mcg inositol) Duration: NR	Placebo	QoL (EORTC QOL): ↔ between groups, NS (2 RCT, n = 47)	
Nogueira et al^44^ (MA) Breast cancer	Vitamin E Dose: 1000 mg Duration: 6 mo from end of treatment	Placebo	QoL (EORTC QLQ-C30, BR23): Self-reported: NS, ↔ either group (1 RCT, n = 68)	NA
Fatty acids				
Delmicon et al^51^ (SR) Breast cancer	O3-FAs Fish oil capsule Dose: 2.6 g/d EPA + 1.4 g/d DHA Duration: 24 wk	Placebo (A mixture of fats and oils formulated to mirror the ratio of FAs typical of the American diet)	QoL (FACT-ES): ↓ placebo group, ↔ intervention group at 12 wk (1 RCT, n = 44)	NA
Liu et al^43^ (MA) Colorectal cancer	O3-FAs Dose: 192 mg to 6.4 g/d EPA Duration: NR	NR	QoL (NR): MD = NS (3 RCTs, n = 332)	TBC
Phytochemicals				
Bagherniya et al^46^ (SR) Prostate cancer	Curcumin Dose: 1440 mg/d curcumin Duration: 6 mo	Placebo	QoL (NR): NS (1 RCT, n = 82)	NA
da Anunciacao et al.^49^ (SR) Various cancers	Curcumin Dose: 180 mg/d Meriva curcumin Duration: 8 wk during CT	Control	QoL (University of Washington QoL): ↑ QoL However, the curcumin group had a significantly lower quality of life score at baseline compared to the placebo group, “which may have reflected a more compromised health status and the possibility of greater response to treatment in these patients” (1 RCT, n = 80)	NA
Wu et al^56^ (SR) Colorectal cancer	Fucoidan sourced from *Sargassum hemiphyllum* of low molecular weight powder Dose: 8 g/d Duration: 6 mo	Cellulose powder	QoL (EORTC QLQ-CR29): ↔ in QoL (1 RCT, n = 54)	NA
Probiotics, prebiotics & synbiotics				
Croisier et al^48^ (SR) Gynaecologic cancers	Prebiotic Dose: Prebiotic mixture (inulin 50%, FOS 50%) 6 g BID Duration: 5-7 wk starting 5-7 d before RT	Placebo	QoL (EORTC QLQ-C30): ↔ “no clinically or statistically significant improvement in these parameters was found” (1 RCT, n = 38)	NA
Deleemans et al^50^ (SR) Various cancers	Prebiotic/Probiotics Dose: (i) Bifilact: *L. acidophilus* LAC- 361 + *B. longum* BB-536 BID 1.3 × 109 CFU BID or 10 × 10^9^ CFU TID; (ii) Lacidofil: *L. rhamnosus* R0011, *L. acidophilus* R0052 2 × 10^9^ CFU BID; (iii) PHGG BID; (iv) fibre-enriched jelly “jevity FOS” with 10 g fibre,7 g FOS/litre; (v) *L. plantarum* CJLP243 (KCCM11045P), isolated from kimchi 10 × 10^9^/d Duration: 3-12 wk	Placebo, control (standard western diet)	QoL (EORTC-QLQ-C30, FACT-C, GIQLI) (i) Bifilact ↔ EORTC-QLQ-C30 vs. Control, (1 RCT, n = 229, mixed cancer types)(ii) Lacidofil ↑ FACT-C, *P* = .04 (1RCT, n = 60, colorectal cancer) (iii) PHGG ↔ vs. control (1 RCT, n = 30, mixed cancer types undergoing pelvic RT) (iv) jevity FOS: maintained GIQLI vs. ↓ GIQOLI in control, (*P* = .027; 1RCT, n = 32, head and neck cancer) (v) ↔ in EORTC-QLQ-C30 or QLQ-CR29 vs. control (1RCT, n = 36, rectal cancer)	NA
Dikeocha et al^52^ (SR) Colorectal cancer	Prebiotics/Probiotics Dose: (i) Alfasigma capsule 112.5 × 10^9^ CFU BID containing *B. infantis, L plantarum, L paracasei, B. longum, L. bulgaricus, L. acidophilus, B. breve, and Streptococcus thermophilus)* (ii) 1 sachet MCP preparation (30 × 10^9^ CFU + O3-FAs/d) (iii) FourLAB: *Pediococcus pentosaceus, Leuconostoc mesenteriodes, Lactobacillus paracasei, L. plantarum + β*-glucan, inulin, pectin, resistant starch 12 g/d Duration: 15 d-4 wk, postoperatively for ≤15 doses or until discharge	Placebo, no treatment	QoL (FACT-G7, EORTC-QLQ-C30, GIQLI) (i) FACT-G7: ↔ intervention (16.3 ± 5.1 pre, 17.1 ± 5.0 post, *P* = .327) vs. ↓ control (21.6 ± 3.9 pre, 18.0 ± 6.3 post, *P* = .019) vs control (1 RCT, n = 135) (ii) EORTC-QLQ-C30 ↑ intervention vs control, 68.70 ± 1.90 vs 51.60 ± 2.20, *P* < .001 (1 RCT, n = 140) (iii) GIQLI: ↑ 1 mo (77.0 ± 1.7 intervention, vs. 71.4 ± 1.7 control, *P* = 0.01), 3-months (77 ± 1.7 intervention vs. 72.5 ± 1.7 control, *P* = 0.03), 6-months (79.2 ± 1.8 intervention vs. 72.8 ± 1.9 control, *P* = .01; 1 RCT, n = 75)	NA
Mahdavi et al^54^ (SR) Rectal cancer	Prebiotics/Probiotics Dose: BID 108 CFU *Lactobacillus casei* PXN37, *L. rhamnosus* PXN54, *Streptococcus thermophilus* PXN66, *Bifidobacterium breve* PXN25, *L. acidophilus* PXN 35*l, B. longum* PXN30, *L. bulgaricus* PXN39 + FOS, magnesium stearate Duration: 6 wk	Placebo	QoL (EORTC QLQ-C30) ↔ in QoL NS (1 RCT, n = 38)	NA
Other nutrient supplements				
Fan et al^41^ (MA) Various cancers	Melatonin Dose: 10-20 mg/d Duration: 7 d to 1 yr, or until death	Placebo, light therapy	QoL (EORTC, FACT) SMD NS (6 RCTs, n = 967)	⊕OOO* Very low (due to very serious risk of bias and serious inconsistency and imprecision)
Mixed nutrient supplements				
Lin et al^42^ (MA) Various cancers	Interventions: L-carnitine 0.5 g/d (Day 1-2), 1 g/d (Day 3-4), 2 g/d ongoing; Biorinck chlorella granules 4 sticks/d; CoQ10 300 mg + vitamin E 300 IU TID; creatine monohydrate 20 g/d (first week), 5 g/d ongoing; kefir 250 ml BID; 4.3 g/d EPA + DHA Duration: NR	NR	QoL (FACT, FACIT, MDASI, SF-36, EORTC): Pooled data from all nutritional supplement interventions, MD NS (6 RCTs, n = 280)	
Fatigue				
Vitamins & minerals				
Hoppe et al^53^ (SR) Colorectal cancer	Zinc Dose: NR Duration: 16 wk during CT	Placebo	Fatigue (NR): NS (1 RCT, n = 24)	NA
Probiotics, prebiotics & synbiotics				
Belloni et al^79^ (SR) Colorectal and breast cancer	Prebiotics/Probiotics Dose/formulations: (i) *Lacidofil (L. rhamnosus* R0011 + *L acidophilus* R0052 2 × 109 CFUs) BID; (ii) *L. acidophilus* + *L. casei* + *L. lactis* + *B. longum* + *B. infantis* 30 × 10^9^ CFUs per sachet QI*D* + 2 g omega-FAs/d; (iii) *L. rhamnosus, L. casei, L. acidophilus, L. bulgaricus, B. breve, B. longum, L.helveticus, L.lactis, L.paraplantarum, B. bifidum, Streptococcus thermophilus, L.gasseri* 1 × 10^9^ CFUs BID + 21g FOS Duration: 8-12 wk before, during or after CT	Placebo	CRF (FACT-F, EORTC, CFS): (i) Lacidofil: ↑ FACT-F scores compared with placebo. At baseline, both groups had scores of 43.0 (CI for control: 36.0-49.0, CI for intervention 36.5-45.5) After 12 wk, intervention scores: ↑ 44.5, 95% CI 38.5-49.0, *P* = .02). Placebo 44.0, 95% CI 39.5-48.75, *P* < .05 (1 RCT, n = 66) (ii) ↓ EORTC-QLQ30 fatigue scores at 8 wk and 6 mo from baseline. Baseline scores: 23.7 ± 2.8; 8 wk scores: 11.97 ± 1.8; 6 mo scores: 10.3 ± 1.9 (*P* < .05). Placebo: ↑ EORTC-QLQ30 fatigue scores at 8 wk: 31.1 ± 3.0; and 6 mo: 35.4 ± 4.3 (*P* < .05). Baseline scores NR. (1 RCT, n = 140) (iii) ↓ CFS scores after 4 wk (*P* < .001) and was maintained at 8 wk (*P* < .001) compared with the placebo group who did not experience comparable improvement in fatigue symptoms. (1 RCT, n = 74)	NA
Deleemans et al^50^ (SR) Various cancers	Probiotics Dose: “Lacidofil” *L. rhamnosus* R0011, *L. acidophilus* R0052 2 × 109 CFU BID Duration: 12 wk	Placebo	Fatigue (FACT-F) ↓ FACT-F scores (*P* = .02; 1 RCT, n = 60)	NA
Tsai et al^78^ (SR/MA) Breast cancer	CoQ10 Dose: 300 mg/d Duration: 24 wk	Placebo	Profile of Mood States fatigue subscale (0-4) NS (1 RCT trial, n = 139)	NA
Other nutrient supplements				
Fan et al^41^ (MA) Various cancers	Melatonin Dose: 6-20 mg/d before bed Duration: 10 d to 1 y, or until death	Placebo, no treatment	Fatigue (EORTC, FACT-F, MFI-20, VAS): SMD NS (5 RCTs, n = 855)	Very Low ⊕OOO* (due to very serious risk of bias and imprecision)
Gremmler et al^62^ (SR) Pelvic cancer	Proteolytic enzymes Dose: 4 tablets Wobe-Mugos^®^ TID Duration: From 3 d prior to RT and during RT	Placebo	Fatigue (CTCAE, EORTC) ↔ differences in mild or moderate/severe fatigue NS (1RCT, n = 56)	NA
Hoppe et al^53^ (SR) Colorectal cancer	Zinc Dose: NR Duration: 16 wk during CT	Placebo	Fatigue (NR): NS (1 RCT, n = 24)	NA
Tsai et al^78^ (SR/MA) Breast cancer	CoQ10 Dose: 300 mg/d Duration: 24 wk	Placebo	Profile of Mood States fatigue subscale (0-4) NS (1 RCT, n = 139)	NA

Key: ↓ denotes significant decrease; ↑ denotes significant increase; ↔ non-significant/no difference.

Abbreviations: Arg, arginine; BID, twice per day; CFS, chronic fatigue scale; CFUs, colony-forming units; CI, confidence intervals; CoQ10, coenzyme Q10; CT, chemotherapy, d, day(s); CTCAE, NCI Common Terminology Criteria for Adverse Events; DHA, docosahexaenoic acid; EPA, eicosapentaenoic acid; EORTC QLQ-C30, European Organisation for Research and Treatment of Cancer Quality of Life Questionnaire; EORTC QLQ-CR29, QoL questionnaire for colorectal cancer; FAs, fatty acids; FACIT, The Functional Assessment of Chronic Illness Therapy-Fatigue; FACT, Functional Assessment of Cancer Therapy; FOS, fructooligosaccharides; GIQLI, Gastrointestinal Quality of Life Index; Gln, glutamine; HMB, beta-hydroxy b-methylbutyrate; MA, meta-analysis; MD, mean difference; MDASI, MD Anderson Symptom Inventory; MFI-20, Multidimensional Fatigue Inventory; mg, milligrammes; NA, not available; NR, not reported; NS, not significant; O3-FAs, omega-3 fatty acids; PHGG, partially hydrolysed guar gum; QLQ, QoL questionnaire; QoL, quality of life; RCT, randomised controlled trial; RT, radiotherapy; SF-36, 36-item Short Form Health Survey; SMD, standard mean difference; SR, systematic review; TID, three times daily; VAS, Visual Analogue Scale.

+ Original authors.

* Current authors.

Pre/probiotics may be effective for reducing CRF scores in breast and colorectal cancers compared with placebo^79^ as found in 3 RCTs (n = 280) evaluating diverse pre/probiotic formulations. One RCT demonstrating efficacy evaluated a combined intervention with probiotics and O3-FAs, however, the baseline fatigue scores of the intervention group were significantly higher than the control group, limiting the interpretation of this evidence (n = 140).^79^

All other evaluated interventions did not demonstrate efficacy against controls, including a meta-analysis on melatonin.^41^ Melatonin is not an effective intervention for CRF (5 RCTs, n = 855)^41^ with very low certainty. Zinc,^53^ proteolytic enzymes^62^ and coenzyme Q10 (CoQ10)^78^ were also not effective for improving CRF.

#### Genitourinary Complications

Two systematic reviews,^80,83^ with critically low methodological quality, evaluated the efficacy of nutrients for genitourinary complications in individuals with breast and cervical cancers (Table 5). Vaginal suppository intervention with a combination of HA, vitamin A and vitamin E resulted in statistically significant reductions in vaginal dryness, dyspareunia, vulvar fibrosis and mucosal inflammation among individuals with cervical cancer compared with control.^83^ However, the nature of the controls was not reported, and the evidence is limited to 2 RCTs (n = 222).^83^ Intervention with vitamin D or vitamin E resulted in a significant reduction in self-reported genitourinary symptoms in individuals with breast cancer compared with placebo.^80^ This evidence is also limited to a single RCT (n = 96), and the details of the intervention (dose, duration) were not reported.^80^

#### Hot Flushes

One network meta-analysis^90^ and 1 systematic review^63^ evaluated the incidence and severity of hot flashes in individuals with breast cancer (Table 5). The reviews were assessed as having critically low and low methodological quality. None of the evaluated interventions demonstrated efficacy. Supplementation with magnesium oxide (1 RCT, n = 358)^90^ and vitamin E (1 RCT, n = 125)^63^ showed no significant difference compared with placebo.

#### Insomnia/Sleep Disorders

We identified 1 meta-analysis, with low methodological quality, evaluating melatonin for sleep quality in individuals with various cancer diagnoses.^41^ Melatonin does not improve sleep quality compared with placebo and active controls with data pooled from 9 RCTs (n = 1128)^41^ with very low certainty. The effects remained nonsignificant following subgroup analyses according to intervention duration, dosage and treatment type^41^ (Table 6).

#### Lymphoedema

We identified 1 meta-analysis on probiotics^65^ and 1 systematic review on proteolytic enzymes^62^ that evaluated effects on lymphoedema in individuals with breast cancer (Table 5). The reviews were assessed as having low and critically low methodological quality. There were no interventions that demonstrated efficacy for improving lymphoedema outcomes.

Supplementation with probiotics *Lactobacillus*, *Bifidobacterium* and *Streptococcus* (species not reported) alone or combined with FOS were not effective for reducing oedema volume compared with placebo (2 RCTs, n = 223).^65^ Intervention with proteolytic enzymes also did not show any significant differences in arm volume, skin fold thickness or skin tension compared with placebo (1 RCT, n = 88).^62^

#### Mucositis

Eleven reviews reported on OM outcomes following nutritional supplement interventions (1 umbrella review,^57^ 1 network meta-analysis,^58^ 4 meta-analyses,^41,59-61^ and 5 systematic reviews^45,53,56,62,63^ (Table 4). Several interventions demonstrated promise for reducing the incidence and/or severity of OM during chemo/radiotherapy, including glutamine,^57^ melatonin,^41^ probioitics^59^ and zinc.^60^ There is limited evidence for supplementation with proteolytic enzymes^62^ and inconsistent evidence for vitamin E.^58,63^ The meta-analyses and systematic reviews were assessed as having low or critically low methodological quality.

There was low certainty that glutamine supplementation is effective for reducing the severity OM during radiotherapy compared with placebo or no treatment with pooled data from 16 RCTs (n = 1199).^57^

There was very low certainty evidence that melatonin supplementation is not effective for reducing the severity of stomatitis (3 RCTs, n = 154), nor was it effective for reducing the overall incidence of stomatitis (7 RCTs, n = 832).^41^ However, melatonin was found to be effective for reducing incidence of stomatitis when individuals with head and neck cancer were excluded in a subgroup analysis (4 RTCs, n = 674).^41^

There is very low certainty evidence that zinc supplementation, encompassing various dosage forms, may be effective in preventing OM compared with placebo (12 RCTs, n = 783).^60^ Following subgroup analysis by cancer location, zinc was not effective for reducing the incidence of OM in individuals with head and neck cancer (6 RCTs, n = 339).^60^

Three reviews evaluated the efficacy of vitamin E.^57,58,63^ In a network meta-analysis by Wang et al^58^ vitamin E mouthwash was superior to placebo (1 RCT, n = 59) and showed comparable efficacy to the other active treatments (eg, sodium bicarbonate, natural drugs; Table 4) for reducing the severity of OM in individuals with head and neck cancer (13 RCTs, n = 570). Topical application of vitamin E paste was also shown to be superior to both oral vitamin E supplementation and placebo paste for reducing the severity of OM after cycles 3 and 4 of chemo/radiotherapy in haematological cancer (1 RCT, n = 67).^63^ Oral vitamin E supplementation is effective for reducing OM incidence^57^ (2 RCTs, n = 72) with low certainty. A single RCT (n = 535) reports that beta-carotene “reduces mucositis” in head and neck cancer,^45^ however, the dosage and data to support this conclusion was not provided.

Among the other evaluated supplements, probiotic supplementation covering a diverse range of strains and dosage forms (eg, lozenges, Yakult) was effective for reducing the overall incidence of OM during chemotherapy compared with placebo or usual care^59^ (4 RCTs, n = 412). The authors, however, did not find any significant effects on OM severity (7 RCTs, n = 647).^59^ Oral proteolytic enzymes demonstrated efficacy for reducing the overall severity of OM in individuals with head and neck cancers compared with placebo in 1 review (3 RCTs, n = 208).^62^

Fucoidan^56^ and selenium^61^ did not demonstrate efficacy for OM outcomes.

#### Neuropathy

We identified 8 reviews (6 meta-analyses^64,68-72^ and two systematic reviews^40,63^) reporting on nutritional supplements to manage CIPN (Table 6). Vitamin E, glutamine, O3-FAs and NAC may reduce the incidence of CIPN however, the evidence was very uncertain. There is limited evidence that supplementation with various amino acids/amino acid derivatives, vitamin E and a B vitamin complex may reduce severity of CIPN. All reviews were assessed as having critically low to low methodological quality.

Vitamin E supplementation may be effective for reducing the incidence, but not severity, of CIPN (8 RCTs, n = 307)^69^ with very low certainty. The type of control was also not reported.^69^ In a systematic review by Retzlaff et al,^63^ compared with no treatment, vitamin E was significantly associated with a reduction in both the incidence and severity of CIPN and showed significant differences in sensory nerve function (2 RCTs, n = 62).

O3-FA supplementation may reduce the likelihood of developing CIPN by 80% compared with placebo across various cancer diagnoses (3 RCTs, n = 158, OR: 0.20, 95% CI: 0.10-0.40, *P* < .00001)^64^ with very low certainty.

There is very low certainty evidence that glutamine may reduce the incidence of CIPN (1 RCT, n = 86)^68^ and low certainty evidence that it may reduce severity (grade 3-4; 1 RCT, n = 86),^72^ when compared with standard care and non-active controls. Among the other amino acid/derivative interventions, there was moderate certainty that supplementation with NAC (2 RCTs, n = 46) and low certainty that L-carnosine (2 RCTs, 121) may be effective for reducing the severity of CIPN in individuals with gastrointestinal cancers compared with placebo or non-active controls.^72^ However, in another single RCT, supplementation with NAC did not prevent CIPN in individuals with breast cancer (1 RCT, n = 65).^70^ Cysteine and theanine supplementation reduced incidence of CIPN in gastrointestinal cancer (1 RCT, n = 28)^72^ with very low certainty.

Finally, supplementation with a B vitamin complex was associated with significant improvements in patient perceived sensory CIPN after 36 weeks of treatment (1 RCT, 71), however there were no differences in the overall incidence of CIPN in various cancer types.^40^

Other interventions with acetyl-L-carnitine,^40,71^ glutamate,^70^ and alpha-lipoic acid^40^ did not reduce incidence or severity of CIPN.

#### Postoperative Ileus

We identified 2 meta-analyses^67,81^ and one systematic review^82^ evaluating perioperative probiotic supplementation for the prevention of postoperative ileus in individuals with gastrointestinal cancers (Table 4). All the reviews were assessed as having critically low or low methodological quality.

There is very low certainty that probiotics may reduce time to flatus (7 RCTs, n = 693)^67^ and time to first stool (4 RCTs, n = 398).^81^ The systematic review by Cogo et al^82^ included 7 RCTs evaluating post-operative ileus with inconsistent findings across the interventions. The inconsistency is related to the diversity of the probiotic interventions; this heterogeneity precluded the authors from performing a meta-analysis.^82^ The details of the probiotic interventions are shown in Table 4.

#### Quality of Life

Seventeen reviews reported on the efficacy of nutrient supplementation for improvements in QoL including 4 meta-analyses^41-44^ and 13 systematic reviews^40,45-56^ (Table 7). Overall, perioperative pre/probiotic supplementation may improve QoL in individuals with colorectal cancer. There was less evidence for amino acids/amino acid derivatives, combined nutritional and phytochemical supplementation and O3-FAs in various cancer types. All included reviews on QoL were assessed as having critically low or low methodologic quality, apart from Croisier et al^48^ evaluating inulin and FOS in gynaecological cancer which was assessed as high quality.

There is limited and inconsistent evidence on pre/probiotics for improving QoL. Two systematic reviews found significant improvements in QoL in individuals with colorectal cancer using pre/probiotic interventions, irrespective of strain and formulation, when compared with placebo or no treatment (5 RCTs, n = 410).^50,52^ Prebiotic FOS-enriched jelly (1 RCT, n = 32) was also found to be effective for maintaining QoL (Gastrointestinal Quality of Life Index [GIQLI]) in individuals with head and neck cancer compared with placebo.^50^ However, supplementation with synbiotics, a combination of pre and probiotics,^54^ and *Lactobacillus plantarum*^50^ were not effective for improving QoL in individuals with colorectal cancer. Lactobacillus acidophilus combined with Bifidobacterium longum,^50^ prebiotic fibres including partially hydrolysed guar gum (PHGG),^50^ inulin and FOS^48^ did not demonstrate efficacy against control across individuals with various cancer diagnoses and treatment schedules.

A single RCT evaluating the amino acid derivative, L-carnitine (n = 72) demonstrated efficacy for improving QoL compared with placebo during chemotherapy in individuals with pancreatic cancer.^47^ A combined nutritional supplement (b-carotene, vitamin C, vitamin E, and selenium) may significantly improve QoL in individuals with cervical cancer undergoing chemoradiotherapy (1 RCT, n = 103),^45^ while CoQ10 and melatonin,^41^ vitamin E^44^ and zinc^53^ did not demonstrate efficacy.

O3-FA supplementation was reported to significantly improve QoL in individuals with breast cancer (1 RCT, n = 44),^51^ however was not effective in colorectal cancer (3 RCTs, n = 332)^43^ or mixed cancer types (none in breast cancer; 6 RCTs, n = 33).

A range of other evaluated nutritional supplement interventions did not demonstrate efficacy for QoL. Pooled data from trials evaluating L-carnitine, chlorella, coenzyme Q10 with vitamin E, creatine monohydrate and kefir showed no significant differences in QoL.^42^ Fucoidan,^56^ melatonin^41^ vitamin E^44^ zinc^53^and prebiotics, inulin and FOS^48^ did not demonstrate efficacy against control.

#### Xerostomia

Five systematic reviews^45,51,62,63,80^ reported on xerostomia outcomes following nutritional supplementation (Table 4). Interventions included proteolytic enzymes^62^ (1RCT), O3-FAs^51^ (1 RCT), selenium^80^ (1 RCT) and vitamin E alone^63,80^ (2 RCTs) or in combination with vitamin C^45^ (1 RCT). All of the evaluated nutritional supplements demonstrated efficacy for improving xerostomia,^45,51^ dysphagia^62^ or salivary gland function outcomes.^63,80^ This evidence, however, is limited to data reported from single RCTs with small sample sizes and deficient reporting on point estimates and measures of variability. All the reviews were assessed as having low or critically low methodological quality.

Supplementation with O3-FAs in breast cancer (1 RCT, n = 52)^51^ and vitamin C with vitamin E in head and neck cancer (1 RCT, n = 52)^45^ during chemotherapy were associated with significant improvements in xerostomia compared with placebo controls. In individuals with head and neck cancer, proteolytic enzymes also showed a significant reduction in dysphagia during radiotherapy compared with placebo (1 RCT, n = 100).^62^ In individuals with thyroid cancer, vitamin E supplementation during chemo/radiotherapy (2 RCTs, n = 118)^63,80^ and selenium during treatment with radioactive iodine were associated with significant improvements in salivary gland function compared with placebo (1 RCT, n = 16).^80^

#### Safety and Adverse Events

Eighteen of the included reviews reported on nutritional supplement-related adverse events AEs, including 5 meta-analyses^44,64,70,85,86^ and 13 systematic reviews^40,46,48,49,53,55,62,63,76,80,87-89^ (Table 8). Among the reviews that reported AEs, serious side effects (grade 3 or 4) were consistently reported for interventions with high-dose vitamin A, while AEs of zinc and vitamin E supplementation were varied. In a 2020 systematic review by Retzlaff et al^87^ oral vitamin A doses of 50 000, 100 000 and 300 000 IU/day were associated with a higher incidence of mucocutaneous side effects including skin rashes and desquamation, hepatic side effects including raised liver enzymes and increased triglycerides, and grade 3 or 4 gastrointestinal side effects, and bone pain compared with placebo controls (4 RCTs, n = 3335). Yellowing of the skin, bone pain, depression, diarrhoea and mucositis were associated with 50 mg and 75 mg beta-carotene intervention doses compared with placebo (n = 2 RCTs, 378).^87^ Yellowing of the skin was also reported following a placebo-controlled intervention with 300 mg beta-carotene and 400 IU vitamin E (1 RCT, n = 79).^76^ In a 2021 systematic review by Hoppe et al,^53^ 135 mg of zinc/day was associated with an increased incidence of moderate to severe dysphagia (1 RCT, n = 159), increased mild gastrointestinal side effects were reported in 3 additional RCTs (n = NR) while 7 other RCTs and 1 meta-analysis within the review found there were no side effects or differences between groups following zinc supplementation.^53^

**Table 8.** Nutritional Supplement Safety and Adverse Events.

Author, study design	Intervention	Control	Nutrition supplement-related adverse events
AMINO ACIDS & DERIVATIVES			
E Vasconcelos et al^76^ (SR) Head and neck cancer	HMB HMB/Arg/Gln Dose: 24 g HMB/Arg/Gln (1.2 g HMB, 7 g Arg, 7 g Gln) BID Duration: During CT/RT until 1 wk after CT/RT	No treatment	HMB/Arg/Gln related adverse events: No AEs reported (1 RCT, n = 34)
Leen et al^70^ (MA) Breast cancer	NAC Dose: Low dose group: 600 mg NAC BID High dose group: 1200 mg NAC BID Duration: 12 wk	No treatment/usual care	NAC-related adverse events (1) Withdrawn from the study: Watery diarrhoea and abdominal pain: n = 1 hypersensitivity reaction with skin rash: n = 2 (2) Other AEs: Constipation: n = 4, Nausea and vomiting: n = 8 Transient skin rash: n = 5 (1 RCT, n = 65)
Prado et al^55^ Various cancers	HMB/Arg/Gln Dose: 1.5 g HMB, 7 g Arg, 7 g Gln BID Duration: 8 wk during CT	Isonitrogenous, isocaloric placebo	Incidence of gastrointestinal AEs was ≤15%, with a probable relationship with HMB/Arg/Gln supplementation (1 RCT, n = 197)
MINERALS			
Barnhart et al^80^ (SR) Prostate, CLL, bladder cancer	Selenium, selenium + lycopene Dose: NR Duration: NR	NR	Selenium ± lycopene related AEs: ↔ in safety endpoints for selenium + lycopene (1RCT, n = 209, prostate cancer)
	Selenium Dose: NR Form(s): sodium selenite, seleno-I-methionine, or Se-methylselenocysteiene, oral selenium yeast Duration: NR	Placebo	Selenium related AEs: 1 patient discontinued due to Grade 2 constipation “Low levels of DNA damage” “Overall, well tolerated” (1 RCT, n = 24, leukaemia [CLL]) Similar side effects for selenium yeast (1 RCT, n = 292, bladder cancer)
Hoppe et al^53^ (SR) Various cancers	Zinc Dose: 45 mg TID Duration: NR	Placebo	Zinc-related adverse effects: Increased occurrence of moderate and severe dysphagia (7% zinc arm vs. 4% placebo arm) with 45 mg zinc TID (1 RCT, n = 159) Nausea and vomiting with 50 mg zinc sulphate TID (2 RCTs, n = NR) Diarrhoea, abdominal pain, cramps and diaphoresis (1 RCT, n = NR) 4 RCTs reported ↔ between groups (n = NR) 3 RCTs and 1 MA did not find any side effects from zinc supplementation (n = NR)
Zeng et al^89^ (SR) Head and neck	Zinc Dose: 50 mg zinc sulphate with meal TID Duration: 72 mo	Placebo	Zinc-related adverse effects: AEs between the two groups were not statistically significant (*P* = .67) (1 RCT, n = 72), head and neck cancer
Fatty acids			
Lam et al^64^ (MA) Mixed cancer types	O3-FAs Dose: 1.0-2.58 g EPA + 0.5 to 1.38 g DHA omega-3 PUFA-enriched oral capsules/d Duration: 9-24 wk	Placebo	Omega-3 FA related adverse events: ↔ in biochemical, haematological, mucosal, skin or gastrointestinal AEs (2 RCTs, n = 100). Intervention: grade 1 (mild symptoms) for diarrhoea at post-intervention, p NR (1 RCT, n = 56)
Phytochemicals			
Bagherniya et al^46^ (SR) Prostate cancer	Curcumin Dose: 1440-3000 mg curcumin/d; 120 mg nanocurcumin/d; 180 mg high absorbance curcumin Duration: 3 d prior to 6 mo post RT	Placebo, standard care	Curcumin-related adverse events: 4 RCTs reported no AEs to various curcumin interventions (n = 222)
da Anunciacao et al.^49^ (SR) Various cancers	Curcumin Dose: 1-3 g curcumin capsules/d Duration: During RT, RT/CT or CT	Control (dicalcium phosphate, rice bran, watercress extract, NR)	Curcumin-related adverse events: AEs were reported in both groups with ↔ (3 RCTs, n = 196)
E Vasconcelos et al^76^ (SR) Breast cancer	Anthocyanins Dose: 125 mg/d Duration: 1 wk prior to RT until 2-5 wk post	Placebo	Anthocyanin related adverse events: No AEs reported (1 RCT, n = 192)
E Vasconcelos et al^76^ (SR) Breast cancer	Adlay Bran Dose: 1 g Adlay bran extract capsules BID Duration: 5 to 6 wk	Olive oil capsules	Adlay bran related adverse events: Intervention group: Abdominal bloating (1/73) mild watery stools (1/73) between group difference NR (1 RCT, n = 110)
Probiotic, prebiotic, synbiotic			
An et al^85^ Colorectal cancer	Synbiotics/probiotics Strain(s): (i) *Lactobacillus acidophilus NCFM + L. rhamnosus HN001 + L. casei LPC-37 + Bifidobacterium lactis HN019 + fructooligosaccharides;* (*ii) B. longum + L. acidophilus + Enterococcus faecalis;* (*iii) B. animalis + B. lactis + L. casei + L. plantarum; iv) lactic acid bacteria; v) Streptococcus thermophilus + Bifidobacteria +L. acidophilus + L. plantarum + L. paracasei + L. delbrueckii subsp. Bulgaricus* Dose: NR Duration: 1 d prior to surgery to 21 d post-surgery. 1 RCT timed with antibiotic use: 1 d after discontinuation for 4 wk	Placebo, standard care	Probiotic-related adverse events: No significant difference in AEs between groups RR = NS (7 RCTs n = 692) RR: 0.73 (95% CI: 0.4501.19, *I*^2^ = 0%; low CoE) MCID: 5% absolute difference ⊕⊕OO+ Low (downgraded for imprecision and allocation [2 levels])
Croisier et al^48^	Prebiotic/probiotic Dose: 150 ml yoghurt with 6.5% lactulose + 2 × 109 L*actobacillus acidophilus*/d Duration: 5-7 wk, starting 5-7 d before RT	Placebo	Pre/probiotic-related adverse events 2 RCTs reported GI toxicity following supplementation (n = 410) 1 RCT reported increased flatulence in the intervention group and postulated the reason was the increased amount of lactulose consumed” (n = 21)
Vitamins			
*Vitamin E*			
Loprinzi et al^40^ (SR) Cancer type NR	Vitamin E Dose: 300-600 mg/d Duration: NR	NR	Vitamin E related adverse events: No AEs observed (4 RCTs, n = NR)
Nogueira et al^44^ (SR/MA) Cancer type NR	Vitamin E + pentoxifylline (PTX) Dose: 300-1000 mg per d Duration: 6 mo	Placebo or no intervention	Vitamin E related adverse events: Nausea (resolved after 1 wk), rash, treatment interruption n = 1 participant (1 RCT, n = 53), Both groups: nausea, bruising, neuropathic pain, thyrotoxicosis, bleeding from the conjunctiva, vomiting, gastritis, diarrhoea, gastrointestinal disorder, depression, dizziness, tiredness, insomnia, investigations, weight loss, headache, and increased sweating (1 RCT, n = 83). Vitamin E + PTX: hot flashes, asthenia, vertigo and headache Placebo and vit. E groups: No AEs (1 RCTS, n = 24).
Retzlaff et al^63^ (SR) Head and neck cancer	Vitamin E Dose: 400 mg a-tocopherol BID Duration: from day 1 to 4 of CT until 1 mo post CT	Placebo	Vitamin E related adverse events: ↔ AEs. Nausea, vomiting, alopecia was not related to the a-tocopherol intervention (2 RCTs, n = 72, *P* = .88, *P* = .96). Toxicities were likely associated with systemic CT treatment.
*Vitamins A, B, C & D*			
Retzlaff et al^87^ (SR) Various cancers	Vitamin A Form/dose: (i) Busulphan + vitamin A Aquasol A (retinol until 1982, retinyl palmitate thereafter) 50 000 IU/d; (ii) vitamin A 100 000 IU/d Duration: 18 mo and 7 y	Bulsulphan, no treatment	Vitamin A related adverse events: (i) Higher incidence of hepatic side effects (increased AST, GGT, and increased triglycerides (1 RCTs, n = 153) - incidence of ≥ grade 2 toxicity side effects (CTCAE) intervention vs control: 13/56 (23.2%) vs. 3/67 (4.5%; *P* = .002; 1 RCT, n = 153) (ii)53/118 (45%) in intervention experienced a side effect (1 RCT, n = 248 16/118 (14%) experienced a grade 3 or 4 side effect (1 RCT, n = 248) 17/118 (14.4%) dropped out of the intervention due to side effects (1 RCT, n = 248)
Zeng et al^89^ (SR) Skin cancer, breast cancer	Vitamin B3 (Nicotinamide) Dose: 1000 mg (Insolar, Blackmores)/d Duration: 18 mo	Placebo	Nicotinamide related adverse events: ↔ for number or AE type (1 RCT, n = 386), skin cancer
van Gorkom et al^88^ (SR) Advanced colorectal cancer	Vitamin C Duration: NR Dose: 10 g per oral/d	Placebo	Vitamin C related adverse events: Low incidence of mild AEs (1 RCT, n = 100). Type of AE NR
Zeng et al^89^ (SR) Skin cancer, breast cance	Vitamin D3 Dose: 3 capsules 10 000 IU/wk Duration: 24 wk	Placebo	Vitamin D related adverse events: No AEs to vitamin D3 were reported (1 RCT, n = 160), breast cancer
Other nutrient supplements			
E Vasconcelos et al^76^ (SR) Breast cancer	Adlay Bran Dose: 1 g Adlay bran extract capsules BID Duration: 5-6 wk	Olive oil capsules	Adlay bran related adverse events: Intervention group: Abdominal bloating (1/73) mild watery stools (1/73) between group difference NR (1 RCT, n = 110)
Gremmler et al^62^ (SR) Ovarian cancer	Proteolytic enzymes – containing papain, trypsin, chymotrypsin Dose: (i) 2 tablets Wobe-Mugos^®^ TID, (ii) 10 Wobe-Mugos^®^ dragees TID Duration: Day 2-7 of every CT cycle	Placebo	Oral enzyme related adverse events: Subjective global assessment: showed 97% of patients found the tolerability “very good.” One patient in the group (i) reported “undesirable side effects” (1 RCT, n = 59)
Seo et al^86^ (MA) Breast cancer	Melatonin Dose: 3-20 mg Duration: 10 d prior to and during CT to 4 mo post treatment	Placebo	Melatonin related adverse events: “Headache, fatigue, and bad dreams” (2 RCTs, n = 143) “No differences in side effects were found” (1 RCT, n = 48) “reduced side effects” (1 RCT, n = 36)

Abbreviations: AE, adverse events; Arg, arginine; BID, twice per day; CLL, Chronic lymphocytic leukaemia; CT, chemotherapy; d, day; DHA, docosahexaenoic acid; EPA, eicosapentaenoic acid; Gln, glutamine; HMB, beta-hydroxy b-methylbutyrate; PUFA, polyunsaturated fatty acid; TID; three times a day; MA, meta-analysis; n, number; NAC, N-acetylcysteine; NMA, network meta-analysis; NR, not reported; SR, systematic review; PTX, pentoxifylline; RCT, randomised controlled trial; RT, radiotherapy; TID, three times per day.

Three reviews reported on AEs to vitamin E.^40,44,63^ Two reviews (5 RCTs, n = NR) reported there were no differences between groups with intervention doses between 300 and 600 mg/day.^40,63^ Conversely, a systematic review and meta-analysis by Nogueira et al^44^ identified a higher incidence of mild gastrointestinal, haematological, and neurological side effects with doses between 300 and 1000 mg vitamin E/day compared with placebo or no intervention (3 RCTs, n = 160).

Minimal mild side effects, primarily gastrointestinal origin, were reported for supplementation with adlay bran,^76^ selenium,^80^ curcumin^46^, HMB/Arg/Gln,^55^ NAC,^70^ prebiotics,^48^ vitamin C,^88^ and proteolytic enzymes.^62^ Melatonin supplementation was associated with headaches, fatigue and bad dreams in 2 of 4 included RCTs that reported on AEs in a systematic review by Seo et al.^86^

No differences between groups for AEs were reported for supplementation with anthocyanins,^76^ fucoidan,^56^ O3-FAs,^64^ vitamin B3,^89^ vitamin D^89^ or pro/synbiotics.^85^ There is low certainty evidence that perioperative pro/synbiotics supplementation in individuals with colorectal cancer is associated with a 5% minimal clinically important difference in AEs compared with placebo or standard care in perioperative colorectal cancer (7 RCTs, n = 692).^85^ All data extracted on safety and AEs to the nutritional supplement interventions are shown in Table 8.

## Discussion

To our knowledge, this is the first umbrella review to comprehensively collate and evaluate the evidence on the use and safety of nutritional supplements in supportive cancer care. We synthesised the findings from 52 reviews covering a wide range of cancer and treatment-related effects, including any reported adverse events associated with the supplement intervention. Overall, most included reviews were of low or critically low methodological quality. While some interventions suggested benefit, evidence was largely uncertain or very uncertain, and a number of interventions did not suggest benefit, including for QoL.

We report that amino acids^73^ and oral proteolytic enzymes^75^ may reduce the incidence and severity of RD. Certainty of evidence varied from very low certainty for HMB/Arg/Gln for incidence to moderate certainty for severity of RD.^73^ Supplementation with exogenous enzymes may accelerate skin barrier repair in conditions such as epidermal hyperplasia^91^ and their use as a cosmetic application for skin renewal have gained attention.^92^ Our review updates evidence from the MASCC clinical practice guidelines for RD which did not recommend glutamine due to insufficient evidence of benefit.^39^ Although our updated evidence is based on a greater number of RCTs (5 RCTs vs 2 RCTs in the MASCC guidelines) we note that evidence remains limited and inconsistent, with different studies reporting both harm and benefit for glutamine supplementation,^75^ and therefore that strong clinical recommendations cannot be made at the present time.

Our review updates the older evidence on O3-FAs for prevention of weight loss, cited in the European Society for Clinical Nutrition and Metabolism (ESPEN) clinical practice guidelines for nutrition in cancer.^93^ The ESPEN guidelines cited 2 systematic reviews published in 2007 and 2015 and made a weak recommendation for O3-FAs to stabilise or improve appetite, food intake, lean body mass and body weight in individuals with advanced cancer undergoing chemotherapy and at risk of weight loss or malnutrition. Our review considers updated evidence from 2 more recent meta-analyses^43,64^ and 1 systematic review^51^ that report that O3-FAs are not effective for preventing weight loss in individuals with colorectal cancer, breast cancer and other cancer types, and includes new evidence from 7 additional RCTs. However, given this evidence was rated as very low certainty due to imprecision, risk of bias and inconsistency, we are uncertain as to the true effect of O3-FAs on prevention of weight loss. We note, as do the authors of the ESPEN guidelines, that there are no significant safety concerns with O3-FA administration and the balance of benefit versus harm may be in favour of O3-FAs.

Oral mucositis remains a debilitating and dose-limiting side effect of treatment. Glutamine,^57^ zinc,^60^ probiotics^59^ and melatonin^41^ may be effective for reducing the incidence of OM while glutamine may also reduce severity,^57^ however the certainty of evidence was low to very low. Melatonin, a hormone with potent free radical scavenger with antioxidant and anti-inflammatory properties,^94^ may protect against chemo/radiotherapy-induced mucosal damage and support mucosal regeneration.^94^ However, the evidence suggests its efficacy is limited to non-head and neck cancers.^41^ Glutamine is the most abundant amino acid well-recognised for its indispensable role in rapidly proliferating cells,^95^ while zinc is a trace element abundant in the skin and necessary to maintain the physiological functions.^96^ Our review updates the recommendations in the 2020 MASCC/ISOO Clinical Practice Guidelines for OM,^97^ which was based on older evidence (search date June 2016). Our findings concur with and strengthen the evidence that supports the use of oral glutamine (doses 10-30 mg/day) for individuals with head and neck cancer.^97^ In the same guidelines, zinc was no longer recommended for prevention due to inconsistent and insufficient evidence,^98^ and our review similarly concurs with these recommendations with regard to head and neck cancer, finding that there is no evidence for benefit in subgroup analysis.^60^ However, in our review, a pooled meta-analysis of all cancer types suggested very low certainty evidence of potential benefit for zinc, with particular promise for leukaemia and pharyngeal cancers in subgroup analyses.^60^ The use of zinc for OM in individuals with these cancers should be further evaluated.

Chemotherapy-induced peripheral neuropathy is a dose-limiting toxicity with limited treatment options.^99^ We found very low certainty evidence that vitamin E, O3-FAs and amino acids may provide potential protective effects for CIPN,^40,44,63,64,69^ with no serious side effects reported for either intervention. Our findings concur with the ASCO 2020 guidelines that were unable to make recommendations as to nutrient supplements for prevention or management of CIPN. Although we present updated evidence, including moderate certainty evidence for potential benefit of NAC in gastrointestinal cancer,^72^ this evidence remains limited and sparse. Further research is required to determine the neuroprotective effect of these supplements, especially given the dearth of options available to individuals experiencing CIPN.

At the time of planning our umbrella review, guidelines on management of CRF had not yet been published, and therefore, this symptom was included in our synthesis. Our findings complement those reported in the recently published ASCO-SIO guideline update on the management of fatigue in adult cancer survivors.^25^ Unlike our review, the ASCO-SIO guideline is based on a synthesis of individual RCTs, rather than of systematic reviews or meta-analyses. We also report on some nutritional supplements not included in the ASCO-SIO guideline, including pre/probiotics^50,79^ and zinc.^53^ Taken together, our findings and those in the ASCO-SIO guideline do not suggest a benefit from nutritional supplements (including co-enzyme Q10) for managing CRF.^100^ Given the demonstrated effectiveness of other interventions, such as exercise, cognitive-behavioural therapy and mind-body therapies for fatigue during and after treatment, these approaches should be recommended instead of nutritional supplements for management of this debilitating condition.^100^

The safety of nutritional supplements during and after cancer treatment is a key concern of clinicians and their patients. Adverse events were inconsistently reported across the included reviews. However, where reported, they were generally minor and severe AEs were rare. Additionally, AEs tended to be dose-related. High-dose vitamin A was associated with significant toxicity (50 000, 100 000 and 300 000 IU/d),^87^ and high doses of zinc (≥135 mg/d) led to severe dysphagia, increased nausea and vomiting.^53^ These side effects are consistent with other pharmacological doses of zinc, which exceed the recommended tolerable Upper Intake Level of 40 mg/d, across studies in other patient populations where nausea, vomiting, epigastric pain, lethargy and fatigue were reported with doses ranging from 100 to 300 mg/d.^101^ Importantly, however, and not captured by this review, are any disturbances in mineral absorption with high doses of zinc. Zinc interacts with mineral absorption, competitively inhibiting calcium, manganese, copper, selenium and iron in high doses for extended durations, with iron and copper most concerning.^101,102^ Copper deficiency has been associated with zinc doses of >50 mg/d for >10 months.^101^ This highlights the need to evaluate the risk-benefit profile while also considering treatment duration and ongoing mineral status with pharmacological doses of zinc.

A key challenge of using nutritional supplementation in supportive cancer care is the balance between benefit and harm, in particular, the potential for interference with the intended effect of curative treatment.^103^ For example, NAC increases cellular levels of cysteine and glutathione, restoring the antioxidant potential of cells by replenishing glutathione.^104^ During radiotherapy, this is counter-productive to the intended effect of treatment with radiotherapy and may have the potential to reduce the efficacy of radiotherapy. These key clinical concerns should be addressed with improved reporting of AEs and longer-term follow-up of participants to determine any potential impact on efficacy of curative treatments.

Implications for practice: Strong clinical recommendations are not possible due to the overall uncertainty of the evidence. At the present time, we suggest that clinicians discuss the direction of evidence and the uncertainties that preclude strong recommendations and assist their patients to make decisions informed by this evidence.

Implications for research: There is a significant gap in research on debilitating conditions such as cognitive impairment, that should be addressed. There are promising findings for a number of interventions such as glutamine and other amino acids, zinc and vitamin E. However, findings are consistently downgraded for concerns such as risk of bias in included RCTs, imprecision due to small sample sizes and small numbers of trials, and inconsistency due to significant heterogeneity. There is a need for a collaborative, systematic and standardised approach to evaluating the potential efficacy of nutrient supplements in supportive care in cancer. Standardisation of dose and delivery type should be achieved so that findings are comparable across studies, and heterogeneity is minimised. Additionally, evaluation of the impact of different doses should be incorporated in order to determine the minimum effective dose. Comprehensive and longer-term evaluation of adverse events is essential. Researchers should also aim to use core outcome sets where available. Lastly, factors affecting implementation of the use of nutrient supplements in clinical practice should also be evaluated.

This umbrella review has several strengths, among which is the methodology encompassing inclusion of the most up-to-date literature, selection of only the highest levels of evidence available and the broad scope of outcomes examined. A rigorous process was followed to eliminate or minimise overlap. Only reviews reporting a relevant quality assessment were included for review, although the quality assessment for each review was not presented. We used validated appraisal tools (AMSTAR-2, GRADE) and prioritised outcomes with validated measures. However, the strength of the umbrella review hinges on the quality and rigour of the included studies, most of which were of low methodological rigour. Whilst the AMSTAR-2 quality appraisal tool considers causes of heterogeneity, the use of sensitivity analysis in meta-analyses and the impact of publication bias in all included reviews, these assessments were not presented individually. Our search was restricted to articles available in English, and due to resource limitations, grey literature and non-randomised trials were excluded, which may have resulted in a less comprehensive review. Due to the breadth of the outcomes, GRADE assessments were not feasible for all reported outcomes. Finally, heterogeneity in dosages, formulations, probiotics strains and numbers, treatment duration and control limit the synthesis of findings.

## Conclusion

Cancer survivors, depending on the patient population, stage of disease and cultural background, are highly motivated to use nutritional supplements to manage side effects, improve QoL and provide a sense of autonomy and self-advocacy.^105^ This evidence synthesis serves to fill an important gap in supportive cancer care by equipping healthcare providers with the best available evidence to clarify misconceptions, guide evidence-based shared decision-making and overcome the historic and persistent lack of disclosure to healthcare providers regarding supplement use.^105^ Despite the generally low risk of adverse effects with nutritional supplements, the low to very low certainty of the majority of evidence limits the ability to make firm clinical recommendations. Moving forward, the provision of high-quality, evidence-based, patient-centred care requires larger, high-quality RCTs that include patient-reported outcomes as well as clinician-reported outcomes. These studies should assess the potential impact, positive or negative, of nutritional supplements on cancer treatment.

## Supplemental Material

sj-docx-1-ict-10.1177_15347354251405267 – Supplemental material for The Efficacy and Safety of Nutritional Supplements for Cancer Supportive Care: An Umbrella Review and Hierarchical Evidence Synthesis Supplemental material, sj-docx-1-ict-10.1177_15347354251405267 for The Efficacy and Safety of Nutritional Supplements for Cancer Supportive Care: An Umbrella Review and Hierarchical Evidence Synthesis by Sarah Benna-Doyle, Suzanne Grant, Alison Maunder, Jing Liu, Melik Ibrahim, Adele Cave, Chhiti Pandey, Monica Tang, Eng-Siew Koh, Geoff Delaney, Deep Jyoti Bhuyan, Victoria Choi, Ki Kwon, Maria Gonzalez, Susannah Graham, Ashanya Malalasekera and Carolyn Ee in Integrative Cancer Therapies

sj-docx-2-ict-10.1177_15347354251405267 – Supplemental material for The Efficacy and Safety of Nutritional Supplements for Cancer Supportive Care: An Umbrella Review and Hierarchical Evidence Synthesis Supplemental material, sj-docx-2-ict-10.1177_15347354251405267 for The Efficacy and Safety of Nutritional Supplements for Cancer Supportive Care: An Umbrella Review and Hierarchical Evidence Synthesis by Sarah Benna-Doyle, Suzanne Grant, Alison Maunder, Jing Liu, Melik Ibrahim, Adele Cave, Chhiti Pandey, Monica Tang, Eng-Siew Koh, Geoff Delaney, Deep Jyoti Bhuyan, Victoria Choi, Ki Kwon, Maria Gonzalez, Susannah Graham, Ashanya Malalasekera and Carolyn Ee in Integrative Cancer Therapies
