# Supplementary material for: The Efficacy and Safety of Nutritional Supplements for Cancer Supportive Care: An Umbrella Review and Hierarchical Evidence Synthesis
Source: Integr Cancer Ther. 2026 Jan 3;25:15347354251405267. doi: 10.1177/15347354251405267 (PMC12764759; doi:10.1177/15347354251405267)
Supplement: sj-docx-1-ict-10.1177_15347354251405267 – Supplemental material for The Efficacy and Safety of Nutritional Supplements for Cancer Supportive Care: An Umbrella Review and Hierarchical Evidence Synthesis [file sj-docx-1-ict-10.1177_15347354251405267.docx]

**Supplementary Table S2-** MEDLINE search strategy

Ovid MEDLINE(R) ALL

1 exp Neoplasms/ or Neoplasms.mp. 4158725

2 exp Cancer Survivors/ or Cancer Survivors.mp. 29748

3 (cancer* or neoplas* or "*malignant neoplasm" or tumour or tumor or "cancer survivo*r*" or carcinoma* or oncolog* or malignant).ti,ab. 4134049

4 1 or 2 or 3 5440666

5 "chemotherapy induced nausea and vomiting".ti,ab. 2264

6 CINV.ti,ab. 1305

7 Nausea/ or nause*.ti,ab. 86666

8 Vomiting/ or vomit*.ti,ab. 101861

9 (emesis* or hyperemesis).ti,ab. 10167

10 (emetic* or emetogenic).ti,ab. 6023

11 ("chemotherapy induced peripheral neuropathy" or CIPN).ti,ab. 2077

12 ("post-operative ileus" or "post operative ileus").ti,ab. 253

13 exp Antineoplastic Agents, Hormonal/ or Antineoplastic Agents, Hormonal.mp. 189725

14 "endocrine therapy side effects".ti,ab. 6

15 "endocrine therapy toxicity".ti,ab. 3

16 Menopause, Premature/ 1384

17 "early menopause".ti,ab. 1364

18 exp "Sexual Dysfunction, Physiological"/ or "Sexual Dysfunctions, Psychological"/ 39388

19 "sexual dysfunction".ti,ab. 13460

20 "genitourinary syndrome".ti,ab. 654

21 Dyspareunia/ or dyspareunia.ti,ab. 6214

22 "vaginal dryness".ti,ab. 1263

23 "vaginal atrophy".ti,ab. 762

24 Antineoplastic Agents/ae 50012

25 Hot Flashes/ or Climacteric/ 8760

26 "night sweat*".ti,ab. 3295

27 Andropause/ or andropause.ti,ab. 595

28 ("hot fl*she*" or "hot flashes").ti,ab. 5156

29 Hot Flashes/dt 1541

30 Vasomotor System/de 2848

31 ("vasomotor symptom*" or "night sweat*").ti,ab. 5697

32 Weight Gain/de 8884

33 Weight Loss/de 4325

34 ("weight gain" or "weight loss").ti,ab. 197097

35 Appetite/ or appetite.ti,ab. 37464

36 Asthenia/ or asthenia.ti,ab. 7128

37 Arthralgia/ or arthralgia.ti,ab. 18105

38 neuropath*.ti,ab. 172141

39 xerostomia/ or xerostomia.ti,ab. 8927

40 "Quality of Life"/ or ("quality of life" or QOL).ti,ab. 516157

41 ("health related quality of life" or "health-related quality of life" or HRQL).ti,ab. 67168

42 Sleep/de or (sleep* or "sleep disorder*").ti,ab. 267888

43 "Sleep Initiation and Maintenance Disorders"/ or insomnia.ti,ab. 39608

44 Lymphedema/ or lymph*edema.ti,ab. 16738

45 Mucositis/ or mucositis.ti,ab. 13272

46 Dermatitis/ or dermatitis.ti,ab. 81804

47 Attention/de 5268

48 Breast Neoplasms/px 13500

49 Cognition/de 14592

50 Memory/de 15132

51 Psychomotor Performance/de 8210

52 Verbal Behavior/de 396

53 "fear of cancer recur*".ti,ab. 899

54 FOCR.ti,ab. 7

55 Heart/de or Heart/ or heart.ti,ab. 1087872

56 (myocyte or cardiomyocyte).ti,ab. 39094

57 Myocardium/de or Myocardium/ or (myocardium or myocardial).ti,ab. 540456

58 Cardiotoxicity/ or (cardiotoxicity or "cardiac tox*" or cardiotox*).ti,ab. 25255

59 Cardiomyopathies/ or exp Heart Diseases/ 1334538

60 (cardiopathic or cardiopathy).ti,ab. 3974

61 Arrhythmias, Cardiac/ or ("cardiac arrhythmias" or arrhythmia*).ti,ab. 144642

62 cardiac.ti,ab. 763776

63 arrhythmia.ti,ab. 54991

64 Fatigue/ 38464

65 ("cancer related fatigue" or "cancer-related fatigue" or fatigue* or tired* or exhaust* or CRF).ti,ab. 243008

66 "side effects".ti,ab. 297341

67 13 and 66 7017

68 Antineoplastic Agents/ or ("antineoplastic agent*" or "anti-neoplastic agent*").ti,ab. 346403

69 66 and 68 15663

70 Vitamins/ or vitamin*.ti,ab. 274531

71 Minerals/tu or Minerals/ or mineral*.ti,ab. 241673

72 Nutrients/ or Micronutrients/ or (nutrient* or micronutrient*).ti,ab. 235977

73 (multivitamin* or multi-vitamin*).ti,ab. 4870

74 Dietary Supplements/ or ("dietary supplement" or "food supplement").ti,ab. 89441

75 "nutritional supplement".ti,ab. 2594

76 "meal replacement".ti,ab. 559

77 "health supplement".ti,ab. 510

78 Fatty Acids/ or "*fatty acid*".ti,ab. 312038

79 Fatty Acids, Omega-3/ or ("omega 3 fatty acid*" or "omega-3 fatty acid*").ti,ab. 22823

80 Eicosapentaenoic Acid/ or ("Eicosapentaenoic Acid" or EPA).ti,ab. 30687

81 "omega 3 carboxylic acid".ti,ab. 8

82 ("omega 3" or omega-3).ti,ab. 21406

83 (promega or pro-mega).ti,ab. 876

84 (n3 or n-3).ti,ab. 127045

85 Fish Oils/ or "fish oil*".ti,ab. 14182

86 Docosahexaenoic Acids/ or ("Docosahexaenoic Acid*" or DHA).ti,ab. 26514

87 alpha-Linolenic Acid/ or ("alpha-Linolenic Acid*" or "alpha Linolenic Acid*" or ALA).ti,ab. 50848

88 Linoleic Acid/ or (" alpha linoleic acid*" or "alpha-linoleic acid*").ti,ab. 7307

89 Fatty Acids, Unsaturated/ 22667

90 Thioctic Acid/ or ("thiotic acid" or "alpha lipoic acid" or "alpha-lipoic acid" or "lipoic acid").ti,ab. 7246

91 Amino Acids/tu or Amino Acids/ or "amino acid*".ti,ab. 620640

92 Taurine/ or taurine.ti,ab. 18630

93 Arginine/ or arginine.ti,ab. 128214

94 Tryptophan/ or tryptophan.ti,ab. 70360

95 Methionine/ or S-Adenosylmethionine/ 33630

96 (methionine* or s-adenosylmethionine).ti,ab. 63114

97 (levomethionine or levo-methionine).ti,ab. 1

98 Selenomethionine/ or selenomethionine.ti,ab. 3490

99 methiolate.ti,ab. 6

100 Creatine/ or creatine.ti,ab. 54306

101 Cysteine/ or cysteine.ti,ab. 126342

102 (acetylcysteine or acetyl-cysteine).ti,ab. 19911

103 (N-acetylcysteine or NAC).ti,ab. 36757

104 Probiotics/ or probiotic*.ti,ab. 51735

105 Prebiotics/ or prebiotic*.ti,ab. 16045

106 symbiotic.ti,ab. 21944

107 Tocopherols/ or tocopherol*.ti,ab. 24853

108 alpha-Tocopherol/ or ("alpha tocopherol" or alpha-tocopherol).ti,ab. 18670

109 Vitamin E/ or "vitamin E".ti,ab. 42655

110 Carotenoids/ or carotenoid*.ti,ab. 39502

111 carotene.ti,ab. 19869

112 Vitamin A/ or "vitamin A".ti,ab. 39289

113 ("retinyl palmitate" or retinyl*).ti,ab. 3560

114 "retinoic acid".ti,ab. 36532

115 Vitamin B Complex/ or ("B vitamin*" or "B group vitamin").ti,ab. 11979

116 Thiamine/ or (thiamine or "vitamin B1").ti,ab. 18083

117 Riboflavin/ or (riboflavin or "vitamin B2").ti,ab. 16639

118 Niacin/ or Niacinamide/ or ("nicotinic acid" or "vitamin B3" or niacin or niacinamide).ti,ab. 31695

119 Pantothenic Acid/ or ("pantothenic acid" or pantothenic or "vitamin B5").ti,ab. 4131

120 Vitamin B 6/ or Pyridoxine/ or Pyridoxal Phosphate/ 16172

121 ("vitamin B6" or pyridoxine or pyridox*).ti,ab. 20979

122 Biotin/ or (biotin or "vitamin B7" or "vitamin H").ti,ab. 37162

123 Folic Acid/ or "folic acid".ti,ab. 42676

124 methylfolate.ti,ab. 195

125 5-MTH.ti,ab. 15

126 "Levomefolic acid".ti,ab. 4

127 folate.ti,ab. 31949

128 "folinic acid".ti,ab. 3360

129 Methylfol*.ti,ab. 216

130 Vitamin B 12/ or "vitamin B12".ti,ab. 32551

131 cyanocobalamin.ti,ab. 1528

132 methylcobalamin.ti,ab. 851

133 cobalamin.ti,ab. 5164

134 Inositol/ or (inositol or myo-inositol).ti,ab. 44368

135 "ascorbyl palmitate".ti,ab. 305

136 Ascorbic Acid/ or "ascorbic acid".ti,ab. 65996

137 "vitamin C".ti,ab. 27812

138 ascorbate.ti,ab. 20204

139 Antioxidants/ or antioxidant*.ti,ab. 361870

140 Flavonoids/ or flavonoid*.ti,ab. 100074

141 bioflavonoid*.ti,ab. 1507

142 "vitamin P".ti,ab. 277

143 "ferulic acid".ti,ab. 7466

144 Cholecalciferol/ or cholecalciferol.ti,ab. 10641

145 Vitamin D/ or ("vitamin D" or "vitamin D3").ti,ab. 97564

146 Iron/ or iron.ti,ab. 276873

147 Ferrous Compounds/tu or Ferrous Compounds/ or ferrous.ti,ab. 24343

148 Trace Elements/ or "trace element*".ti,ab. 35897

149 Calcium/ or calcium.ti,ab. 575380

150 Phosphorus/ or phosphorus.ti,ab. 105009

151 Magnesium/ or magnesium.ti,ab. 117314

152 Potassium/ or potassium.ti,ab. 224892

153 Manganese/ or manganese.ti,ab. 58846

154 Zinc/ or zinc.ti,ab. 178129

155 Selenium/ or selenium.ti,ab. 43428

156 Boron/ or boron.ti,ab. 27930

157 Chromium/ or chromium.ti,ab. 38853

158 Lycopene/ or lycopene.ti,ab. 6991

159 Isoflavones/ or isoflav*.ti,ab. 17304

160 Genistein/ or genistein.ti,ab. 13416

161 "soy isoflav*".ti,ab. 2156

162 daidzein.ti,ab. 4309

163 Carnitine/ or carnitine.ti,ab. 21083

164 Acetylcarnitine/ or (acetylcarnitine or L-carnitine or acetyl-L-carnitine).ti,ab. 7375

165 Grape Seed Extract/ or Proanthocyanidins/ or ("grape seed extract" or proanthocyanidins).ti,ab. 7264

166 Quercetin/ or quercetin.ti,ab. 30666

167 quercitol.ti,ab. 48

168 ("indole-3-carbinol" or I3C).ti,ab. 1208

169 Isothiocyanates/ or isothiocyanate.ti,ab. 19800

170 (diindolymethane or DIM).ti,ab. 9051

171 sulforaphane.ti,ab. 3126

172 ("green tea extract" or EGCG).ti,ab. 9148

173 Vitamin K 2/ or "vitamin K2".ti,ab. 5138

174 Melatonin/ or melatonin.ti,ab. 34466

175 Glutathione/ or glutathione.ti,ab. 178261

176 Lignans/ or lignan.ti,ab. 9034

177 "fermented wheat germ".ti,ab. 62

178 Resveratrol/ or resveratrol.ti,ab. 19275

179 "aged garlic extract".ti,ab. 367

180 Carnosine/ or (carnosine or L-carnosine).ti,ab. 3318

181 Galactans/ or (galactan* or arabinogalactan).ti,ab. 5941

182 "rice bran extract".ti,ab. 108

183 Glycine/ or (glycine or L-glycine).ti,ab. 90373

184 Ubiquinone/ or ubiquinone.ti,ab. 15385

185 ("coenzyme Q10" or CoQ10 or co-Q10 or "coenzyme Q").ti,ab. 7294

186 ubiquinol.ti,ab. 2059

187 Pectins/ or (pectin* or "citrus pectin*").ti,ab. 19879

188 Coumaric Acids/ or Coumarins/ or ("coumaric acid" or coumarin*).ti,ab. 35085

189 Ellagic Acid/ or "ellagic acid".ti,ab. 4142

190 Polyphenols/ or polyphenol*.ti,ab. 57121

191 Catechin/ or catechin.ti,ab. 18586

192 Anthocyanins/ or Anthocyanin*.ti,ab. 20934

193 anthocyanidin.ti,ab. 1012

194 Tannins/ or tannin*.ti,ab. 19499

195 "tannic acid".ti,ab. 5893

196 gallotanin.ti,ab. 9

197 Rutin/ or rutin.ti,ab. 10188

198 rutinoside.ti,ab. 2044

199 rutoside.ti,ab. 245

200 Molybdenum/ or molybdenum.ti,ab. 22655

201 Lactoferrin/ or lactoferrin.ti,ab. 10546

202 Polysaccharides/ or polysaccharides.ti,ab. 85419

203 biobran.ti,ab. 39

204 arabinoxylan.ti,ab. 1613

205 beta-Glucans/ or (beta-glucan* or b-glucan).ti,ab. 11099

206 allicin.ti,ab. 1183

207 gingerols.ti,ab. 307

208 shogaols.ti,ab. 139

209 6-shogaol.ti,ab. 484

210 Curcumin/ or curcumin*.ti,ab. 26103

211 Saponins/ or saponin.ti,ab. 20435

212 Phytic Acid/ or "phytic acid".ti,ab. 5551

213 "chlorogenic acid".ti,ab. or Chlorogenic Acid/ 8453

214 *beta Carotene/ 3815

215 "beta carotene".ti,ab. 17205

216 *Retinoids/ 4206

217 retinoid*.ti,ab. 21094

218 "vitamin B8".ti,ab. 10

219 "phenolic acid".ti,ab. 2902

220 glucoraphanin.ti,ab. 461

221 "epigallocatechin-3-gallate".ti,ab. 3949

222 curcuminoid.ti,ab. 675

223 5 or 6 or 7 or 8 or 9 or 10 or 11 or 12 or 13 or 14 or 15 or 16 or 17 or 18 or 19 or 20 or 21 or 22 or 23 or 24 or 25 or 26 or 27 or 28 or 29 or 30 or 31 or 32 or 33 or 34 or 35 or 36 or 37 or 38 or 39 or 40 or 41 or 42 or 43 or 44 or 45 or 46 or 47 or 48 or 49 or 50 or 51 or 52 or 53 or 54 or 55 or 56 or 57 or 58 or 59 or 60 or 61 or 62 or 63 or 64 or 65 or 66 or 67 or 68 or 69 4555012

224 70 or 71 or 72 or 73 or 74 or 75 or 76 or 77 or 78 or 79 or 80 or 81 or 82 or 83 or 84 or 85 or 86 or 87 or 88 or 89 or 90 or 91 or 92 or 93 or 94 or 95 or 96 or 97 or 98 or 99 or 100 or 101 or 102 or 103 or 104 or 105 or 106 or 107 or 108 or 109 or 110 or 111 or 112 or 113 or 114 or 115 or 116 or 117 or 118 or 119 or 120 or 121 or 122 or 123 or 124 or 125 or 126 or 127 or 128 or 129 or 130 or 131 or 132 or 133 or 134 or 135 or 136 or 137 or 138 or 139 or 140 or 141 or 142 or 143 or 144 or 145 or 146 or 147 or 148 or 149 or 150 or 151 or 152 or 153 or 154 or 155 or 156 or 157 or 158 or 159 or 160 or 161 or 162 or 163 or 164 or 165 or 166 or 167 or 168 or 169 or 170 or 171 or 172 or 173 or 174 or 175 or 176 or 177 or 178 or 179 or 180 or 181 or 182 or 183 or 184 or 185 or 186 or 187 or 188 or 189 or 190 or 191 or 192 or 193 or 194 or 195 or 196 or 197 or 198 or 199 or 200 or 201 or 202 or 203 or 204 or 205 or 206 or 207 or 208 or 209 or 210 or 211 or 212 or 213 or 214 or 215 or 216 or 217 or 218 or 219 or 220 or 221 or 222 4329868

225 4 and 223 and 224 92229

226 ("systematic review*" or "meta-analysis" or "umbrella review" or "meta-review" or "meta-analy*" or metaanaly* or "meta reg*" or metareg*).ti,ab. 530756

227 exp Meta-Analysis/ 221006

228 exp "Systematic Review"/ 293883

229 226 or 227 or 228 567011

230 225 and 229 1742

231 limit 230 to (english language and yr="2017 - 2023") 833

232 limit 231 to dt=20230315-20230615 32

233 limit 230 to yr="2019 - 2024" 849

234 limit 233 to dt=20170101-20230615 607

235 limit 230 to dt=20170101-20240510 903

236 limit 235 to english language 891
