# Supplementary material for: The Efficacy and Safety of Nutritional Supplements for Cancer Supportive Care: An Umbrella Review and Hierarchical Evidence Synthesis
Source: Integr Cancer Ther. 2026 Jan 3;25:15347354251405267. doi: 10.1177/15347354251405267 (PMC12764759; doi:10.1177/15347354251405267)
Supplement: sj-docx-2-ict-10.1177_15347354251405267 – Supplemental material for The Efficacy and Safety of Nutritional Supplements for Cancer Supportive Care: An Umbrella Review and Hierarchical Evidence Synthesis [file sj-docx-2-ict-10.1177_15347354251405267.docx]

**Supplementary Table S2 –** List of excluded studies with exclusion reasons

| **Author Year (Reference)** | **Title** | **Exclusion reason** |
| --- | --- | --- |
| ***Reports excluded (n=52)*** | | |
| Abt 2020 ^1^ | Zinc Supplementation May Reduce the Effects of Oral Mucositis for Patients With Cancer Receiving Either Chemotherapy or Radiotherapy | Commentary on the review (review of the review) |
| Abt 2021 ^2^ | PROBIOTICS MAY LOWER THE RISK OF ORAL MUCOSITIS IN CANCER PATIENTS | Commentary on the review (review of the review) |
| Akbari 2020 ^3^ | Curcumin as a preventive or therapeutic measure for chemotherapy and radiotherapy induced adverse reaction: A comprehensive review | Wrong study design |
| Al-Hussaniy 2023 ^4^ | Chemotherapy-induced cardiotoxicity: a new perspective on the role of Digoxin, ATG7 activators, Resveratrol, and herbal drugs | Wrong study design |
| Alcantara 2020 ^5^ | Vitamin E in the treatment of chemotherapy and radiation-induced mucositis: A meta-analysis of randomized controlled trials | Abstract only |
| Blackwood 2020 ^6^ | A systematic review examining nutrition support interventions in patients with incurable cancer | Wrong intervention |
| Bowen 2019 ^7^ | Systematic review of agents for the management of cancer treatment-related gastrointestinal mucositis and clinical practice guidelines | Risk of bias not performed |
| Chaitanya 2019 ^8^ | Meta-analysis on the efficacy of zinc in oral mucositis during cancer chemo and/or radiotherapy-an evidence-based approach | Risk of bias not performed |
| Cogo 2021 ^9^ | Are supplemental branched-chain amino acids beneficial during the oncological peri-operative period: A systematic review and meta-analysis | Wrong intervention |
| Cogo 2023 ^10^ | A systematic review of probiotics use during oncological surgery | Abstract only |
| Crichton 2019 ^11^ | Efficacy of Ginger (Zingiber officinale) in Ameliorating Chemotherapy-Induced Nausea and Vomiting and Chemotherapy-Related Outcomes: A Systematic Review Update and Meta-Analysis | Wrong intervention |
| Daugelaite 2019 ^12^ | Prevention and Treatment of Chemotherapy and Radiotherapy Induced Oral Mucositis | Risk of bias not performed |
| DiNapoli 2023 ^13^ | What Is the Role of Nutraceutical Products in Cancer Patients? A Systematic Review of Randomized Clinical Trials | Risk of bias not performed |
| Elad 2020 ^14^ | MASCC/ISOO clinical practice guidelines for the management of mucositis secondary to cancer therapy | Wrong study design |
| Emanuel 2022 ^15^ | Nutritional Interventions in Pancreatic Cancer: A Systematic Review | Wrong intervention |
| Firoozbakhsh 2024 ^16^ | Cardioprotective potential of botanical agents against anthracycline-induced cardiotoxicity | Wrong study design |
| Garczyk 2022 ^17^ | Influence of Probiotics in Prevention and Treatment of Patients Who Undergo Chemotherapy or/and Radiotherapy and Suffer from Mucositis, Diarrhoea, Constipation, Nausea and Vomiting | Risk of bias not performed |
| Gardiner 2022 ^18^ | THE EFFICACY OF FIBRE AND PREBIOTIC INTERVENTIONS ON CLINICAL OUTCOMES IN CANCER AND HAEMATOPOIETIC STEM CELL THERAPIES: SYSTEMATIC LITERATURE REVIEW | Abstract only |
| Gholamalizadeh 2023 ^19^ | The effects of dietary supplements in patients with cervical cancer: a comprehensive systematic review | Risk of bias not performed |
| Ginzac 2020 ^20^ | Quality of life for older patients with cancer: a review of the evidence supporting melatonin use | Wrong study design |
| Jangid 2023 ^21^ | Non-Pharmacological Strategies for Chemotherapy-Induced Peripheral Neuropathy: A Comprehensive Review | Abstract only |
| Jayaraj 2020 ^22^ | Clinical approaches to interpreting the findings of systematic review and meta-analysis of the effectiveness of probiotics in the prevention and treatment of Cancer Therapy-Induced Oral Mucositis (CTIOM) | Commentary on the review (review of the review) |
| Kalisch 2019 ^23^ | Complementary therapies for the prevention and management of chemotherapy-induced peripheral neuropathy | Abstract only |
| KarabogaArslan 2022 ^24^ | The golden spice curcumin in cancer: A perspective on finalized clinical trials during the last 10 years | Wrong study design |
| Kemp 2020 ^25^ | Interventions to manage cardiovascular disease risk in cancer patients and survivors: An umbrella review | Abstract only |
| Khamdi 2023 ^26^ | Non-pharmacologic interventions for management of radiation-induced dry mouth: A systematic review | Wrong outcomes |
| Li 2023 ^27^ | The Role of Complementary and Alternative Medicine on Cancer-Related Fatigue in Adults: An Overview of Systematic Reviews | Wrong intervention |
| Lian 2021 ^28^ | Pyridoxine for prevention of hand-foot syndrome caused by chemotherapy agents: a meta-analysis | Wrong study design |
| Lim 2022 ^29^ | Effects of exogenous melatonin supplementation on health outcomes: An umbrella review of meta-analyses based on randomized controlled trials | Wrong patient population |
| Mansouri 2020 ^30^ | Clinical effects of curcumin in enhancing cancer therapy: A systematic review | Wrong outcomes |
| Mezerji 2020 ^31^ | Investigating the effect of herbal antioxidants on the process of colon cancer treatment: A systematic review | Wrong outcomes |
| MirzaeiDahka 2023 ^32^ | Impact of Curcumin Supplementation on Radiation Dermatitis Severity: A Systematic Review and Meta-Analysis of Randomized Controlled Trials | Risk of bias not performed |
| Mora 2022 ^33^ | Complementary and alternative medicine modalities used to treat adverse effects of anti-cancer treatment among children and young adults: a systematic review and meta-analysis of randomized controlled trials | Paediatric population |
| Moutabian 2022 ^34^ | The cardioprotective effects of nano-curcumin against doxorubicin-induced cardiotoxicity: A systematic review | Wrong study design |
| Muhamad 2023 ^35^ | The Role of Vitamin B6 in Peripheral Neuropathy: A Systematic Review | Wrong patient population |
| Muszynski 2022 ^36^ | Vitamin D-The Nutritional Status of Post-Gastrectomy Gastric Cancer Patients-Systematic Review | Wrong outcomes |
| Najafi 2020 ^37^ | The role of melatonin on doxorubicin-induced cardiotoxicity: A systematic review | Wrong study design |
| Nayeri 2020 ^38^ | The Effect of Complementary and Alternative Medicines on Quality of Life in Patients with Breast Cancer: A Systematic Review | Wrong intervention |
| Parsons 2023 ^39^ | Nutrition as prevention for improved cancer health outcomes: a systematic literature review | Results not sufficiently reported |
| Patel 2024 ^40^ | Evaluation of curcumin for dermatologic conditions: a systematic review | Wrong patient population |
| Perez-Bilbao 2023 ^41^ | Effects of Combined Interventions of Exercise and Diet or Exercise and Supplementation on Breast Cancer Patients: A Systematic Review | Wrong intervention |
| Robert 2022 ^42^ | Systemic therapies for preventing or treating aromatase inhibitor-induced musculoskeletal symptoms in early breast cancer (review) | Wrong outcomes |
| Srinivasaraghavan 2021 ^43^ | Effect of Whey Protein Supplementation on Perioperative Outcomes in Patients with Cancer-A Systematic Review and Meta-Analysis (PROSPERO 2020: CRD42020188666) | Wrong outcomes |
| Thomsen 2019 ^44^ | Zinc deficits, mucositis, and mucosal macrophage perturbation: is there a relationship? | Wrong study design |
| Ukovic 2020 ^45^ | Nutrition interventions to improve the appetite of adults undergoing cancer treatment: a systematic review | Wrong intervention |
| Vogel 2023 ^46^ | Phytoestrogen Treatment for Menopausal Vasomotor Symptoms after Breast Cancer | Wrong intervention |
| Wiese 2023 ^47^ | Green tea and green tea extract in oncological treatment: A systematic review. | Wrong intervention |
| Wolyniuk 2023 ^48^ | NUTRITIONAL INTERVENTIONS TO SUPPORT LUNG CANCER PATIENTS UNDERGOING THORACIC RADIATION: A SYSTEMATIC REVIEW | Abstract only |
| Wu 2024 ^49^ | Efficacy of turmeric in the treatment of oral mucositis in patients with head and neck cancer after radiotherapy or chemoradiotherapy: a systematic review of meta-analysis | Wrong intervention |
| Yarom 2019 ^50^ | Systematic review of natural and miscellaneous agents for the management of oral mucositis in cancer patients and clinical practice guidelines-part 1: vitamins, minerals, and nutritional supplements | Wrong study design |
| Yarom 2021 ^51^ | Correction to: Systematic review of natural and miscellaneous agents, for the management of oral mucositis in cancer patients and Clinical Practice Guidelines - Part 1: vitamins, minerals and nutritional supplements (Supportive Care in Cancer, (2019), 27, 10, (3997-4010), 10.1007/s00520-019-04887-x) | Commentary on the review (review of the review) |
| Zhang 2022 ^52^ | Can Dietary Nutrients Prevent Cancer Chemotherapy-Induced Cardiotoxicity? An Evidence Mapping of Human Studies and Animal Models | Wrong patient population |
| ***Studies excluded due to overlap (n=29)*** | | |
| Auttara-Atthakorn 2022 ^53^ | Prevention of salivary gland dysfunction in patients treated with radioiodine for differentiated thyroid cancer: A systematic review of randomized controlled trials | SR |
| Chan 2020 ^54^ | The effects of pharmacological and non-pharmacological interventions on symptom management and quality of life among breast cancer survivors undergoing adjuvant endocrine therapy: A systematic review | 100% overlap with Barnhart 2023 & Delmicon 2023 |
| Crichton 2022 ^55^ | Orally comsumed ginger and human health: an umbrella review | Only included review on cancer population in Crichton 2019 and is included |
| de Meneses 2020 ^56^ | Effects of oral supplementation in the management of oral mucositis in cancer patients: a meta-analysis of randomized clinical trials | 100% overlap with more recent MAs |
| Dharman 2021 ^57^ | A Systematic Review and Meta-Analysis on the Efficacy of Curcumin/Turmeric for the Prevention and Amelioration of Radiotherapy/Radiochemotherapy Induced Oral Mucositis in Head and Neck Cancer Patients | 75% overlap with other review |
| Edwards 2021 ^58^ | Nutritional interventions for oral mucositis: a systematic literature review | 100% coverage with newer MAs |
| Frey-Furtado 2023 ^59^ | The Role of Biotics as a Therapeutic Strategy for Oral Mucositis- A Systematic Review | 100% overlap with other reviews |
| Guo 2019 ^60^ | Complementary and alternative medicine for natural and treatment-induced vasomotor symptoms: An overview of systematic reviews and meta-analyses | 100% overlap with RCT on vit E in BC hot flashes by Retzlaff 2021 |
| Hao 2020 ^61^ | Effects of Nonpharmacological Interventions in Chemotherapy-Induced Peripheral Neuropathy: An Overview of Systematic Reviews and Meta-Analyses | 100% overlap with other reviews |
| Heilfort 2023 ^62^ | A Systematic Review of the Benefit of B-Vitamins as a Complementary Treatment in Cancer Patients | 100% overlap with other reviews |
| Jafari-Koulaee 2021 ^63^ | The effect of melatonin on sleep quality and insomnia in patients with cancer: a systematic review study | 100% overlap with more recent MAs (Seo 2023 & Fan 2022) |
| Kao 2023 ^64^ | Topical Prevention of Radiation Dermatitis in Breast Cancer Patients: A Network Meta-Analysis of Randomised Controlled Trials | NMA |
| Lee 2024 ^65^ | Efficacy of natural products in preventing oral mucositis resulting from cancer therapies: A network meta-analysis of randomized controlled trials | 75% overlap with MA by Wu 2024 |
| Liu 2022 ^66^ | Preventive Effect of Probiotics on Oral Mucositis Induced by Cancer Treatment: A Systematic Review and Meta-Analysis | CCA 43% with Feng 2022 |
| Lu 2022 ^67^ | Effects of probiotic supplementation on related side effects after chemoradiotherapy in cancer patients | probiotics |
| Minervini 2023 ^68^ | Probiotics in the Treatment of Radiotherapy-Induced Oral Mucositis: A Systematic Review with Meta-Analysis | Feng 2022 more comprehensive MA with 12 studies, n=3 in Minervini (2 of 3 in Feng 2022) |
| Normando 2019 ^69^ | Effects of turmeric and curcumin on oral mucositis: A systematic review | 100% overlap with other reviews |
| Osouli-Tabrizi 2023 ^70^ | The effectiveness of omega-3 fatty acids on health outcomes in women with breast cancer: A systematic review | Excluded due to after compared with Delmicon 2023, CCA>15, with less included relevant RCTs and the older search date. |
| Peng 2022 ^71^ | Effects of various treatments for preventing oral mucositis in cancer patients: A network meta-analysis | 100% overlap with AmiriKosroshani MA 2023 & Feng MA 2022. All but 1 RCTs on curcumin covered in MA by Wu 2024 |
| Pico-Monllor 2019 ^72^ | Search and Selection of Probiotics That Improve Mucositis Symptoms in Oncologic Patients. A Systematic Review | 100% overlap with MA by Feng 2022 |
| Ramos 2023 ^73^ | Therapeutic Potential of Melatonin Couteracting Chemotherapy-Induced Toxicity in Breast Cancer Patients: A Systematic Review | 25% CCA with MA by Fan 2022 which has more recent RCTs |
| Rodriguez-Arrastia 2021 ^74^ | Probiotic Supplements on Oncology Patients' Treatment-Related Side Effects: A Systematic Review of Randomized Controlled Trials | 83% overlap with other reviews |
| Shu 2020 ^75^ | The effectiveness of probiotics in prevention and treatment of cancer therapy-induced oral mucositis: A systematic review and meta-analysis | 80% overlap with MA by Feng 2022 |
| Shuai 2019 ^76^ | Prophylaxis With Oral Zinc Sulfate Against Radiation Induced Oral Mucositis in Patients With Head and Neck Cancers: A Systematic Review and Meta-Analysis of Four Randomized Controlled Trials | 100% overlap with MA by Liu 2023 |
| Van de Roovaart 2024 ^77^ | Safety and efficacy of vitamin B in cancer treatments: A systematic review | 100% overlap with Loprinzi 2020 & Pandy 2022 |
| Wen 2023 ^78^ | Update on the treatment of chemotherapy and radiotherapy-induced buccal mucositis: a systematic review | SR |
| Wilairat 2020 ^79^ | Comparative efficacy and safety of interventions for preventing chemotherapy-induced oral mucositis in adult cancer patients: a systematic review and network meta-analysis | 100% overlap with AmiriKosroshahi 2023 & Liu 2023 |
| Yu/Ya Ying 2020 ^80^ | Effects of 9 oral care solutions on the prevention of oral mucositis: a network meta-analysis of randomized controlled trials | 100% overlap with MA by Wu 2024 |
| Zhang 2021 ^81^ | Prophylactic and Therapeutic Effects of Curcumin on Treatment-Induced Oral Mucositis in Patients with Head and Neck Cancer: A Meta-Analysis of Randomized Controlled Trials | 100% overlap with other reviews |

Abbreviations: CCA=corrected covered area; MA=meta-analysis; NMA=network meta-analysis; SR=systematic review

**References**

1. Abt E. Zinc Supplementation May Reduce the Effects of Oral Mucositis for Patients With Cancer Receiving Either Chemotherapy or Radiotherapy. *The journal of evidence-based dental practice*. 2020;20(4):101494. <https://doi.org/https://dx.doi.org/10.1016/j.jebdp.2020.101494>

2. Abt E. PROBIOTICS MAY LOWER THE RISK OF ORAL MUCOSITIS IN CANCER PATIENTS. *The journal of evidence-based dental practice*. 2021;21(4):101639. <https://doi.org/https://dx.doi.org/10.1016/j.jebdp.2021.101639>

3. Akbari S, Kariznavi E, Jannati M, Elyasi S, Tayarani-Najaran Z. Curcumin as a preventive or therapeutic measure for chemotherapy and radiotherapy induced adverse reaction: A comprehensive review. *Food and chemical toxicology : an international journal published for the British Industrial Biological Research Association*. 2020;145:111699. <https://doi.org/https://dx.doi.org/10.1016/j.fct.2020.111699>

4. Al-Hussaniy HA, Alburghaif AH, Alkhafaje Z, et al. Chemotherapy-induced cardiotoxicity: a new perspective on the role of Digoxin, ATG7 activators, Resveratrol, and herbal drugs. *Journal of medicine and life*. 2023;16(4):491-500. <https://doi.org/https://dx.doi.org/10.25122/jml-2022-0322>

5. Alcantara MJE, Pandy JGP, Sebastian MRC. Vitamin E in the treatment of chemotherapy and radiation-induced mucositis: A meta-analysis of randomized controlled trials. *Annals of Oncology*. 2020;31(Supplement 6):S1372-S1373. <https://doi.org/https://dx.doi.org/10.1016/j.annonc.2020.10.332>

6. Blackwood HA, Hall CC, Balstad TR, et al. A systematic review examining nutrition support interventions in patients with incurable cancer. *Supportive care in cancer : official journal of the Multinational Association of Supportive Care in Cancer*. 2020;28(4):1877-1889. <https://doi.org/https://dx.doi.org/10.1007/s00520-019-04999-4>

7. Bowen JM, Gibson RJ, Coller JK, et al. Systematic review of agents for the management of cancer treatment-related gastrointestinal mucositis and clinical practice guidelines. *Supportive Care in Cancer*. 2019;27(10):4011-4022. <https://doi.org/10.1007/s00520-019-04892-0>

8. Chaitanya NCSK, Shugufta K, Suvarna C, et al. Meta-analysis on the efficacy of zinc in oral mucositis during cancer chemo and/or radiotherapy-an evidence-based approach. *Journal of Nutritional Science and Vitaminology*. 2019;65(2):184-191. <https://doi.org/https://dx.doi.org/10.3177/jnsv.65.184>

9. Cogo E, Elsayed M, Liang V, et al. Are Supplemental Branched-Chain Amino Acids Beneficial During the Oncological Peri-Operative Period: A Systematic Review and Meta-Analysis. *Integr Cancer Ther*. Jan-Dec 2021;20:1534735421997551. <https://doi.org/10.1177/1534735421997551>

10. Cogo E, Elsayed ME, Liang V, et al. A systematic review of probiotics use during oncological surgery. *Journal of Complementary and Integrative Medicine*. 2023;20(2):eA54-eA55. <https://doi.org/https://dx.doi.org/10.1515/jcim-2020-2120>

11. Crichton M, Marshall S, Marx W, McCarthy AL, Isenring E. Efficacy of Ginger (Zingiber officinale) in Ameliorating Chemotherapy-Induced Nausea and Vomiting and Chemotherapy-Related Outcomes: A Systematic Review Update and Meta-Analysis. *J Acad Nutr Diet*. Dec 2019;119(12):2055-2068. <https://doi.org/10.1016/j.jand.2019.06.009>

12. Daugelaite G, Uzkuraityte K, Jagelaviciene E, Filipauskas A. Prevention and Treatment of Chemotherapy and Radiotherapy Induced Oral Mucositis. *Medicina (Kaunas, Lithuania)*. 2019;55(2)<https://doi.org/https://dx.doi.org/10.3390/medicina55020025>

13. Di Napoli R, Balzano N, Mascolo A, et al. What Is the Role of Nutraceutical Products in Cancer Patients? A Systematic Review of Randomized Clinical Trials. *Nutrients*. 2023;15(14)<https://doi.org/10.3390/nu15143249>

14. Elad S, Cheng KKF, Lalla RV, et al. MASCC/ISOO clinical practice guidelines for the management of mucositis secondary to cancer therapy. *Cancer*. 2020;126(19):4423-4431. <https://doi.org/https://dx.doi.org/10.1002/cncr.33100>

15. Emanuel A, Krampitz J, Rosenberger F, Kind S, Rotzer I. Nutritional Interventions in Pancreatic Cancer: A Systematic Review. *Cancers*. 2022;14(9)<https://doi.org/https://dx.doi.org/10.3390/cancers14092212>

16. Firoozbakhsh P, Ghaffarinejad Z, Arbabi M, Dokhani N, Alizadehasl A. Cardioprotective potential of botanical agents against anthracycline-induced cardiotoxicity. *Phytomedicine Plus*. 2024;4(2):100575. <https://doi.org/https://dx.doi.org/10.1016/j.phyplu.2024.100575>

17. Garczyk A, Kaliciak I, Drogowski K, et al. Influence of Probiotics in Prevention and Treatment of Patients Who Undergo Chemotherapy or/and Radiotherapy and Suffer from Mucositis, Diarrhoea, Constipation, Nausea and Vomiting. *Journal of Clinical Medicine*. 2022;11(12):3412. <https://doi.org/https://dx.doi.org/10.3390/jcm11123412>

18. Gardiner B, Wardill H, O'Connor G, Hargrave D, Frost G, Lett A. THE EFFICACY OF FIBRE AND PREBIOTIC INTERVENTIONS ON CLINICAL OUTCOMES IN CANCER AND HAEMATOPOIETIC STEM CELL THERAPIES: SYSTEMATIC LITERATURE REVIEW. *Pediatric Blood and Cancer*. 2022;69(Supplement 5):S350. <https://doi.org/https://dx.doi.org/10.1002/pbc.29952>

19. Gholamalizadeh M, Ardekanizadeh NH, Aghakhaninejad Z, et al. The effects of dietary supplements in patients with cervical cancer: a comprehensive systematic review. *European journal of obstetrics & gynecology and reproductive biology: X*. 2023;19:100217. <https://doi.org/10.1016/j.eurox.2023.100217>

20. Ginzac A, Dubois S, Hager MO, et al. Quality of life for older patients with cancer: a review of the evidence supporting melatonin use. *Aging Clinical and Experimental Research*. 2020;32(12):2459-2468. <https://doi.org/https://dx.doi.org/10.1007/s40520-020-01532-0>

21. Jangid S, Verma A, Pruthi J, Prasanna R, Rai MK. Non-Pharmacological Strategies for Chemotherapy-Induced Peripheral Neuropathy: A Comprehensive Review. *Value in Health*. 2023;26(12 Supplement):S28. <https://doi.org/https://dx.doi.org/10.1016/j.jval.2023.09.147>

22. Jayaraj R, Kumaraswamy C, Shetty S, et al. Clinical approaches to interpreting the findings of systematic review and meta-analysis of the effectiveness of probiotics in the prevention and treatment of Cancer Therapy-Induced Oral Mucositis (CTIOM). *Oral Oncology*. 2020;104:104622. <https://doi.org/https://dx.doi.org/10.1016/j.oraloncology.2020.104622>

23. Kalisch A, Rostock M, Stapf A, Steinmann D, Horneber M. Complementary therapies for the prevention and management of chemotherapy-induced peripheral neuropathy. *Oncology Research and Treatment*. 2019;42(Supplement 1):8-9. <https://doi.org/https://dx.doi.org/10.1159/000497740>

24. Karaboga Arslan A, Uzunhisarcikli E, Yerer M, Bishayee A. The golden spice curcumin in cancer: A perspective on finalized clinical trials during the last 10 years. *Journal of Cancer Research and Therapeutics*. 2022;18(1):19-26. <https://doi.org/https://dx.doi.org/10.4103/jcrt.JCRT_1017_20>

25. Kemp E, Lawn S, Clark RA, et al. Interventions to manage cardiovascular disease risk in cancer patients and survivors: An umbrella review. *Asia-Pacific Journal of Clinical Oncology*. 2020;16(SUPPL 8):136. <https://doi.org/https://dx.doi.org/10.1111/ajco.13498>

26. Khamdi S, Matangkasombut O, Lam-Ubol A. Non-pharmacologic interventions for management of radiation-induced dry mouth: A systematic review. *Oral Diseases*. 2023;<https://doi.org/https://dx.doi.org/10.1111/odi.14804>

27. Li P, Wang Q, Liu L, et al. The Role of Complementary and Alternative Medicine on Cancer-Related Fatigue in Adults: An Overview of Systematic Reviews. *Integrative Cancer Therapies*. 2023;22<https://doi.org/https://dx.doi.org/10.1177/15347354231188947>

28. Lian S, Zhang X, Zhang Y, Zhao Q. Pyridoxine for prevention of hand-foot syndrome caused by chemotherapy agents: a meta-analysis. *Clinical and Experimental Dermatology*. 2021;46(4):629-635. <https://doi.org/https://dx.doi.org/10.1111/ced.14486>

29. Lim S, Park S, Koyanagi A, et al. Effects of exogenous melatonin supplementation on health outcomes: An umbrella review of meta-analyses based on randomized controlled trials. *Pharmacological Research*. 2022;176:106052. <https://doi.org/https://dx.doi.org/10.1016/j.phrs.2021.106052>

30. Mansouri K, Rasoulpoor S, Daneshkhah A, et al. Clinical effects of curcumin in enhancing cancer therapy: A systematic review. *BMC Cancer*. 2020;20(1):791. <https://doi.org/https://dx.doi.org/10.1186/s12885-020-07256-8>

31. Mezerji NMG, Moghimbeigi A, Eghbalian M, Kopaei MR. Investigating the effect of herbal antioxidants on the process of colon cancer treatment: A systematic review. *Cogent Medicine*. 2020;7(1):1735682. <https://doi.org/https://dx.doi.org/10.1080/2331205X.2020.1735682>

32. Mirzaei Dahka S, Afsharfar M, Tajaddod S, et al. Impact of Curcumin Supplementation on Radiation Dermatitis Severity: A Systematic Review and Meta-Analysis of Randomized Controlled Trials. *Asian Pacific journal of cancer prevention : APJCP*. 2023;24(3):783-789. <https://doi.org/https://dx.doi.org/10.31557/APJCP.2023.24.3.783>

33. Mora DC, Overvag G, Jong MC, et al. Complementary and alternative medicine modalities used to treat adverse effects of anti-cancer treatment among children and young adults: a systematic review and meta-analysis of randomized controlled trials. *BMC Complementary Medicine and Therapies*. 2022;22(1):97. <https://doi.org/https://dx.doi.org/10.1186/s12906-022-03537-w>

34. Moutabian H, Ghahramani-Asl R, Mortezazadeh T, et al. The cardioprotective effects of nano-curcumin against doxorubicin-induced cardiotoxicity: A systematic review. *BioFactors (Oxford, England)*. 2022;48(3):597-610. <https://doi.org/https://dx.doi.org/10.1002/biof.1823>

35. Muhamad R, Akrivaki A, Papagiannopoulou G, Zavridis P, Zis P. The Role of Vitamin B6 in Peripheral Neuropathy: A Systematic Review. *Nutrients*. 2023;15(13):2823. <https://doi.org/https://dx.doi.org/10.3390/nu15132823>

36. Muszyński T, Polak K, Frątczak A, Miziołek B, Bergler-Czop B, Szczepanik A. Vitamin D-The Nutritional Status of Post-Gastrectomy Gastric Cancer Patients-Systematic Review. *Nutrients*. 2022;14(13)<https://doi.org/10.3390/nu14132712>

37. Najafi M, Hooshangi Shayesteh MR, Mortezaee K, Farhood B, Haghi-Aminjan H. The role of melatonin on doxorubicin-induced cardiotoxicity: A systematic review. *Life Sciences*. 2020;241:117173. <https://doi.org/https://dx.doi.org/10.1016/j.lfs.2019.117173>

38. Nayeri ND, Bakhshi F, Khosravi A, Najafi Z. The Effect of Complementary and Alternative Medicines on Quality of Life in Patients with Breast Cancer: A Systematic Review. *Indian Journal of Palliative Care*. 2020;26(1):95-104. <https://doi.org/10.4103/IJPC.IJPC_183_19>

39. Parsons HM, Forte ML, Abdi HI, et al. Nutrition as prevention for improved cancer health outcomes: a systematic literature review. *JNCI Cancer Spectrum*. 2023;7(3)<https://doi.org/https://dx.doi.org/10.1093/jncics/pkad035>

40. Patel P, Wang JY, Mineroff J, Jagdeo J. Evaluation of curcumin for dermatologic conditions: a systematic review. *Archives of Dermatological Research*. 2024;316(1):37. <https://doi.org/https://dx.doi.org/10.1007/s00403-023-02754-8>

41. Perez-Bilbao T, Alonso-Duenas M, Peinado AB, San Juan AF. Effects of Combined Interventions of Exercise and Diet or Exercise and Supplementation on Breast Cancer Patients: A Systematic Review. *Nutrients*. 2023;15(4)<https://doi.org/https://dx.doi.org/10.3390/nu15041013>

42. Roberts KE, Adsett IT, Rickett K, Conroy SM, Chatfield MD, Woodward NE. Systemic therapies for preventing or treating aromatase inhibitor-induced musculoskeletal symptoms in early breast cancer. *Cochrane Database Syst Rev*. Jan 10 2022;1(1):Cd013167. <https://doi.org/10.1002/14651858.CD013167.pub2>

43. Srinivasaraghavan N, Das N, Balakrishnan K, Rajaram S. Effect of Whey Protein Supplementation on Perioperative Outcomes in Patients with Cancer-A Systematic Review and Meta-Analysis (PROSPERO 2020: CRD42020188666). *Nutrition and Cancer*. 2021:1-14. <https://doi.org/10.1080/01635581.2021.2020302>

44. Thomsen M, Vitetta L. Zinc deficits, mucositis, and mucosal macrophage perturbation: is there a relationship? *Current opinion in clinical nutrition and metabolic care*. 2019;22(5):365-370. <https://doi.org/https://dx.doi.org/10.1097/MCO.0000000000000588>

45. Ukovic B, Porter J. Nutrition interventions to improve the appetite of adults undergoing cancer treatment: a systematic review. *Supportive care in cancer : official journal of the Multinational Association of Supportive Care in Cancer*. 2020;28(10):4575-4583. <https://doi.org/https://dx.doi.org/10.1007/s00520-020-05475-0>

46. Vogel M, Franik S, Kiesel L. Phytoestrogen Treatment for Menopausal Vasomotor Symptoms after Breast Cancer. *Breast Care*. 2023;18(3):158-163. <https://doi.org/10.1159/000529695>

47. Wiese F, Kutschan S, Doerfler J, et al. Green tea and green tea extract in oncological treatment: A systematic review. *Int J Vitam Nutr Res*. Feb 2023;93(1):72-84. <https://doi.org/10.1024/0300-9831/a000698>

48. Wolyniuk L, Youssef AN, Louie AV, Mutsaers A. NUTRITIONAL INTERVENTIONS TO SUPPORT LUNG CANCER PATIENTS UNDERGOING THORACIC RADIATION: A SYSTEMATIC REVIEW. *Radiotherapy and Oncology*. 2023;186(Supplement 1):S53-S54. <https://doi.org/https://dx.doi.org/10.1016/S0167-8140%2823%2989907-8>

49. Wu CF, Wu HJ, Shih CL, Yeh TP, Ma WF. Efficacy of turmeric in the treatment of oral mucositis in patients with head and neck cancer after radiotherapy or chemoradiotherapy: a systematic review and meta-analysis. *Front Pharmacol*. 2024;15:1363202. <https://doi.org/10.3389/fphar.2024.1363202>

50. Yarom N, Hovan A, Bossi P, et al. Systematic review of natural and miscellaneous agents for the management of oral mucositis in cancer patients and clinical practice guidelines-part 1: vitamins, minerals, and nutritional supplements. *Supportive care in cancer : official journal of the Multinational Association of Supportive Care in Cancer*. 2019;27(10):3997-4010. <https://doi.org/https://dx.doi.org/10.1007/s00520-019-04887-x>

51. Yarom N, Hovan A, Bossi P, et al. Correction to: Systematic review of natural and miscellaneous agents, for the management of oral mucositis in cancer patients and Clinical Practice Guidelines - Part 1: vitamins, minerals and nutritional supplements (Supportive Care in Cancer, (2019), 27, 10, (3997-4010), 10.1007/s00520-019-04887-x). *Supportive Care in Cancer*. 2021;29(7):4175-4176. <https://doi.org/https://dx.doi.org/10.1007/s00520-021-06141-9>

52. Zhang XY, Yang KL, Li Y, et al. Can Dietary Nutrients Prevent Cancer Chemotherapy-Induced Cardiotoxicity? An Evidence Mapping of Human Studies and Animal Models. *Frontiers in Cardiovascular Medicine*. 2022;9:921609. <https://doi.org/https://dx.doi.org/10.3389/fcvm.2022.921609>

53. Auttara-Atthakorn A, Sungmala J, Anothaisintawee T, Reutrakul S, Sriphrapradang C. Prevention of salivary gland dysfunction in patients treated with radioiodine for differentiated thyroid cancer: A systematic review of randomized controlled trials. *Front Endocrinol (Lausanne)*. 2022;13:960265. <https://doi.org/10.3389/fendo.2022.960265>

54. Chan CWH, Tai D, Kwong S, Chow KM, Chan DNS, Law BMH. The Effects of Pharmacological and Non-Pharmacological Interventions on Symptom Management and Quality of Life among Breast Cancer Survivors Undergoing Adjuvant Endocrine Therapy: A Systematic Review. *Int J Environ Res Public Health*. Apr 24 2020;17(8)<https://doi.org/10.3390/ijerph17082950>

55. Crichton M, Davidson AR, Innerarity C, et al. Orally consumed ginger and human health: an umbrella review. *Am J Clin Nutr*. Jun 7 2022;115(6):1511-1527. <https://doi.org/10.1093/ajcn/nqac035>

56. de Menêses AG, Normando AGC, Porto de Toledo I, Reis PED, Guerra ENS. Effects of oral supplementation in the management of oral mucositis in cancer patients: A meta-analysis of randomized clinical trials. *J Oral Pathol Med*. Feb 2020;49(2):117-125. <https://doi.org/10.1111/jop.12901>

57. Dharman S, G M, Shanmugasundaram K, Sampath RK. A Systematic Review and Meta-Analysis on the Efficacy of Curcumin/Turmeric for the Prevention and Amelioration of Radiotherapy/Radiochemotherapy Induced Oral Mucositis in Head and Neck Cancer Patients. *Asian Pac J Cancer Prev*. Jun 1 2021;22(6):1671-1684. <https://doi.org/10.31557/apjcp.2021.22.6.1671>

58. Edwards A, Santos C, Chen A-Y, Bauer J. Nutritional interventions for oral mucositis: a systematic literature review. *Nutrition & Dietetics*. 2021/02/01 2021;78(1):101-114. <https://doi.org/https://doi.org/10.1111/1747-0080.12656>

59. Frey-Furtado L, Magalhães I, Azevedo MJ, Sampaio-Maia B. The Role of Biotics as a Therapeutic Strategy for Oral Mucositis - A Systematic Review. *Probiotics Antimicrob Proteins*. Aug 2024;16(4):1313-1326. <https://doi.org/10.1007/s12602-023-10116-z>

60. Guo PP, Li P, Zhang XH, et al. Complementary and alternative medicine for natural and treatment-induced vasomotor symptoms: An overview of systematic reviews and meta-analyses. *Complement Ther Clin Pract*. Aug 2019;36:181-194. <https://doi.org/10.1016/j.ctcp.2019.07.007>

61. Hao J, Zhu X, Bensoussan A. Effects of Nonpharmacological Interventions in Chemotherapy-Induced Peripheral Neuropathy: An Overview of Systematic Reviews and Meta-Analyses. *Integr Cancer Ther*. Jan-Dec 2020;19:1534735420945027. <https://doi.org/10.1177/1534735420945027>

62. Heilfort L, Kutschan S, Dörfler J, et al. A Systematic Review of the Benefit of B-Vitamins as a Complementary Treatment in Cancer Patients. *Nutr Cancer*. 2023;75(1):33-47. <https://doi.org/10.1080/01635581.2022.2098348>

63. Jafari-Koulaee A, Bagheri-Nesami M. The effect of melatonin on sleep quality and insomnia in patients with cancer: a systematic review study. *Sleep Med*. Jun 2021;82:96-103. <https://doi.org/10.1016/j.sleep.2021.03.040>

64. Kao YS, Wu YC, Wu MY, Wu PL, Lu LY, Hung CH. Topical Prevention of Radiation Dermatitis in Breast Cancer Patients: A Network Meta-analysis of Randomized Controlled Trials. *In Vivo*. May-Jun 2023;37(3):1346-1357. <https://doi.org/10.21873/invivo.13216>

65. Lee CC, Chen YW, Kang YN, et al. Efficacy of natural products in preventing oral mucositis resulting from cancer therapies: A network meta-analysis of randomized controlled trials. *Crit Rev Oncol Hematol*. Jul 2024;199:104373. <https://doi.org/10.1016/j.critrevonc.2024.104373>

66. Liu YC, Wu CR, Huang TW. Preventive Effect of Probiotics on Oral Mucositis Induced by Cancer Treatment: A Systematic Review and Meta-Analysis. *Int J Mol Sci*. Oct 31 2022;23(21)<https://doi.org/10.3390/ijms232113268>

67. Lu Y, Luo X, Yang D, et al. Effects of probiotic supplementation on related side effects after chemoradiotherapy in cancer patients. *Front Oncol*. 2022;12:1032145. <https://doi.org/10.3389/fonc.2022.1032145>

68. Minervini G, Franco R, Marrapodi MM, et al. Probiotics in the Treatment of Radiotherapy-Induced Oral Mucositis: Systematic Review with Meta-Analysis. *Pharmaceuticals (Basel)*. Apr 27 2023;16(5)<https://doi.org/10.3390/ph16050654>

69. Normando AGC, de Menêses AG, de Toledo IP, et al. Effects of turmeric and curcumin on oral mucositis: A systematic review. *Phytother Res*. May 2019;33(5):1318-1329. <https://doi.org/10.1002/ptr.6326>

70. Osouli-Tabrizi S, Mehdizadeh A, Naghdi M, Sanaat Z, Vahed N, Farshbaf-Khalili A. The effectiveness of omega-3 fatty acids on health outcomes in women with breast cancer: A systematic review. *Food Sci Nutr*. Aug 2023;11(8):4355-4371. <https://doi.org/10.1002/fsn3.3409>

71. Peng TR, Tsai FP, Wu TW. Effects of various treatments for preventing oral mucositis in cancer patients: A network meta-analysis. *PLoS One*. 2022;17(12):e0278102. <https://doi.org/10.1371/journal.pone.0278102>

72. Picó-Monllor JA, Mingot-Ascencao JM. Search and Selection of Probiotics That Improve Mucositis Symptoms in Oncologic Patients. A Systematic Review. *Nutrients*. Oct 1 2019;11(10)<https://doi.org/10.3390/nu11102322>

73. Ramos E, Egea J, López-Muñoz F, Gil-Martín E, Romero A. Therapeutic Potential of Melatonin Counteracting Chemotherapy-Induced Toxicity in Breast Cancer Patients: A Systematic Review. *Pharmaceutics*. May 30 2023;15(6)<https://doi.org/10.3390/pharmaceutics15061616>

74. Rodriguez-Arrastia M, Martinez-Ortigosa A, Rueda-Ruzafa L, Folch Ayora A, Ropero-Padilla C. Probiotic Supplements on Oncology Patients' Treatment-Related Side Effects: A Systematic Review of Randomized Controlled Trials. *Int J Environ Res Public Health*. Apr 17 2021;18(8)<https://doi.org/10.3390/ijerph18084265>

75. Shu Z, Li P, Yu B, Huang S, Chen Y. The effectiveness of probiotics in prevention and treatment of cancer therapy-induced oral mucositis: A systematic review and meta-analysis. *Oral Oncol*. Mar 2020;102:104559. <https://doi.org/10.1016/j.oraloncology.2019.104559>

76. Shuai T, Tian X, Shi B, et al. Prophylaxis With Oral Zinc Sulfate Against Radiation Induced Oral Mucositis in Patients With Head and Neck Cancers: A Systematic Review and Meta-Analysis of Four Randomized Controlled Trials. *Front Oncol*. 2019;9:165. <https://doi.org/10.3389/fonc.2019.00165>

77. Van de Roovaart HJ, Stevens MM, Goodridge AE, et al. Safety and efficacy of vitamin B in cancer treatments: A systematic review. *J Oncol Pharm Pract*. Apr 2024;30(3):451-463. <https://doi.org/10.1177/10781552231178686>

78. Wen S, Brito L, Santander J, Conteras G. Update on the treatment of chemotherapy and radiotherapy-induced buccal mucositis: a systematic review. *Acta Odontol Latinoam*. Apr 29 2023;36(1):3-14. <https://doi.org/10.54589/aol.36/1/3>

79. Wilairat P, Kengkla K, Kaewpanan T, et al. Comparative efficacy and safety of interventions for preventing chemotherapy-induced oral mucositis in adult cancer patients: a systematic review and network meta-analysis. *Eur J Hosp Pharm*. Mar 2020;27(2):103-110. <https://doi.org/10.1136/ejhpharm-2018-001649>

80. Yu YT, Deng JL, Jin XR, Zhang ZZ, Zhang XH, Zhou X. Effects of 9 oral care solutions on the prevention of oral mucositis: a network meta-analysis of randomized controlled trials. *Medicine (Baltimore)*. Apr 2020;99(16):e19661. <https://doi.org/10.1097/md.0000000000019661>

81. Zhang L, Tang G, Wei Z. Prophylactic and Therapeutic Effects of Curcumin on Treatment-Induced Oral Mucositis in Patients with Head and Neck Cancer: A Meta-Analysis of Randomized Controlled Trials. *Nutr Cancer*. 2021;73(5):740-749. <https://doi.org/10.1080/01635581.2020.1776884>
